# Clinical predictors of severe dengue: a systematic review and meta-analysis

**DOI:** 10.1186/s40249-021-00908-2

**Published:** 2021-10-09

**Authors:** Tsheten Tsheten, Archie C. A. Clements, Darren J. Gray, Ripon K. Adhikary, Luis Furuya-Kanamori, Kinley Wangdi

**Affiliations:** 1 Department of Global Health, Research School of Population Health, College of Health and Medicine, Australian National University, Canberra, Australia; 2 Royal Centre for Disease Control, Ministry of Health, Thimphu, Bhutan; 3 Telethon Kids Institute, Nedlands, Australia; 4 Curtin University, Perth, Australia; 5 UQ Centre for Clinical Research, The University of Queensland, Herston, QLD Australia

**Keywords:** Severe dengue, Meta-analysis, Risk factor, Warning sign

## Abstract

**Background:**

Severe dengue is a life-threatening complication; rapid identification of these cases, followed by adequate management is crucial to improve the clinical prognosis. Therefore, this study aimed to identify risk factors and predictors of severe dengue.

**Methods:**

A literature search for studies reporting risk factors of severe dengue among individuals with dengue virus infection was conducted in PubMed, Scopus and Web of Science database from inception to December 31, 2020. Pooled odds ratios (*ORs*) for patients’ demographic characteristics, co-morbidities, and warning signs were estimated using an inverse variance heterogeneity model.

**Results:**

We included 143 articles in the meta-analysis from a total of 13 090 articles retrieved from the literature search. The risk factors of severe dengue were: being a child [*OR* = 1.96; 95% confidence interval (*CI*): 1.22–3.13], secondary infection (*OR* = 3.23; 95% *CI*: 2.28–4.57), and patients with pre-existing diabetes (*OR* = 2.88; 95% *CI*: 1.72–4.81) and renal disease (*OR* = 4.54; 95% *CI*: 1.55–13.31). Warning signs strongly associated with severe disease were increased haematocrit with a concurrent decrease in platelet count (*OR* = 5.13; 95% *CI*: 1.61–16.34), abdominal pain (*OR* = 2.00; 95% *CI*: 1.49–2.68), lethargy (*OR* = 2.73; 95% *CI*: 1.05–7.10), vomiting (*OR* = 1.80; 95% *CI*: 1.43–2.26), hepatomegaly (*OR* = 5.92; 95% *CI*: 3.29–10.66), ascites (*OR* = 6.30; 95% *CI*: 3.75–10.60), pleural effusion (*OR* = 5.72; 95% *CI*: 3.24–10.10) and melena (*OR* = 4.05; 95% *CI*: 1.64–10.00).

**Conclusions:**

Our meta-analysis identified children, secondary infection, diabetes and renal disease(s) as important predictors of severe dengue. Our finding also supports the predictive ability of the WHO warning signs to identify severe dengue. These findings are useful for clinicians to identify severe dengue for management and timely interventions.

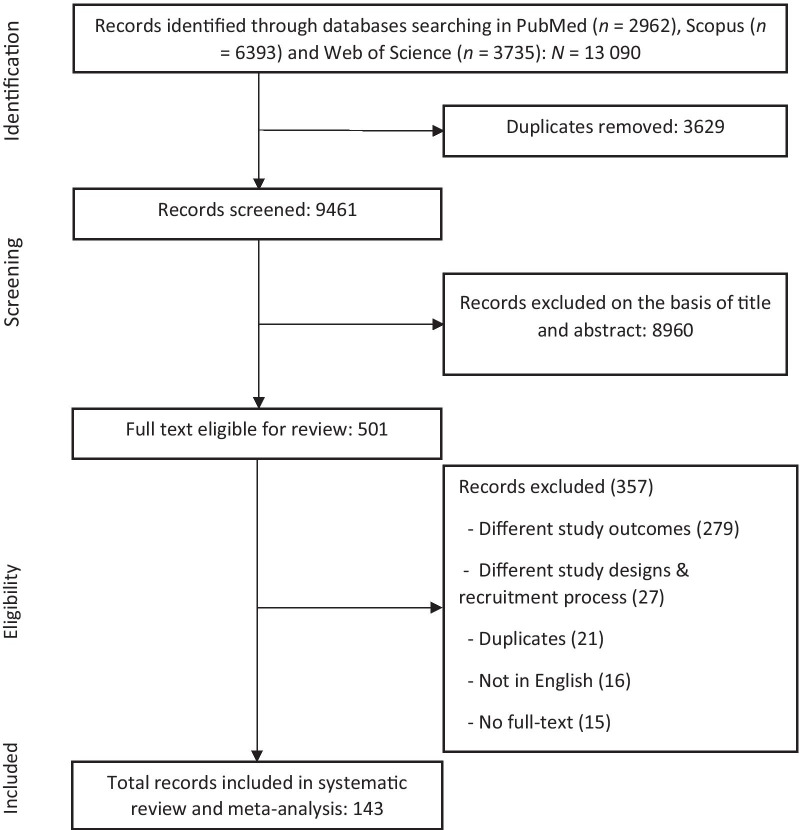

**Supplementary Information:**

The online version contains supplementary material available at 10.1186/s40249-021-00908-2.

## Background

In 2010, it was estimated that there were 390 million dengue infections, of which 96 million manifested clinically [1] with severe dengue resulting in 21 000 deaths worldwide [2]. Asia bears 70% of this global burden [1]. The incidence of dengue has surged dramatically with an eightfold increase over the last two decades, from 505 430 cases in 2000 to over 2.4 million in 2010, and 4.2 million in 2019 [3]. The increase in dengue incidence has been associated with explosive outbreaks and geographical expansion to new areas [3].

Dengue is an arboviral infection caused by a dengue virus (DENV) belonging to the Flaviviridae family. Four antigenically and genetically distinct DENV serotypes (DENV1–4) have been described to co-circulate around the world and cause human infections [4]. The infection leads to a wide spectrum of clinical manifestations from asymptomatic infection to life threatening severe dengue or dengue shock syndrome (DSS) [1]. In many Asian countries, severe dengue is the leading cause of hospitalization among children and the case fatality rate (CFR) is about 5% on average [5].

There is no specific treatment and the dengue vaccine [CYD-TDV (Dengvaxia^®^)] is licensed only in 20 countries [6]. The vaccine is not yet approved for younger children due to low efficacy and safety reasons [7]. In a randomized controlled, multicentre, phase III trial, the efficacy of CYD-TDV was reported at ~ 56% against virologically confirmed dengue among children in countries in the Asia–Pacific region [8]. Only adults aged 9–45 years living in an area of ≥ 70% dengue prevalence, and whose serostatus is positive for past dengue infection are recommended for immunization [6]. Due to the challenges associated with the need to collect information on the burden and seroprevalence profiles of the local population, and the recent reports of vaccine-related severe dengue and deaths, the use of the dengue vaccines is not widespread [9]. Therefore, rapid identification of severe cases and appropriate clinical management remains the mainstay to avoid dengue-related case fatalities. This includes monitoring for plasma leakage and initiating intravenous fluid replacement to prevent shock and death [10]. A rational approach of case management through proper understanding of the determinants of severe dengue is key to improving clinical outcomes [11].

This systematic review and meta-analysis aimed to identify predictors of severe dengue. Such knowledge will be useful to clinicians for targeting at-risk groups of severe dengue for initiating prompt interventions to save lives.

## Methods

### Search strategy

The methods and results of the systematic review and meta-analysis are reported in accordance with the recommendations of the Preferred Reporting Items for Systematic Reviews and Meta-Analyses (PRISMA) guidelines (Additional file 1) [12]. No protocol was registered for this systematic review and meta-analysis.

Three databases, PubMed, Scopus and Web of Science, were searched from inception to December 31, 2020, for relevant articles. Key search terms were “dengue”, “dengue haemorrhagic fever”, “dengue shock syndrome” or “severe dengue”. The detailed search strategy is provided in Additional file 2. In addition, a backward citation search using the reference lists of relevant studies were reviewed for additional studies that may not have been captured using the search terms.

### Eligibility criteria

This review was undertaken to identify predictors of severe dengue based on the patient’s demographic characteristics, comorbidities, and presentation of warning signs; therefore, the inclusion criteria were: (1) observational studies (cross-sectional, case control, or cohort study designs) conducted in humans; (2) which compared severe dengue and non-severe dengue cases; and (3) reported patients’ demographic characteristics (i.e., age, sex, ethnicity, socio-economic class, region/location, and primary or secondary dengue infection), co-morbidities [i.e., asthma, chronic obstructive pulmonary disease (COPD), visual impairment, cardiovascular diseases (CVD), diabetes, obesity and overweight, hearing loss, cancer, oral health, alcohol use disorder, and haemoglobin disorders like thalassemia and sickle cell disease], and/or clinical warning signs [i.e., abdominal pain, vomiting, enlarged liver size, pleural effusion, ascites, gum bleeding, epistaxis, lethargy, melena, increase in haematocrit with concurrent decrease in platelet count, gastrointestinal (GI) bleeding, hematemesis and skin bleeding]. Exclusion criteria included: (1) case reports, case series, reviews, or letters; (2) in vitro and animal studies; (3) conference presentations; and (4) studies where patient outcomes were not separated into severe and non-severe dengue.

The classification of the severity of dengue of the selected studies was done either with the World Health Organization (WHO) 1997 or the revised WHO 2009 dengue case classification. The WHO 1997 dengue case classification categorized dengue into dengue fever (DF), dengue haemorrhagic fever (DHF) (i.e., grade I & II) and dengue shock syndrome (DSS) (i.e., grade III & IV) [5]. While the WHO 2009 dengue case classification categorized dengue into dengue without warning signs (DWoWS), dengue with warning signs (DWWS), and severe dengue (SD) [10]. In this review, we defined severe dengue as DSS according to the WHO 1997 dengue case classification and SD according to WHO 2009 dengue case classification. A detailed description of the WHO 1997 and 2009 dengue case classification along with the case definition of severe dengue used in this study are presented in Table 1.

**Table 1:** Dengue severity stratification according to 1997 and 2009 World Health Organization (WHO) guidelines

1997 WHO classification			2009 WHO classification		
Dengue fever	DHF (Grade 1 and 2)	DSS (Grade 3 and 4)	DWoWS	DWWS	SD
Acute febrile illness with ≥ 2 of the following symptoms: Headache Retro-orbital pain Myalgia Arthralgia Rash Haemorrhage manifestations Leukopenia	Following must be all present: Fever or history of fever Haemorrhage tendencies (as manifested by a positive tourniquet test, petechiae/purpura/ecchymoses, mucosal bleeding) Thrombocytopenia Plasma leakage (a rise in the haematocrit, pleural effusion, ascites)	All four criteria of DHF plus evidence of circulatory failure evidenced by: Rapid and weak pulse Narrow pulse pressure (< 20 mm Hg) Hypotension Cold clammy skin and restlessness	Fever and two of the following symptoms: Nausea or vomiting Rash Bodyaches Positive tourniquet test Leukopenia	Similar features of DWoWS with the following warning signs: Abdominal pain Persistent vomiting Lethargy or restlessness Liver enlargement Increase in HCT and decrease in platelet count	Patients with any of the following features: Severe plasma leakage leading to Shock, fluid accumulation with respiratory distress Severe bleeding Severe organ impairment

*DHF* dengue haemorrhagic fever, *DSS* dengue shock syndrome, *DWoWS* dengue without warning signs, *DWWS* dengue with warning signs, *SD* severe dengue, *HCT* haematocrit

*Case definition of severe dengue used in the meta-analysis is shaded in grey

### Selection of studies and data extraction

All retrieved articles from the three databases (PubMed, Scopus and Web of Science) were imported into EndNote X7.7.1 (Clarivate Analytics, Philadelphia, PA, USA) and duplicates were removed. Then studies were screened by title and abstract in Rayyan (http://rayyan.qcri.org/). Using Rayyan, articles selected by title and abstract also underwent full text screening for the final selection. The screening process was conducted by two independent reviewers (TT and RKA) and any discrepancies during the selection of studies were resolved through discussion and consensus following independent evaluation by another author (KW).

The same two reviewers (TT and RKA) extracted the data of the eligible articles. Differences in the extracted data were resolved by consensus between the reviewers. The following information was extracted: name of the first author, WHO dengue case classification type (guideline 1997 or 2009), country name, recruitment time, study design/size, study population (children, adults or mixed), median/mean age, infection type (primary or secondary), warning signs (i.e., abdominal pain, persistent vomiting, clinical fluid accumulation, mucosal bleed, lethargy, liver enlargement, and increase in haematocrit with a concurrent decrease in platelets), co-morbidities (i.e., asthma, COPD, CVD, hypertension, diabetes, obesity, cancer, sickle cell disease), and the severity of disease (severe and non-severe dengue). When available, adjusted estimates were extracted, otherwise unadjusted estimates were calculated.

### Quality assessment

The quality of the studies was assessed using the MethodologicAl STandards for Epidemiological Research (MASTER) scale [13]. This scale has 36 bias safeguards that were categorized into seven methodological standards or equivalence [13]. These standards reflect initial and ongoing equivalence in equal recruitment, equal retention, equal ascertainment, equal implementation, equal prognosis, sufficient analysis and temporal precedence. The studies were rated as ‘1” or “0” depending on the presence or absence of each of these safeguard items. Safeguards not relevant to the studies were rated “0”. Similar to the screening and data extraction process, two independent reviewers (TT & RKA) conducted the assessment and any discrepancies were resolved by the consensus and involvement of another author (KW).

### Data analysis

The pooled odds ratios (*OR*s) with 95% confidence intervals (*CI*) comparing severe and non-severe dengue for each predictor was estimated using the inverse variance heterogeneity (IVhet) model [14]. Heterogeneity between studies was assessed using the Cochran Q and the *I*^*2*^ test statistics. Levels of heterogeneity were categorized according to the *I*^*2*^ index as low (< 25%), low to moderate (25% to < 50%), moderate to high (50% to < 75%) or high (≥ 75%). The same Cochran Q statistic was used to assess heterogeneity in the sub-group analysis.

Sub-group analyses were conducted to compare the differential effect based on (1) WHO dengue case classification of disease severity (1997 vs 2009), and (2) children and adults to identify risk factors specific to an age group. We defined participants under the age of 20 years as children and as adults otherwise. This classification was based on the definition in the studies, with some studies reporting 19 years as children. Studies reporting only children or adults were excluded from the age predictor analysis. A minimum of four studies per strata was required for sub-group analysis.

For sensitivity analysis, a bias-adjusted (quality-effect model) meta-analysis was performed using the score generated from the MASTER Scale. The scores of all safeguards generated as described above were added and converted into a relative rank between 0 and 1 by dividing the cumulative score of each study by the highest score. We included these quality ranks into the model to estimate bias-adjusted pooled effect sizes as a sensitivity analysis [15].

The publication bias was assessed using the Doi plot and LFK index bias [16]. LFK values beyond ± 1 were considered to be indicative of asymmetry and suggest the presence of publication bias [16]. The analysis was performed in the statistical program Stata 16 (College Station, TX: StataCorp LLC) using *metan* and *lfk* modules.

## Results

### Literature search

**Fig. 1:**
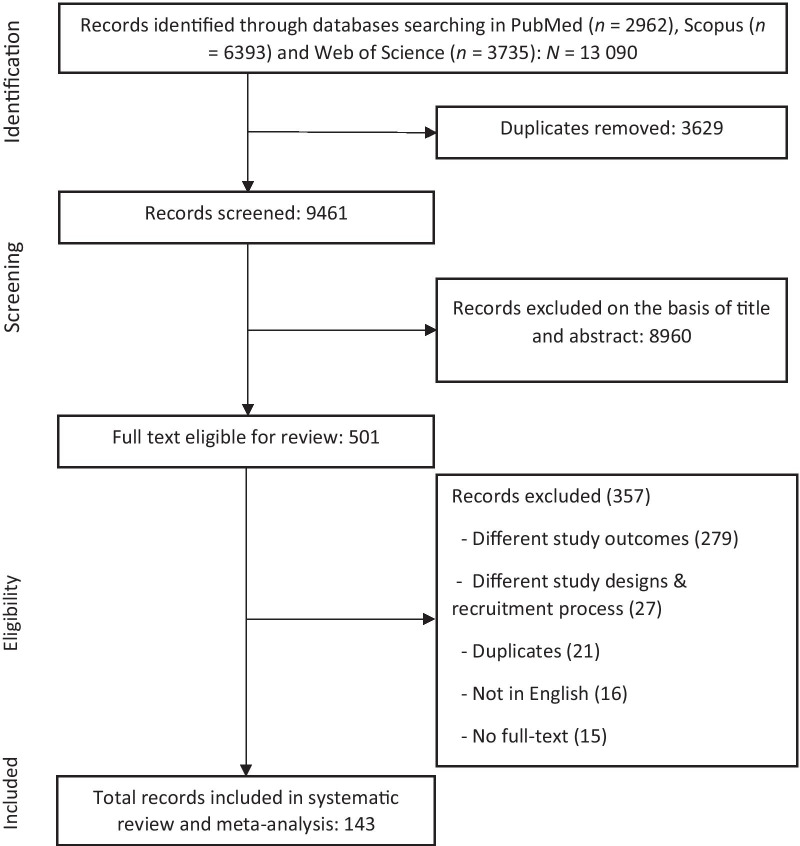
Screening and selection of studies

A total of 13 090 records were retrieved from the initial search. After removing 3629 duplicates, 9461 records were screened by titles and abstracts. Subsequently, 501 articles were included for full-text review, of which 143 articles remained and were included in the systematic review and meta-analysis (Fig. 1). Studies included in this study are presented in Additional file 3.

### Characteristics of the studies

Included studies were reported from the WHO regions as follows: South-East Asia (*n* = 74, 51.8%), Western Pacific (*n* = 34, 23.1%), Americas (*n* = 26, 18.2%), Eastern Mediterranean (*n* = 7, 4.9%), Europe (*n* = 2, 1.4%) and Africa (*n* = 1, 0.7%), respectively. Most of the studies were cross-sectional (*n* = 81, 56.6%) followed by cohort (*n* = 36, 25.2%) and case–control studies (*n* = 26, 18.2%). In 59 studies, only children were included, while 36 studies reported only adults, both children and adults were reported in 47 studies, and one study did not provide information on the age of the participants. Dengue severity was classified using the WHO 2009 dengue case classification in 85 (59.4%) studies, while the rest used the WHO 1997 dengue case classification (Table 2).

**Table 2:** Characteristics of the studies included to assess the demographic characteristics, co-morbidities and clinical warning signs of severe dengue

Parameters	Frequency (*n*)	Percentage (%)
WHO Classification type		
1997	58	40.6
2009	85	59.4
WHO region		
South-East Asia	74	51.8
Western Pacific	33	23.1
American	26	18.2
Eastern Mediterranean	7	4.9
European	2	1.4
Africa	1	0.7
Study design		
Case control	26	18.2
Cohort	36	25.2
Cross-sectional	81	56.6
Study population		
Children	59	41.3
Adults	36	25.2
Mixed	47	32.9
No information	1	0.7
Recruitment year		
1990–2010	78	54.6
2011–2019	64	44.8
No information	1	0.7
Laboratory diagnosis		
ELISA	96	67.1
PCR	49	34.3
RDT	27	18.9
HI	10	7.0
Viral culture	4	2.8
Immunohistochemistry	2	1.4
Immunofluorescence assay	1	0.7
Neutralization test	1	0.7
Dotblot immuno assay	1	0.7
No information	13	9.1

*ELISA* enzyme linked immunosorbent assay, *PCR* polymerase chain reaction, *RDT* rapid diagnostic test, *HI* haemagglutination inhibition

Socio-demographic predictors including sex, age and primary/secondary infection variables were reported in 114, 87, and 29 studies, respectively. Diabetes was the most reported co-morbidity in 10 studies, followed by hypertension in nine studies, obesity in five studies, and one each of CVD and renal disease in four studies. Other co-morbidities including asthma, pulmonary disease or sickle cell disease were not adequately reported to be analysed further. Finally, warning signs of severe dengue were reported as follows: abdominal pain (*n* = 55 studies), vomiting (*n* = 53 studies), enlarged liver size (*n* = 47 studies), pleural effusion (*n* = 25 studies), ascites (*n* = 22 studies), gum bleeding (*n* = 12 studies), epistaxis (*n* = 11 studies), lethargy (*n* = 10 studies), melena (*n* = 9 studies), increase in haematocrit with concurrent decrease in platelet count (*n* = 7 studies), gastrointestinal (GI) bleeding (*n* = 5 studies), hematemesis (*n* = 5 studies) and skin bleeding (*n* = 4 studies).

### Quality of the studies

The quality of the studies was assessed against each of the 36 safeguard items. Accordingly, the studies met all the pre-defined eligibility criteria and were from the same population and timeframe. Similarly, the attrition rate and missing values were either below 20% or non-existent in 143 studies. The procedures for data collection of covariates and outcomes were reliable and objective in 142 studies. Overall, the least deficient standards across studies were equal prognosis (88.6%), equal implementation (64.6%) and equal retention (59.4%). Temporal precedence was the most deficient standard across the studies (1.5%) (Additional file 4). This might be because most of the studies included in the review used cross-sectional designs where there is no temporal dimension.

### Quantitative analysis

#### Demographic characteristics

Children were positively associated with the development of severe disease as compared to adults (*OR* = 1.96, 95% *CI*: 1.22–3.13). Progression to severe dengue did not show a significant difference by sex (*OR* = 1.20, 95% *CI*: 0.79–1.82). Secondary dengue infection was found to be significantly associated with the development of severe disease (*OR* = 3.23, 95% *CI*: 2.28–4.57) (Table 3).

**Table 3:** Pooled estimates of odds ratio and corresponding 95% confidence intervals of patient demographic characteristics and severe dengue

Predictors	Number of studies	Pooled	Heterogeneity test	
		*OR* (95% *CI*)	*I*^2^ (%)	*P*-value
Demography				
Children	22	1.96 (1.22–3.13)	90.00	< 0.001
Female	114	1.20 (0.79–1.82)	80.3	< 0.001
Secondary infection	29	3.23 (2.28–4.57)	33.20	0.044
Co-morbidities				
Diabetes	10	2.88 (1.72–4.81)	40.9	0.085
Cardiovascular disease	4	2.27 (0.38–13.71)	70.8	0.016
Obesity	5	0.76 (0.41–1.40)	32.9	0.202
Renal disease	4	4.54 (1.55–13.31)	45.1	0.162
Hypertension	9	1.82 (0.98–3.37)	63.1	0.006
Warning signs				
↑Hct & ↓Plt*	7	5.13 (1.61–16.34)	88.1	< 0.001
Abdominal pain	55	2.00 (1.49–2.68)	70.9	< 0.001
Vomiting	53	1.80 (1.43–2.26)	62.8	< 0.001
Lethargy	10	2.73 (1.05–7.10)	85.1	< 0.001
Hepatomegaly	47	5.92 (3.29–10.65)	89.3	< 0.001
Ascitis	22	6.30 (3.75–10.60)	67.7	< 0.001
Pleural effusion	25	5.72 (3.24–10.10)	76.3	< 0.001
Gum bleeding	12	2.00 (0.86–4.66)	56.4	0.008
Epistaxis	11	1.85 (0.72–4.70)	64.4	0.002
Hemetemesis	5	12.35 (4.97–30.72)	52	0.080
Melena	9	4.05 (1.64–10.00)	78.1	< 0.001
Skin bleeding	4	1.38 (0.47–4.06)	73.5	0.010
GI bleeding	5	9.49 (2.75–32.70)	78.4	0.001

*Increase in haematocrit values with concurrent decrease in platelet count

*OR* odd ratio, *CI* confidential interval

#### Co-morbidities

Diabetes (*OR* = 2.88 95% *CI*: 1.72–4.81) and renal disease(s) (*OR* = 4.85, 95% *CI*: 1.08–21.66) were associated with severe dengue. However, other co-morbidities including hypertension (*OR* = 1.82, 95% *CI*: 0.98–3.37), CVD (*OR* = 2.27, 95% *CI*: 0.38–13.71), and obesity (*OR* = 0.76, 95% *CI*: 0.41–1.40) were not significantly associated with the severe disease (Table 3).

#### Warning signs

The definition of warning signs varied across the studies. Only one study defined abdominal pain as severe enough to warrant medical attention [17]. Persistent vomiting was defined in four ways: vomiting with signs of dehydration [18–20], ≥ 2 episodes of vomiting associated with fatigue or requiring intravenous fluid [17], at least six episodes of vomiting in 24 h [21] or vomiting during ≥ 2 consecutive days [22]. Similarly, liver enlargement was defined as > 2 cm in the midclavicular line in three studies [23–25]. No studies provided a definition of lethargy.

Progression to severe dengue was associated with a concurrent increase in haematocrit and decrease in platelet count compared to normal values (*OR* = 5.13, 95% *CI*: 1.61–16.34), abdominal pain (*OR* = 2.00, 95% *CI*: 1.49–2.68), lethargy (*OR* = 2.73, 95% *CI*: 1.05–7.09), vomiting (*OR* = 1.80, 95% *CI*: 1.43–2.26) and enlarged liver (*OR* = 5.92, 95% *CI*: 3.29–10.65) (Table 3).

Studies have used different definitions for mucosal bleeding and clinical fluid accumulation. Some studies used specific conditions like epistaxis [26, 27] or gum bleeding [28, 29] to refer to mucosal bleeding, while others have grouped them as mucosal bleeding [30, 31]. Similarly, clinical fluid accumulation was defined as ascites [24, 32] or pleural effusion or combined as clinical fluid accumulation [17, 33]. Here, we presented only specific conditions rather than the grouped variable. In terms of clinical fluid accumulation, both ascites (*OR* = 6.94, 95% *CI*: 3.75–10.60) and pleural effusion (*OR* = 5.72, 95% *CI*: 3.24–10.10) were significantly associated with severe dengue. In terms of mucosal bleeding, hematemesis (*OR* = 12.35, 95% *CI*: 4.97–30.72) was significantly associated, while gum bleeding (*OR* = 2.00, 95% *CI*: 0.86–4.66) and epistaxis (*OR* = 1.85, 95% *CI*: 0.72–4.70) were not significantly associated with severe dengue. In addition, GI bleeding (*OR* = 9.49, 95% *CI*: 2.75–32.70) and melena (*OR* = 4.05, 95% *CI*: 1.69–10.00) were also found to be positively associated with severe disease (Table 3). The forest plots are presented in additional file 5.

### Subgroup analysis

All predictors that were significantly associated in the main analysis also showed similar results in the stratified analysis using the WHO 1997 and 2009 dengue case classifications. These included age groups, secondary infection, abdominal pain, vomiting, enlarged liver size, ascites, pleural effusion, hematemesis and melena. Similar to the main analysis, sex, epistaxis and gum bleeding were not significant in the stratified analysis (Additional file 6).

In the subgroup analysis by age, only adult females were significantly associated with severe dengue (relative to adult males, *OR* = 2.12, 95% *CI*: 1.13–3.97) (Additional file 7). Due to a limited number of studies, sub-group analysis could not be performed for all predictors in the co-morbidities category, GI bleeding, and increase in haematocrit values with a concurrent decrease in platelet count.

### Sensitivity analysis

In the sensitivity analysis, when using the quality effects model, all pooled estimates were found to be consistent with the main analysis (Additional file 8).

### Publication bias

The Doi plot and LFK index revealed major asymmetries for the estimates of age group (LFK = -3.83), CVD (LFK = 2.92), renal disease(s) (LFK = -3.13), hypertension (LFK = 5.05), vomiting (LFK = 1.94), lethargy (LFK = 3.7), gum bleeding (LFK = 2.04), melena (LFK = 2.4), skin (LFK = 5.47) and GI bleeding (LFK = -2.05). A moderate to high heterogeneity of the studies might have accounted for asymmetries in these estimates (Table 3 and Additional file 9).

## Discussion

In this systematic review and meta-analysis, we found that the main predictors for severe dengue were being a child, secondary dengue infection, pre-existing co-morbidities [i.e., diabetes and renal disease(s)] and the presence of warning signs (i.e., increase in haematocrit with concurrent decrease in platelet count, abdominal pain, lethargy, vomiting, hepatomegaly, ascites, pleural effusion and melena). Most of these studies were reported from countries in the WHO-South-East Asia region.

Although there has been a shift in the incidence of DF towards older age groups [34], severe dengue continues to be an important cause of significant morbidity and mortality in children since it was first reported in the 1950s in South-East Asia [35]. Previous studies have demonstrated an increased risk of severe dengue or dengue shock syndrome in children and these conditions have been known to be common causes of hospitalization and mortality in tropical regions [36, 37]. The risk of severe dengue can be explained by greater vascular permeability in children [38]. Dengue shock results from a sudden generalized increase in microvascular permeability with less microvascular reserve to accommodate extraneous factors [38]. Therefore, clinicians should pay special attention to children in recognizing the severity of the disease and providing appropriate interventions on time. Such a strong positive association of severe disease with children also supports the delivery of future vaccines and therapeutics to pre-school and school-going children to achieve the greatest impact on disease burden.

Similar to the other reported studies [39], we found a strong association between secondary dengue infection and severe dengue. This pathogenesis might be related to antibody-dependent enhancement (ADE) in secondary infection with a different DENV serotype, where the pre-existing heterotypic antibodies bind to form immune complexes with virions without neutralizing it [40]. These virus-immune complexes facilitate virus entry and enhanced virus replication in the FcγR (fragment crystallizable gamma receptors)-bearing cells, such as monocytes, dendritic cells and macrophages. The internalized DENV particles then initiate an immune cascade which results in the evasion of innate immunity, such as the inhibition of type-1 interferon, and subsequently leads to vascular leakage and severe disease [40, 41]. Further, cytokine levels are also assumed to be elevated in secondary dengue infection [42]. Cytokines like vascular cell adhesion molecule-1 (VCAM-1) facilitate chemotaxis by mediating the adhesion of lymphocytes and cells of the innate immune system to the vascular endothelium [43]. Other cytokines such as vascular endothelial growth factor-A (VEGF-A) enhance vascular permeability and activate the coagulation system by upregulating the production of tissue factors [44, 45]. Finally, biosynthesis of other pro-inflammatory cytokines such as interleukins (IL-6, IL-7, IL-8 and IL-10) facilitates an increased synthesis of DENV RNA (ribonucleic acid) and suppresses the host mediated and adaptive immune responses [41, 46]. However, it is important to note that the severity may be affected by certain DENV serotypes; the other meta-analysis study reported severe disease in secondary infection with DENV-2, 3 and 4 [39]. To provide accurate management of dengue, clinicians should rely on tests that detect both recent and past infections.

Our study also found a significantly higher risk of severe dengue due to pre-existing co-morbidities like diabetes and renal disease. This finding supports the need for hospitalization and monitoring of dengue patients with pre-existing co-morbidities [10]. Although no clear mechanism was postulated, in diabetes, patients with suboptimal glycaemic control (HbA1c ≥ 7%) were found to be strongly associated with severe dengue than were patients with adequate glycaemic control and without other co-morbidities [47]. In advanced diabetes, micro and macro-vascular functions are impaired, which might lead to increased plasma leakage and subsequently progress to severe dengue [48, 49]. In chronic kidney disease(s), pro-inflammatory cytokines are markedly elevated, which might cause vascular injury in dengue virus infection [50]. In addition, the uraemia associated with kidney disease induces endothelium dysfunction and contributes to greater vascular damage with dengue infection [51].

Patients with warning signs have to be admitted into the hospital for close monitoring and intravenous fluid therapy administration [10]. These interventions can reduce the frequency of patients progressing to severe dengue and deaths. However, none of the studies so far have comprehensively studied all warning signs identified by the WHO. Some studies [52] used thrombocytopenia and elevated thrombocytopenia separately to assess the risk of developing severe disease. However, these parameters have to be interpreted with other concurrent laboratory results. For example, an increase in haematocrit with a concurrent decrease in platelet count is an important warning sign.

As expected, our study found all warning signs to be positively associated with severe dengue excepting cutaneous and mucosal bleeding (epistaxis and gum bleeding). Notably, gastrointestinal bleeding/melena was significantly associated with severe disease. A previous study on the clinical predictors of severe dengue also found similar findings [53]. Similar to a previously published study [52], fluid accumulation, vomiting and abdominal pain was found to be positively associated with the severe disease in this study. In addition, lethargy, abdominal pain, vomiting and hepatomegaly were strongly associated with an increase in haematocrit with a concurrent decrease in platelet count. None of the meta-analyses in the past have pooled this estimate, possibly due to a low number of studies.

The findings of this study should be interpreted in the context of some limitations. We were not able to consider the role of viremia, dengue virus serotypes, genetic, biomarker and other clinical parameters besides warning signs as predictors of severe dengue. Second, we were unable to analyse different co-morbidities such as sickle cell disease and bleeding disorders despite our broad search strategy. These disorders could affect the progression to severe disease and possible outcomes. Third, there was inconsistent reporting of heart diseases which made it difficult to assess these conditions individually as potential predictors, rather we combined different heart conditions into a single group. Fourth, many studies did not report adjusted effect sizes, and we based our pooled result on crude effect sizes. These might have overestimated the pooled effect sizes due to potential confounders. This is of particular concern with smaller studies and therefore our results need to be interpreted with caution. Fifth, limiting papers published in English might have influenced the precision of the pooled estimates. Importantly, most of the studies were from the South-East Asia region and the Pacific region, which bears more than 75% of the global burden of dengue. Sixth, we did not include biomarkers of severe dengue. Finally, we found both heterogeneity and publication bias in the included studies. These might be related to variations in study design, sample sizes, recruitment processes and exposure/outcome measurement across different studies. However, we conducted subgroup analysis and sensitivity analysis to account for these variations and tested the robustness of our results.

## Conclusions

Our meta-analysis identified children, secondary infection, diabetes and renal disease(s) as important predictors of severe dengue. We also confirmed the predictive ability of all warning signs of severe dengue identified by the WHO. All warning signs were significantly associated with severe disease excepting mucosal and cutaneous bleeding. The knowledge generated from this study will help clinicians to identify early warning signals of severe dengue leading to timely interventions of dengue cases. Future studies using novel biomarkers and point of care methods including ultrasonography will be useful in predicting the onset of severe dengue.

## Supplementary Information

**Additional file 1.** PRISMA Checklist for systematic reviews and meta-analysis

**Additional file 2.** Search strategy used in the review of literature

**Additional file 3.** Studies included in the review and meta-analysis

**Additional file 4.** Quality assessment of the studies using a MASTER Scale

**Additional file 5.** Forest plots with pooled OR of progression to severe dengue with potential predictors

**Additional file 6.** Subgroup analysis using WHO classification for demography, co-morbidities and clinical warning signs of dengue severity

**Additional file 7.** Subgroup analysis using age groups for demography and warning signs of severe dengue

**Additional file 8.** Sensitivity analysis

**Additional file 9.** Doi plots and LFK values used to assess publication bias

## Data Availability

The datasets used and/or analysed during the current study are included this published article and the additional files, all of which are also available in the public domain.

## Abbreviations

ADE: Antibody-dependent enhancement
CFR: Case fatality rate
COPD: Chronic obstructive pulmonary disease
CVD: Cardiovascular diseases
CYD-TDV: Chimeric yellow fever virus—DENV tetravalent dengue vaccine
DENV: Dengue virus
DF: Dengue fever
DHF: Dengue haemorrhagic fever
DWoWS: Dengue without warning signs
DWWS: Dengue with warning signs
DSS: Dengue shock syndrome DSS
FcγR: Fragment crystallizable gamma receptors
GI: Gastrointestinal bleeding
HCT: Haematocrit
IVhet model: Inverse variance heterogeneity model
*O**R*: Odds ratio
PRISMA: Preferred Reporting Items for Systematic Reviews and Meta-Analyses
RNA: Ribonucleic acid
SD: Severe dengue
VCAM-1: Vascular cell adhesion molecule-1
WHO: World Health Organization

## References

[1] Bhatt S, Gething PW, Brady OJ, Messina JP, Farlow AW, Moyes CL, Drake JM, Brownstein JS, Hoen AG, Sankoh O (2013). The global distribution and burden of dengue. Nature.

[2] Thomas SJ, Endy TP (2011). Vaccines for the prevention of dengue: development update. Hum Vaccin.

[3] World Health Organization: Dengue and severe dengue. https://www.who.int/news-room/fact-sheets/detail/dengue-and-severe-dengue. Accessed July 07 2021.

[4] Weaver SC, Vasilakis N (2009). Molecular evolution of dengue viruses: contributions of phylogenetics to understanding the history and epidemiology of the preeminent arboviral disease. Infect Genet Evol.

[5] World Health Organization. Dengue haemorrhagic fever: diagnosis, treatment, prevention and control. 2nd edition. Geneva 1997.

[6] World Health Organization. Revised SAGE recommendation on use of dengue vaccine. 2018. https://www.who.int/immunization/diseases/dengue/revised_SAGE_recommendations_dengue_vaccines_apr2018/en/. Accessed July 09 2021.

[7] The Lancet Infectious D: the dengue vaccine dilemma. Lancet Infect Dis. 2018;18:123. 10.1016/S1473-3099(18)30023-9 29412952

[8] Capeding MR, Tran NH, Hadinegoro SR, Ismail HI, Chotpitayasunondh T, Chua MN, Luong CQ, Rusmil K, Wirawan DN, Nallusamy R (2014). Clinical efficacy and safety of a novel tetravalent dengue vaccine in healthy children in Asia: a phase 3, randomised, observer-masked, placebo-controlled trial. Lancet.

[9] Tsheten T, Gray DJ, Clements ACA, Wangdi K (2021). Epidemiology and challenges of dengue surveillance in the WHO South-East Asia Region. Trans R Soc Trop Med Hyg.

[10] World Health Organization: Dengue guidelines for diagnosis, treatment, prevention and control: New Edition. World Health Organization: Geneva. In*.*; 2009. 23762963

[11] World Health Organization. Background paper on dengue vaccine. https://www.who.int/immunization/sage/meetings/2018/april/2_DengueBackgrPaper_SAGE_Apr2018.pdf. Accessed July 10 2021.

[12] Page MJ, McKenzie JE, Bossuyt PM, Boutron I, Hoffmann TC, Mulrow CD, Shamseer L, Tetzlaff JM, Moher D (2021). Updating guidance for reporting systematic reviews: development of the PRISMA 2020 statement. J Clin Epidemiol.

[13] Stone JC, Glass K, Clark J, Ritskes-Hoitinga M, Munn Z, Tugwell P, Doi SAR (2021). The MethodologicAl Standards for Epidemiological Research (MASTER) scale demonstrated a unified framework for bias assessment. J Clin Epidemiol.

[14] Doi SA, Barendregt JJ, Khan S, Thalib L, Williams GM (2015). Advances in the meta-analysis of heterogeneous clinical trials I: the inverse variance heterogeneity model. Contemp Clin Trials.

[15] Doi SA, Thalib L (2008). A quality-effects model for meta-analysis. Epidemiology.

[16] Furuya-Kanamori L, Barendregt JJ, Doi SAR (2018). A new improved graphical and quantitative method for detecting bias in meta-analysis. Int J Evid Based Healthc.

[17] Sreenivasan P, Geetha S, Sasikala K (2018). Development of a prognostic prediction model to determine severe dengue in children. Indian J Pediatr.

[18] Thanachartwet V, Oer-Areemitr N, Chamnanchanunt S, Sahassananda D, Jittmittraphap A, Suwannakudt P, Desakorn V, Wattanathum A (2015). Identification of clinical factors associated with severe dengue among Thai adults: a prospective study. BMC Infect Dis.

[19] Rafi A, Mousumi AN, Ahmed R, Chowdhury RH, Wadood A, Hossain G (2020). Dengue epidemic in a non-endemic zone of Bangladesh: clinical and laboratory profiles of patients. PLoS Negl Trop Dis.

[20] Aung KL, Thanachartwet V, Desakorn V, Chamnanchanunt S, Sahassananda D, Chierakul W, Pitisuttithum P (2013). Factors associated with severe clinical manifestation of dengue among adults in Thailand. Southeast Asian J Trop Med Public Health.

[21] Mercado ES, Espino FE, Perez ML, Bilar JM, Bajaro JD, Huy NT, Baello BQ, Kikuchi M, Hirayama K (2015). HLA-A*33:01 as protective allele for severe dengue in a population of Filipino children. PLoS ONE.

[22] Carrasco LR, Leo YS, Cook AR, Lee VJ, Thein TL, Go CJ, Lye DC (2014). Predictive tools for severe dengue conforming to World Health Organization 2009 criteria. PLoS Negl Trop Dis.

[23] Prasad D, Bhriguvanshi A (2020). Clinical profile, liver dysfunction and outcome of dengue infection in children: a prospective observational study. Pediatr Infect Dis J.

[24] Wakimoto MD, Camacho LAB, Gonin ML, Brasil P (2018). Clinical and laboratory factors associated with severe dengue: a case-control study of hospitalized children. J Trop Pediatr.

[25] Hoffmeister B, Suttorp N, Zoller T (2015). The revised dengue fever classification in German travelers: clinical manifestations and indicators for severe disease. Infection.

[26] Sahu AK, Aggarwal P, Ekka M, Nayer J, Bhoi S, Kumar A, Luthra K (2021). Assessing the serum chymase level as an early predictor of dengue severity. J Med Virol.

[27] Zhang H, Xie Z, Xie X, Ou Y, Zeng W, Zhou Y (2018). A novel predictor of severe dengue: the aspartate aminotransferase/platelet count ratio index (APRI). J Med Virol.

[28] Hanafusa S, Chanyasanha C, Sujirarat D, Khuankhunsathid I, Yaguchi A, Suzuki T (2008). Clinical features and differences between child and adult dengue infections in Rayong Province, Southeast Thailand. Southeast Asian J Trop Med Public Health.

[29] Falconar AK, Romero-Vivas CM (2012). Simple prognostic criteria can definitively identify patients who develop severe versus non-severe dengue disease, or have other febrile illnesses. J Clin Med Res.

[30] Sabeena S, Chandrabharani K, Ravishankar N, Arunkumar G (2018). Classification of dengue cases in southwest India based on the WHO systems—a retrospective analysis. Trans R Soc Trop Med.

[31] Duangmala T, Lumbiganon P, Kosalaraksa P (2014). Unusual clinical manifestations of dengue infection in children in a tertiary care hospital in northeast Thailand. Asian Biomed.

[32] Md Sani SS, Han WH, Bujang MA, Ding HJ, Ng KL, Amir Shariffuddin MA (2017). Evaluation of creatine kinase and liver enzymes in identification of severe dengue. BMC Infect Dis.

[33] Hegazi MA, Bakarman MA, Alahmadi TS, Butt NS, Alqahtani AM, Aljedaani BS, Almajnuni AH (2020). Risk factors and predictors of severe dengue in saudi population in Jeddah, Western Saudi Arabia: a retrospective study. Am J Trop Med Hyg.

[34] Egger JR, Coleman PG (2007). Age and clinical dengue illness. Emerg Infect Dis.

[35] Ooi E-E, Gubler DJ (2009). Dengue in Southeast Asia: epidemiological characteristics and strategic challenges in disease prevention. Cad Saude Publica.

[36] Anders KL, Nguyet NM, Chau NV, Hung NT, Thuy TT, le Lien B, Farrar J, Wills B, Hien TT, Simmons CP (2011). Epidemiological factors associated with dengue shock syndrome and mortality in hospitalized dengue patients in Ho Chi Minh City, Vietnam. Am J Trop Med Hyg.

[37] Teixeira MG, Siqueira JB, Ferreira GL, Bricks L, Joint G (2013). Epidemiological trends of dengue disease in Brazil (2000–2010): a systematic literature search and analysis. PLoS Negl Trop Dis.

[38] Gamble J, Bethell D, Day NP, Loc PP, Phu NH, Gartside IB, Farrar JF, White NJ (2000). Age-related changes in microvascular permeability: a significant factor in the susceptibility of children to shock?. Clin Sci (Lond).

[39] Soo K-M, Khalid B, Ching S-M, Chee H-Y (2016). Meta-analysis of dengue severity during infection by different dengue virus serotypes in primary and secondary infections. PLoS ONE.

[40] Katzelnick LC, Gresh L, Halloran ME, Mercado JC, Kuan G, Gordon A, Balmaseda A, Harris E (2017). Antibody-dependent enhancement of severe dengue disease in humans. Science.

[41] Narayan R, Tripathi S (2020). Intrinsic ADE: the dark side of antibody dependent enhancement during dengue infection. Front Cell Infect Microbiol.

[42] Chaturvedi UC, Agarwal R, Elbishbishi EA, Mustafa AS (2000). Cytokine cascade in dengue hemorrhagic fever: implications for pathogenesis. FEMS Immunol Med Microbiol.

[43] Murgue B, Cassar O, Deparis X (2001). Plasma concentrations of sVCAM-1 and severity of dengue infections. J Med Virol.

[44] Senger DR, Galli SJ, Dvorak AM, Perruzzi CA, Harvey VS, Dvorak HF (1983). Tumor cells secrete a vascular permeability factor that promotes accumulation of ascites fluid. Science.

[45] Mangione JN, Huy NT, Lan NT, Mbanefo EC, Ha TT, Bao LQ, Nga CT, Tuong VV, Dat TV, Thuy TT (2014). The association of cytokines with severe dengue in children. Trop Med Health.

[46] Soo KM, Khalid B, Ching SM, Tham CL, Basir R, Chee HY (2017). Meta-analysis of biomarkers for severe dengue infections. PeerJ.

[47] Lee IK, Hsieh CJ, Lee CT, Liu JW (2020). Diabetic patients suffering dengue are at risk for development of dengue shock syndrome/severe dengue: emphasizing the impacts of co-existing comorbidity(ies) and glycemic control on dengue severity. J Microbiol Immunol Infect.

[48] Tight blood pressure control and risk of macrovascular and microvascular complications in type 2 diabetes: UKPDS 38. UK Prospective Diabetes Study Group. *BMJ*. 1998;317:703–713. PMC28659 9732337

[49] Stratton IM, Adler AI, Neil HA, Matthews DR, Manley SE, Cull CA, Hadden D, Turner RC, Holman RR (2000). Association of glycaemia with macrovascular and microvascular complications of type 2 diabetes (UKPDS 35): prospective observational study. BMJ.

[50] Pecoits-Filho R, Heimbürger O, Bárány P, Suliman M, Fehrman-Ekholm I, Lindholm B, Stenvinkel P (2003). Associations between circulating inflammatory markers and residual renal function in CRF patients. Am J Kidney Dis.

[51] Aznar-Salatti J, Escolar G, Cases A, Gómez-Ortiz G, Anton P, Castillo R, Revert L, Ordinas A (1995). Uraemic medium causes endothelial cell dysfunction characterized by an alteration of the properties of its subendothelial matrix. Nephrol Dial Transplant.

[52] Sangkaew S, Ming D, Boonyasiri A, Honeyford K, Kalayanarooj S, Yacoub S, Dorigatti I, Holmes A (2021). Risk predictors of progression to severe disease during the febrile phase of dengue: a systematic review and meta-analysis. Lancet Infect Dis.

[53] Zhang H, Zhou YP, Peng HJ, Zhang XH, Zhou FY, Liu ZH, Chen XG (2014). Predictive symptoms and signs of severe dengue disease for patients with dengue fever: a meta-analysis. BioMed Res Int.

