# Supplementary material for: Clinical predictors of severe dengue: a systematic review and meta-analysis
Source: Infect Dis Poverty. 2021 Oct 9;10:123. doi: 10.1186/s40249-021-00908-2 (PMC8501593; doi:10.1186/s40249-021-00908-2)
Supplement: Supplementary file 2 — Additional file 2. Search strategy used in the review of literature [file 40249_2021_908_MOESM2_ESM.docx]

(("Severe Dengue"  OR “dengue hemorrhagic fever” OR “dengue haemorrhagic fever” OR “dengue shock syndrome” OR “dengue complication*” OR “dengue death*” OR “dengue mortalit*” OR “dengue fatalit*” OR “dengue intensive care”) AND (“undifferentiated dengue fever” OR “non-severe dengue” OR “dengue fever” OR “dengue without warning signs” OR “dengue with warning signs” OR dengue)) AND (comorbidit* OR “chronic disease*” OR "Nutritional and Metabolic Disease*" OR "Central Nervous System Disease*" OR "Respiratory Tract Disease*" OR "Digestive System Disease*" OR "Urologic Disease*" OR "Metabolic Disease*" OR "Endocrine System Disease*" OR diabetes OR hypertension OR “cardiac disorder” OR “cardiovascular disease*” OR “coronary heart disease*” OR “cerebrovascular disease*” OR “arterial disease*” OR deafness OR “hearing loss*” OR obesity OR cancer OR alcohol OR arthritis OR osteoporosis OR “sickle-cell disease” OR “sickle cell disease” OR “hemoglobin disorder*” OR “haemoglobin disorder*” OR oral OR dental OR malnutrition OR overweight OR underweight OR asthma OR allerg* OR stroke OR “heart disease*” OR embolism OR thrombosis OR anaemia OR anemia OR Demography OR "Age Factors" OR Sex OR "Ethnic Groups" OR "Social Class" OR demograph* OR gender OR age OR sex OR ethnic OR location OR region* OR background OR residen* OR nation* OR class OR income OR econom* OR “socio-economic”)
