# Supplementary material for: Clinical predictors of severe dengue: a systematic review and meta-analysis
Source: Infect Dis Poverty. 2021 Oct 9;10:123. doi: 10.1186/s40249-021-00908-2 (PMC8501593; doi:10.1186/s40249-021-00908-2)
Supplement: Supplementary file 3 — Additional file 3. Studies included in the review and meta-analysis [file 40249_2021_908_MOESM3_ESM.docx]

| Author | Country | Start-year | End-year | Study population | Study design | Classification | Reference |
| --- | --- | --- | --- | --- | --- | --- | --- |
| Tan VPK et al | Malaysia | 2015 | 2015 | Adults | Cross-sectional | 2009 | [1] |
| Burattini MN et al | Brazil | 2000 | 2014 | Mixed | Cross-sectional | 1997 | [2] |
| Kuo HJ et al | Taiwan | 2008 | 2014 | Adults | Cross-sectional | 2009 | [3] |
| Junior JJD et al | Brazil | 2002 | 2011 | Mixed | Cross-sectional | 2009 | [4] |
| Richards AL et al | Indonesia | 1993 | 1993 | Mixed | Cross-sectional | 1997 | [5] |
| Sahu AK et al | India | 2018 | 2020 | Adults | Cross-sectional | 2009 | [6] |
| Ditsuwan T et al | Thailand | 2009 | 2010 | Mixed | Cross-sectional | 1997 | [7] |
| Patra G et al | India | 2016 | 2017 | Mixed | Cross-sectional | 2009 | [8] |
| Mayetti M et al | Indonesia | 2016 | 2017 | Children | Cross-sectional | 1997 | [9] |
| Mangione JNA et al | Vietnam | 2006 | 2007 | Children | Case control | 1997 | [10] |
| Singh J et al | India | 2014 | 2014 | Mixed | Cross-sectional | 1997 | [11] |
| Yeung W et al | Singapore | 2005 | 2008 | Adults | Cohort | 2009 | [12] |
| Yacoub S et al | Vietnam | 2008 | 2008 | Adults | Cross-sectional | 2009 | [13] |
| Giraldo D et al | Brazil | 2007 | 2008 | Children | Cohort | 2009 | [14] |
| Hair GM et al | Brazil | 2007 | 2013 | Mixed | Cross-sectional | 2009 | [15] |
| Sabeena S et al | India | 2011 | 2012 | Mixed | Cross-sectional | 1997/2009 | [16] |
| Lin YP et al | China | 2014 | 2014 | Mixed | Cross-sectional | 2009 | [17] |
| Rathakrishnan A et al | Malaysia | 2010 | 2011 | Adults | Cross-sectional | 2009 | [18] |
| Shah I et al | India | 2004 | 2004 | Children | Cross-sectional | 1997 | [19] |
| Wakimato MD et al | Brazil | 2007 | 2008 | Children | Case control | 2009 | [20] |
| Kumar A et al | Barbados | 2009 | 2009 | Children | Cross-sectional | 2009 | [21] |
| Hanafusa S et al | Thailand | 2004 | 2005 | Mixed | Cross-sectional | 1997 | [22] |
| Trung DT et al | Vietnam | 2006 | 2008 | Mixed | Cross-sectional | 1997 | [23] |
| Agrawal VK et al | India | 2015 | 2017 | Mixed | Cross-sectional | 2009 | [24] |
| Adam AS et al | Indonesia | 2014 | 2016 | Children | Cross-sectional | 2009 | [25] |
| Sahana KS et al | India | 2012 | 2013 | Children | Cross-sectional | 2009 | [26] |
| Bisoyi SK et al | India | 2016 | 2016 | Adults | Cross-sectional | 2009 | [27] |
| Mishra S et al | India | 2013 | 2015 | Children | Cohort | 2009 | [28] |
| Tun MMN et al | Vietnam | 2017 | 2017 | Children | Case control | 2009 | [29] |
| Mittal H et al | India | 2010 | 2010 | Children | Cross-sectional | 1997 | [30] |
| Vaddadi K et al | India | 2014 | 2014 | Children | Cross-sectional | 2009 | [31] |
| Suwarto S et al | Indonesia | 2010 | 2016 | Adults | Cohort | 2009 | [32] |
| Gupta S et al | India | 2013 | 2015 | Children | Cross-sectional | 2009 | [33] |
| Kerdpanich P et al | Thailand | 2002 | 2018 | Mixed | Cross-sectional | 1997 | [34] |
| Dussart P et al | Cambodia | 2011 | 2012 | Children | Cross-sectional | 1997/2009 | [35] |
| Tsai CY et al | Taiwan | 2008 | 2010 | Mixed | Cross-sectional | 2009 | [36] |
| Noecker CA et al | Mexico | 2011 | 2011 | Adults | Cohort | 2009 | [37] |
| Rathakrishnan A et al | Malaysia | 2005 | 2009 | Adults | Case control | 2009 | [38] |
| Khongphatthanayothin A et al | Thailand | 2002 | 2005 | Children | Cohort | 1997 | [39] |
| Pongsumpun P et al | Thailand | 2004 | 2009 | Mixed | Cross-sectional | 1997 | [40] |
| Karyanti MR et al | Indonesia | 2009 | 2013 | Children | Cohort | 1997 | [41] |
| Rafi A et al | Bangladesh | 2019 | 2019 | Adults | Cross-sectional | 2009 | [42] |
| Narayanan M et al | India | 2001 | 2001 | Children | Cohort | 1997 | [43] |
| Boillat-Blanco N et al | Tanzania | 2013 | 2014 | Adults | Cohort | 2009 | [44] |
| Kabra SK et al | India | 1996 | 1996 | Children | Cross-sectional | 1997 | [45] |
| Alvis-Guzman N et al | Colombia | 2003 | 2010 | Mixed | Cross-sectional | 2009 | [46] |
| Elenga N et al | France | 2005 | 2013 | Children | Cohort | 2009 | [47] |
| Anita C et al | India | 2008 | 2009 | Adults | cross-sectional | 1997 | [48] |
| Nunes PCG et al | Brazil | 1990 | 2011 | Mixed | Cross-sectional | 2009 | [49] |
| Rocha BAM et al | Brazil | 2012 | 2013 | Mixed | Cohort | 2009 | [50] |
| ABUALAMAH WA et al | Saudi Arabia | 2006 | 2016 | Mixed | Case control | 2009 | [51] |
| Srinivasan P et al | India | 2015 | 2016 | Children | Case control | 2009 | [52] |
| Lee Ik et al | Taiwan | 2002 | 2015 | Adults | Cross-sectional | 2009 | [53] |
| Pongpan S | Thailand | 2007 | 2010 | Children | Cross-sectional | 1997 | [54] |
| Barros TAC et al | Brazil | 2010 | 2013 | Adults | Cross-sectional | 2009 | [55] |
| Naranjo‐Gómez JS et al | Colombia | 2014 | 2016 | Adults | Cross-sectional | 2009 | [56] |
| Kaur N et al | Malaysia | 2018 | 2018 | Mixed | Cross-sectional | 2009 | [57] |
| Koraka P et al | Indonesia | 1995 | 1996 | Children | Cross-sectional | 1997 | [58] |
| MD San SS et al | Malaysia | 2014 | 2015 | Adults | Cohort | 2009 | [59] |
| Pozo-Aguilar JO et al | Mexico | 2009 | 2009 | Mixed | Cross-sectional | 2009 | [60] |
| Vejchapipat p | Thailand | 2003 | 2004 | Children | Cross-sectional | 1997 | [61] |
| Nguyen MT et al | Vietnam | 2010 | 2013 | Children | Cohort | 2009 | [62] |
| Aung KLL et al | Thailand | 2006 | 2010 | Adults | Cross-sectional | 2009 | [63] |
| Pereira MS et al | India | Not reported | Not reported | Adults | Cross-sectional | 2009 | [64] |
| Khan MIH et al | Pakistan | 2011 | 2011 | Mixed | Cross-sectional | 1997 | [65] |
| Devignot S et al | Cambodia | 2007 | 2007 | Children | Cross-sectional | 1997 | [66] |
| Low GKK et al | Malaysia | 2015 | 2015 | Mixed | Cross-sectional | 2009 | [67] |
| Mercado ES et al | Philippines | 2008 | 2009 | Children | Case control | 1997 | [68] |
| Niriella MA et al | Sri Lanka | 2017 | 2017 | Mixed | Cross-sectional | 2009 | [69] |
| Singla M et al | India | 2012 | 2014 | Children | Cohort | 2009 | [70] |
| Maneerattanasak S et al | Thailand | 2017 | 2018 | Children | Cross-sectional | 1997 | [71] |
| Heringer M et al | Brazil | 2010 | 2012 | Mixed | Cross-sectional | 2009 | [72] |
| Shabrish S et al | India | 2016 | 2017 | Mixed | Case control | 2009 | [73] |
| Juffrie M et al | Indonesia | 1995 | 1996 | Children | Cross-sectional | 1997 | [74] |
| Vicente CR et al | Brazil | 2007 | 2013 | Mixed | Cross-sectional | 2009 | [75] |
| Yolanda N et al | Indonesia | 2016 | 2016 | Children | Cross-sectional | 2009 | [76] |
| Arayasongsak U et al | Thailand | 2000 | 2004 | Children | Case control | 1997 | [77] |
| Shaheedha SM et al | India | 2017 | 2018 | Children | Cross-sectional | 2009 | [78] |
| van de Weg CA et al | Indonesia | 2001 | 2003 | Children | Case control | 1997 | [79] |
| Biswas HH et al | Nicaragua | 2005 | 2013 | Children | Cohort | 2009 | [80] |
| van de Weg CA | Brazil | 2010 | 2010 | Mixed | Cross-sectional | 2009 | [81] |
| Temprasertrudee S et al | Thailand | 2013 | 2015 | Adults | Cohort | 2009 | [82] |
| Humayoun MA et al | Pakistan | 2008 | 2008 | Mixed | Cross-sectional | 1997 | [83] |
| Vasey B et al | Venezuela | 2001 | 2005 | Mixed | Cohort | 2009 | [84] |
| Buntubatu S et al | Indonesia | 2015 | 2016 | Children | Cohort | 1997 | [85] |
| Zhang H et al | China | 2014 | 2014 | Adults | Cross-sectional | 2009 | [86] |
| Davidson TD et al | Jamaica | 2012 | 2012 | Children | Cross-sectional | 2009 | [87] |
| Phuong NTN et al | Vietnam | 2011 | 2013 | Mixed | Cohort | 2009 | [88] |
| Phakhounthong K et al | Cambodia | 2009 | 2010 | Children | Cross-sectional | 2009 | [89] |
| Potts JA et al | Thailand | 1994 | 2007 | Children | Cohort | 1997 | [90] |
| Low GKK et al | Malaysia | 2016 | 2017 | Adults | Cohort | 2009 | [91] |
| Carrasco LR et al | Singapore | 2006 | 2008 | Adults | Cohort | 2009 | [92] |
| Jain S et al | India | 2015 | 2015 | Adults | Cross-sectional | 1997 | [93] |
| Shams N et al | Pakistan | 2015 | 2015 | Adults | Cross-sectional | 1997 | [94] |
| Torrentes-Carvalho A et al | Brazil | 2010 | 2011 | Not reported | Cohort | 2009 | [95] |
| Dang TN et al | Thailand | 1999 | 2004 | Children | Cross-sectional | 1997 | [96] |
| Vazhayil PP et al | India | 2015 | 2016 | Children | Cross-sectional | 2009 | [97] |
| Hoffmeister B et al | Germany | 1996 | 2010 | Mixed | Cross-sectional | 2009 | [98] |
| Wichmann O et al | Thailand | 2001 | 2001 | Mixed | Cross-sectional | 1997 | [99] |
| Hegazi MA et al | Saudi Arabia | 2010 | 2016 | Mixed | Cross-sectional | 2009 | [100] |
| Perdomo-Celis F et al | Colombia | 2013 | 2015 | Children | Cross-sectional | 2009 | [101] |
| Chaiyaratana W et al | Thailand | 2002 | 2005 | Children | Cohort | 1997 | [102] |
| Soundravally R et al | India | 2012 | 2013 | Mixed | Case control | 2009 | [103] |
| Liao B et al | China | 2013 | 2013 | Adults | Cohort | 2009 | [104] |
| Trairatvorakul P et al | Thailand | 2003 | 2004 | Children | Case control | 1997 | [105] |
| Balmaseda A et al | Nicaragua | 1999 | 2001 | Mixed | Cross-sectional | 1997 | [106] |
| Soundravally R et al | India | 2003 | 2003 | Adults | Cross-sectional | 1997 | [107] |
| Falconar AKI et al | Colombia | 2007 | 2007 | Mixed | Cohort | 2009 | [108] |
| Zhao L et al | China | 2013 | 2013 | Adults | Case control | 2009 | [109] |
| Wangdi K et al | Timor-Leste | 2005 | 2013 | Mixed | Cross-sectional | 1997 | [110] |
| Rathi M et al | India | 2013 | 2013 | Children | Cross-sectional | 2009 | [111] |
| Cherupanakkal C et al | India | 2012 | 2014 | Mixed | Cohort | 2009 | [112] |
| Bandyopadhyay D et al | India | 2014 | 2015 | Adults | Cross-sectional | 1997 | [113] |
| Popper SJ et al | Nicaragua | 2005 | 2007 | Children | Cross-sectional | 1997 | [114] |
| Chaudhary R et al | India | 2003 | 2003 | Mixed | Cross-sectional | 1997 | [115] |
| Duangmala T et al | Thailand | 2007 | 2011 | Children | Cross-sectional | 1997 | [116] |
| Park S et al | Thailand | 1994 | 2007 | Children | Cohort | 1997 | [117] |
| Srivastava G et al | India | 2016 | 2016 | Children | Cross-sectional | 2009 | [118] |
| Saqib MA | Pakistan | 2011 | 2011 | Mixed | Cross-sectional | 1997 | [119] |
| Mohamed NA et al | Yemen | 2009 | 2009 | Mixed | Cross-sectional | 1997 | [120] |
| Ahmed FU et al | Bangladesh | 2000 | 2000 | Children | Cohort | 1997 | [121] |
| Lam pk et al | Vietnam | 2001 | 2009 | Children | Cohort | 1997 | [122] |
| Wong jg et al | Singapore | 2010 | 2012 | Adults | Cohort | 1997 | [123] |
| Hoang lt et al | Vietnam | 2006 | 2007 | Mixed | Case control | 1997 | [124] |
| Andries AC et al | Cambodia | 2013 | 2013 | Children | Case control | 2009 | [125] |
| Furuta T et al | Vietnam | 2002 | 2005 | Children | case control | 1997 | [126] |
| Gopal SSS | India | 2013 | 2014 | Adults | Case control | 2009 | [127] |
| Mairuhi ATA et al | Indonesia | 2001 | 2003 | Children | Cohort | 1997 | [128] |
| Vuong NL et al | Vietnam | 2011 | 2013 | Mixed | Cohort | 2009 | [129] |
| Pothapregada S et al | India | 2012 | 2014 | Children | Cross-sectional | 1997 | [130] |
| Thanachartwet V et al | Thailand | 2013 | 2015 | Adults | Cohort | 1997 | [131] |
| May WL et al | Myanmar | 2015 | 2016 | Children | Cross-sectional | 2009 | [132] |
| Machain-Williams C et al | Mexico | 2013 | 2013 | Adults | Cross-sectional | 2009 | [133] |
| de Kruif MD et al | Indonesia | 2002 | 2003 | Children | Cohort | 1997 | [134] |
| Zhao H et al | China | 2013 | 2013 | Mixed | Cross-sectional | 2009 | [135] |
| Pillai AB et al | India | 2012 | 2014 | Mixed | Cohort | 2009 | [136] |
| Marin-Palma D et al | Sri Lanka | 2015 | 2017 | Adults | Case control | 2009 | [137] |
| Cherupanakkal C et al | India | 2014 | 2015 | Mixed | Case control | 2009 | [138] |
| Utama IMGD et al | Indonesia | 2016 | 2017 | Children | Cross-sectional | 1997 | [139] |
| Gibson G et al | Brazil | 2007 | 2008 | Children | Case control | 1997 | [140] |
| Prasad D et al | India | 2014 | 2015 | Children | Cross-sectional | 2009 | [141] |
| Uddin MN et al | Bangladesh | 2008 | 2010 | Adults | Cross-sectional | 1997 | [142] |
| Thanachartwet V et al | Thailand | 2012 | 2014 | Adults | Cross-sectional | 2009 | [143] |

**References**

1. Tan VPK, Ngim CF, Lee EZ, Ramadas A, Pong LY, Ng JI, Hassan SS, Ng XY, Dhanoa A: The association between obesity and dengue virus (DENV) infection in hospitalised patients. *PloS one.* 2018,13:e0200698.

2. Burattini MN, Lopez LF, Coutinho FA, Siqueira JB, Jr., Homsani S, Sarti E, Massad E: Age and regional differences in clinical presentation and risk of hospitalization for dengue in Brazil, 2000-2014. *Clinics (Sao Paulo).* 2016;71:455–463.

3. Kuo HJ, Lee IK, Liu JW: Analyses of clinical and laboratory characteristics of dengue adults at their hospital presentations based on the World Health Organization clinical-phase framework: Emphasizing risk of severe dengue in the elderly. *J Microbiol Immunol Infect.* 2018;51:740–748.

4. Dias Junior JdJ, Freitas Carvalho Branco MdR, de Sousa Queiroz RC, dos Santos AM, Borges Moreira EP, da Silva MdS: Analysis of dengue cases according to clinical severity, Sao Luis, Maranhao, Brazil. *Revista Do Instituto De Medicina Tropical De Sao Paulo.* 2017;59:e71.

5. Richards AL, Bagus R, Baso SM, Follows GA, Tan R, Graham RR, Sandjaja B, Corwin AL, Punjabi N: The first reported outbreak of dengue hemorrhagic fever in Irian Jaya, Indonesia. *Am J Trop Med Hyg.* 1997;57:49–55.

6. Sahu AK, Aggarwal P, Ekka M, Nayer J, Bhoi S, Kumar A, Luthra K: Assessing the serum chymase level as an early predictor of dengue severity. *J Med Virol.* 2020;93:3330–3337.

7. Ditsuwan T, Liabsuetrakul T, Chongsuvivatwong V, Thammapalo S, McNeil E: Assessing the Spreading Patterns of Dengue Infection and Chikungunya Fever Outbreaks in Lower Southern Thailand Using a Geographic Information System. *Ann Epidemiol.* 2011;21:253–261.

8. Patra G, Mallik S, Saha B, Mukhopadhyay S: Assessment of chemokine and cytokine signatures in patients with dengue infection: A hospital-based study in Kolkata, India. *Acta Tropica.* 2019;190:73–79.

9. Mayetti M, Jamsari A, Darwin E, Somasetia DH: Association of angiopoietin-2 level and vascular endothelial growth factor level with dengue infection severity in children. *Drug Invention Today.* 2019;12:679–683.

10. Mangione JN, Huy NT, Lan NT, Mbanefo EC, Ha TT, Bao LQ, Nga CT, Tuong VV, Dat TV, Thuy TT *et al*: The association of cytokines with severe dengue in children. *Trop Med Health.* 2014;42:137–144.

11. Singh J, Dinkar A, Atam V, Himanshu D, Gupta KK, Usman K, Misra R: Awareness and Outcome of Changing Trends in Clinical Profile of Dengue Fever: A Retrospective Analysis of Dengue Epidemic from January to December 2014 at a Tertiary Care Hospital. *J Assoc Physicians India.* 2017;65:42–46.

12. Yeung W, Lye DCB, Thein TL, Chen Y, Leo YS: Blood pressure trend in hospitalized adult dengue patients. *PloS one.* 2020;15:e0235166.

13. Yacoub S, Griffiths A, Chau TT, Simmons CP, Wills B, Hien TT, Henein M, Farrar J: Cardiac function in Vietnamese patients with different dengue severity grades. *Crit Care Med.* 2012;40:477–483.

14. Giraldo D, Sant'Anna C, Périssé AR, March Mde F, Souza AP, Mendes A, Bonfim M, Hofer CB: Characteristics of children hospitalized with dengue fever in an outbreak in Rio de Janeiro, Brazil. *Trans R Soc Trop Med Hyg.* 2011;105:601–603.

15. Macedo Hair G, Fonseca Nobre F, Brasil P: Characterization of clinical patterns of dengue patients using an unsupervised machine learning approach. *BMC Infect Dis.* 2019;19:649.

16. Sabeena S, Chandrabharani K, Ravishankar N, Arunkumar G: Classification of dengue cases in southwest India based on the WHO systems—A retrospective analysis. *Tran R Soc Trop Med Hyg.* 2018;112:479–485.

17. Lin YP, Luo Y, Chen Y, Lamers MM, Zhou Q, Yang XH, Sanyal S, Mok CK, Liu ZM: Clinical and epidemiological features of the 2014 large-scale dengue outbreak in Guangzhou city, China. *BMC Infect Dis.* 2016;16:102.

18. Rathakrishnan A, Klekamp B, Wang SM, Komarasamy TV, Natkunam SK, Sathar J, Azizan A, Sanchez-Anguiano A, Manikam R, Sekaran SD: Clinical and immunological markers of dengue progression in a study cohort from a hyperendemic area in Malaysia. *PloS one.* 2014;9:e92021.

19. Shah I, Katira B: Clinical and Laboratory abnormalities due to dengue hospitalized children in Mumbai in 2004. *Dengue Bulletin.* 2005;29:90–96.

20. Wakimoto MD, Camacho LAB, Gonin ML, Brasil P: Clinical and Laboratory Factors Associated with Severe Dengue: A Case-Control Study of Hospitalized Children. *J Trop Pediatr.* 2018;64:373–381.

21. Kumar A, Gittens-St Hilair M, Jason V, Ugwuagu C, Krishnamurthy K: The clinical characteristics and outcome of children hospitalized with dengue in Barbados, an English Caribbean country. *J Infect Dev Ctries.* 2015;9:394–401.

22. Hanafusa S, Chanyasanha C, Sujirarat D, Khuankhunsathid I, Yaguchi A, Suzuki T: Clinical features and differences between child and adult dengue infections in Rayong Province, Southeast Thailand. *Southeast Asian J Trop Med Public Health.* 2008;39:252–259.

23. Trung DT, Thao le TT, Dung NM, Ngoc TV, Hien TT, Chau NV, Wolbers M, Tam DT, Farrar J, Simmons C *et al*: Clinical features of dengue in a large Vietnamese cohort: intrinsically lower platelet counts and greater risk for bleeding in adults than children. *PLoS Negl Ttrop Dis.* 2012;6:e1679.

24. Agrawal VK, Prusty BSK, Reddy CS, Mohan Reddy GK, Agrawal RK, Sekher Srinivasarao Bandaru VC: Clinical profile and predictors of Severe Dengue disease: A study from South India. *Caspian J Intern Med.* 2018;9:334–340.

25. Adam AS, Pasaribu S, Wijaya H, Pasaribu AP, Baird K, DeJong M, Widodo D, Manosuthi W, Wijaya L, Eyanoer PC *et al*: Clinical profile and warning sign finding in children with severe dengue and non-severe dengue*. Earth Environ Sci*. 2018;125:012038.

26. Sahana KS, Sujatha R: Clinical profile of dengue among children according to revised WHO classification: analysis of a 2012 outbreak from Southern India. *Indian J Pediatr.* 2015;82:109–113.

27. Bisoyi SK, Behera TR, Patnaik N, Pradhan A: Clinical profile of dengue fever at SCB Medical College and Hospital, Cuttack, Odisha. *J Commun Dis.* 2018;50:1–6.

28. Mishra S, Ramanathan R, Agarwalla SK: Clinical Profile of Dengue Fever in Children: A Study from Southern Odisha, India. *Scientifica (Cairo).* 2016;2016:6391594.

29. Ngwe Tun MM, Nguyen TTT, Ando T, Dumre SP, Soe AM, Buerano CC, Nguyen MT, Le NTN, Pham VQ, Nguyen TH *et al*: Clinical, Virological, and Cytokine Profiles of Children Infected with Dengue Virus during the Outbreak in Southern Vietnam in 2017. *Am J Trop Med Hyg.* 2020;102:1217–1225.

30. Mittal H, Faridi MM, Arora SK, Patil R: Clinicohematological profile and platelet trends in children with dengue during 2010 epidemic in north India. *Indian J Pediatr.* 2012;79:467–471.

31. Vaddadi K, Gandikota C, Jain PK, Prasad VSV, Venkataramana M: Co-circulation and co-infections of all dengue virus serotypes in Hyderabad, India 2014. *Epidemiol Infect.* 2017;145:2563–2574.

32. Suwarto S, Ulhaq S, Widjaja B: Combination of three laboratory data as predictor of severe dengue in adults : a retrospective cohort study. *Universa Medicina.* 2017;36:19–24.

33. Gupta S, Mall P, Alam A: Combined score based on arterial lactate, aspartate transaminase and prolonged capillary refill time is a useful diagnostic criterion for identifying severe dengue. *Trans R Soc Trop Med Hyg.* 2020;114:838–846.

34. Kerdpanich P, Kongkiatngam S, Buddhari D, Simasathien S, Klungthong C, Rodpradit P, Thaisomboonsuk B, Wongstitwilairoong T, Hunsawong T, Anderson KB *et al*: Comparative Analyses of Historical Trends in Confirmed Dengue Illnesses Detected at Public Hospitals in Bangkok and Northern Thailand, 2002-2018. *Am J Trop Med Hyg.* 2020;104:1058–66.

35. Dussart P, Duong V, Bleakley K, Fortas C, Lorn Try P, Kim KS, Choeung R, In S, Andries AC, Cantaert T *et al*: Comparison of dengue case classification schemes and evaluation of biological changes in different dengue clinical patterns in a longitudinal follow-up of hospitalized children in Cambodia. *PLoS Negl Trop Dis.* 2020;14:e0008603.

36. Tsai CY, Lee IK, Lee CH, Yang KD, Liu JW: Comparisons of dengue illness classified based on the 1997 and 2009 World Health Organization dengue classification schemes. *J Microbiol Immunol Infect.* 2013;46:271–281.

37. Noecker CA, Amaya-Larios IY, Galeana-Hernández M, Ramos-Castañeda J, Martínez-Vega RA: Contrasting associations of polymorphisms in FcγRIIa and DC-SIGN with the clinical presentation of dengue infection in a Mexican population. *Acta Trop.* 2014;138:15–22.

38. Rathakrishnan A, Wang SM, Hu Y, Khan AM, Ponnampalavanar S, Lum LC, Manikam R, Sekaran SD: Cytokine expression profile of dengue patients at different phases of illness. *PloS one.* 2012;7:e52215.

39. Khongphatthanayothin A, Lertsapcharoen P, Supachokchaiwattana P, La-Orkhun V, Khumtonvong A, Boonlarptaveechoke C, Pancharoen C: Myocardial depression in dengue hemorrhagic fever: prevalence and clinical description. *Pediatr Crit Care Med.* 2007;8:524–529.

40. Pongsumpun P, Tiensuwan M: Application of log-linear models to dengue virus infection patients in thailand. *Model Assisted Statistics and Applications.* 2013;8:275–287.

41. Karyanti MR, Uiterwaal C, Hadinegoro SR, Jansen MAC, Heesterbeek J, Hoes AW, Bruijning-Verhagen P: Clinical Course and Management of Dengue in Children Admitted to Hospital: A 5 Years Prospective Cohort Study in Jakarta, Indonesia. *Pediatr Infect Dis J.* 2019;38:e314–e319.

42. Rafi A, Mousumi AN, Ahmed R, Chowdhury RH, Wadood A, Hossain G: Dengue epidemic in a non-endemic zone of Bangladesh: Clinical and laboratory profiles of patients. *PLoS Negl Trop Dis.* 2020;14:e0008567.

43. Narayanan M, Aravind MA, Thilothammal N, Prema R, Sargunam CS, Ramamurty N: Dengue fever epidemic in Chennai--a study of clinical profile and outcome. *Indian pediatrics.* 2002;39:1027–1033.

44. Boillat-Blanco N, Klaassen B, Mbarack Z, Samaka J, Mlaganile T, Masimba J, Franco Narvaez L, Mamin A, Genton B, Kaiser L *et al*: Dengue fever in Dar es Salaam, Tanzania: clinical features and outcome in populations of black and non-black racial category. *BMC Infect Dis.* 2018;18:644.

45. Kabra SK, Jain Y, Pandey RM, Madhulika, Singhal T, Tripathi P, Broor S, Seth P, Seth V: Dengue haemorrhagic fever in children in the 1996 Delhi epidemic. *Trans R Soc Trop Med Hyg.* 1999;93:294–298.

46. Alvis-Guzman N, Rodriguez-Barreto H, Mattar-Velilla S: Dengue in an area of the Colombian Caribbean, 2003-2010. *Colombia Medica.* 2015;46:3–7.

47. Elenga N, Celicourt D, Muanza B, Elana G, Hocquelet S, Tarer V, Maillard F, Sibille G, Divialle Doumdo L, Petras M *et al*: Dengue in hospitalized children with sickle cell disease: A retrospective cohort study in the French departments of America. *J Infect Public Health.* 2020;13:186–192.

48. Chakravarti A, Suresh K, Neha, Shweta, Malik S: Dengue outbreak in Delhi in 2009: study of laboratory and clinical parameters. *J Commun Dis.* 2012;44:163–168.

49. Nunes PC, Sampaio SA, da Costa NR, de Mendonça MC, Lima Mda R, Araujo SE, dos Santos FB, Simões JB, Gonçalves Bde S, Nogueira RM *et al*: Dengue severity associated with age and a new lineage of dengue virus-type 2 during an outbreak in Rio De Janeiro, Brazil. *J Med Virol.* 2016;88:1130–1136.

50. Rocha BAM, Guilarde AO, Argolo A, Tassara MP, da Silveira LA, Junqueira IC, Turchi MD, Féres VCR, Martelli CMT: Dengue-specific serotype related to clinical severity during the 2012/2013 epidemic in centre of Brazil. *Infect Dis Poverty.* 2017; 6:116.

51. Abualamah WA, Banni HS, Almasmoum HA, Allohibi YA, Samarin HM, Bafail MA: Determining Risk Factors for Dengue Fever Severity in Jeddah City, a Case-Control Study (2017). *Pol J Microbiol.* 2020;69:331–337.

52. Sreenivasan P, S G, K S: Development of a Prognostic Prediction Model to Determine Severe Dengue in Children. *Indian J Pediatr.* 2018;85:433–439.

53. Lee IK, Liu JW, Chen YH, Chen YC, Tsai CY, Huang SY, Lin CY, Huang CH: Development of a Simple Clinical Risk Score for Early Prediction of Severe Dengue in Adult Patients. *PloS one.* 2016;11:e0154772.

54. Pongpan S, Wisitwong A, Tawichasri C, Patumanond J, Namwongprom S: Development of dengue infection severity score. *ISRN Pediatr.* 2013;2013:845876.

55. Barros TADC, Batista DDO, Torrentes de Carvalho A, Costa Faria NRD, Barreto-Vieira DF, Jácome FC, Barth OM, Nogueira RMR, Neves PCDC, Matos DCDS *et al*: Different aspects of platelet evaluation in dengue: Measurement of circulating mediators, ability to interact with the virus, the degree of activation and quantification of intraplatelet protein content. *Virus Research.* 2019;260:163–172.

56. Naranjo-Gomez JS, Andres Castillo J, Rojas M, Restrepo BN, Diaz FJ, Velilla PA, Castano D: Different phenotypes of non-classical monocytes associated with systemic inflammation, endothelial alteration and hepatic compromise in patients with dengue. *Immunology.* 2019;156:147–163.

57. Kaur N, Rahim S, Jaimin JJ, Dony JJF, Khoon KT, Ahmed K: The east coast districts are the possible epicenter of severe dengue in Sabah. *J Physiol Anthropol.* 2020;39:19.

58. Koraka P, Murgue B, Deparis X, Setiati TE, Suharti C, van Gorp ECM, Hack CE, Osterhaus A, Groen J: Elevated levels of total and dengue virus-specific immunoglobulin E in patients with varying disease severity. *J Med Virol.* 2003;70:91–98.

59. Md Sani SS, Han WH, Bujang MA, Ding HJ, Ng KL, Amir Shariffuddin MA: Evaluation of creatine kinase and liver enzymes in identification of severe dengue. *BMC Infect Dis.* 2017;17:505.

60. Pozo-Aguilar JO, Monroy-Martínez V, Díaz D, Barrios-Palacios J, Ramos C, Ulloa-García A, García-Pillado J, Ruiz-Ordaz BH: Evaluation of host and viral factors associated with severe dengue based on the 2009 WHO classification. *Parasit Vectors.* 2014;7:590.

61. Vejchapipat P, Theamboonlers A, Chongsrisawat V, Poovorawan Y: An evidence of intestinal mucosal injury in dengue infection. *Southeast Asian J Trop Med Public Health.* 2006;37:79–82.

62. Nguyen MT, Ho TN, Nguyen VV, Nguyen TH, Ha MT, Ta VT, Nguyen LD, Phan L, Han KQ, Duong TH *et al*: An Evidence-Based Algorithm for Early Prognosis of Severe Dengue in the Outpatient Setting. *Clin Infect Dis.* 2017;64:656–663.

63. Aung KL, Thanachartwet V, Desakorn V, Chamnanchanunt S, Sahassananda D, Chierakul W, Pitisuttithum P: Factors associated with severe clinical manifestation of dengue among adults in Thailand. *Southeast Asian J Trop Med Public Health.* 2013;44:602–612.

64. Pereira MS, Kudru CU, Nair S, Thunga G, Kunhikatta V, Guddattu V: Factors associated with severity of illness in patients with dengue fever in a tertiary care hospital in southern India. *Asian J Pharm Clin Res.* 2018;11:272–276.

65. Khan MIH, Anwar E, Agha A, Hassanien NSM, Ullah E, Syed IA, Raja A: Factors predicting severe dengue in patients with dengue fever. *Mediterr J Hematol Infect Dis.* 2013;5:e2013014.

66. Devignot S, Sapet C, Duong V, Bergon A, Rihet P, Ong S, Lorn PT, Chroeung N, Ngeav S, Tolou HJ *et al*: Genome-wide expression profiling deciphers host responses altered during dengue shock syndrome and reveals the role of innate immunity in severe dengue. *PloS one.* 2010;5:e11671.

67. Low GKK, Papapreponis P, Isa RM, Gan SC, Chee HY, Te KK, Hatta NM: Geographical distribution and spatio-temporal patterns of hospitalization due to dengue infection at a leading specialist hospital in Malaysia. *Geospat Health.* 2018;13:642.

68. Mercado ES, Espino FE, Perez ML, Bilar JM, Bajaro JD, Huy NT, Baello BQ, Kikuchi M, Hirayama K: HLA-A*33:01 as protective allele for severe dengue in a population of Filipino children. *PloS one.* 2015;10:e0115619.

69. Niriella MA, Liyanage IK, Udeshika A, Liyanapathirana KV, A PDS, H JdS: Identification of dengue patients with high risk of severe disease, using early clinical and laboratory features, in a resource-limited setting. *Arch Virol.* 2020;165:2029–2035.

70. Singla M, Kar M, Sethi T, Kabra SK, Lodha R, Chandele A, Medigeshi GR: Immune Response to Dengue Virus Infection in Pediatric Patients in New Delhi, India-Association of Viremia, Inflammatory Mediators and Monocytes with Disease Severity. *PLoS Negl Trop Dis.* 2016;10: e0004497.

71. Maneerattanasak S, Suwanbamrung C: Impact of Nutritional Status on the Severity of Dengue Infection Among Pediatric Patients in Southern Thailand. *Pediatr Infect Dis J.* 2020; 39:e410–e416.

72. Heringer M, Nogueira RMR, de Filippis AMB, Lima MRQ, Faria NRC, Nunes PCG, Nogueira FB, dos Santos FB: Impact of the emergence and re-emergence of different dengue viruses' serotypes in Rio de Janeiro, Brazil, 2010 to 2012. *Tran R Soc Trop Med Hyg.* 2015; 109:268–274.

73. Shabrish S, Karnik N, Gupta V, Bhate P, Madkaikar M: Impaired NK cell activation during acute dengue virus infection: A contributing factor to disease severity. *Heliyon.* 2020;6:e04320.

74. Juffrie M, Vo Meer GM, Hack CE, Haasnoot K, Sutaryo VAJP, Thijs LG: Inflammatory mediators in dengue virus infection in children: Interleukin-6 and its relation to C-reactive protein and secretory phospholipase A2. *Am J Trop Med Hyg.* 2001;65:70–75.

75. Vicente CR, Cerutti Junior C, Froeschl G, Romano CM, Cabidelle ASA, Herbinger KH: Influence of demographics on clinical outcome of dengue: a cross-sectional study of 6703 confirmed cases in Vitoria, Espirito Santo State, Brazil. *Epidemiol Infect.* 2017;145:46–53.

76. Yolanda N, Alfan H: Initial clinical and laboratory profiles to predict pediatric dengue infection severity. *Paediatrica Indonesiana.* 2017;57:303–309.

77. Arayasongsak U, Naka I, Ohashi J, Patarapotikul J, Nuchnoi P, Kalambaheti T, Sa-Ngasang A, Chanama S, Chaorattanakawee S: Interferon lambda 1 is associated with dengue severity in Thailand. *Int J Infect Dis.* 2020;93:121–125.

78. Shaheedha SM, Vijaya Vara Prasad M: Investigation for assessing dengue in children with their clinical presentations. *Int J Res Pharm Sci.* 2019;10:150–154.

79. van de Weg CAM, Koraka P, van Gorp ECM, Mairuhu ATA, Supriatna M, Soemantri A, van de Vijver DAMC, Osterhaus ADME, Martina BEE: Lipopolysaccharide levels are elevated in dengue virus infected patients and correlate with disease severity. *J Clin Virol.* 2012;53:38–42.

80. Biswas HH, Gordon A, Nuñez A, Perez MA, Balmaseda A, Harris E: Lower Low-Density Lipoprotein Cholesterol Levels Are Associated with Severe Dengue Outcome. *PLoS Negl Trop Dis.* 2015;9:e0003904.

81. van de Weg CA, Pannuti CS, de Araújo ES, van den Ham HJ, Andeweg AC, Boas LS, Felix AC, Carvalho KI, de Matos AM, Levi JE *et al*: Microbial translocation is associated with extensive immune activation in dengue virus infected patients with severe disease. *PLoS Negl Trop Dis.* 2013;7:e2236.

82. Temprasertrudee S, Thanachartwet V, Desakorn V, Keatkla J, Chantratita W, Kiertiburanakul S: A Multicenter Study of Clinical Presentations and Predictive Factors for Severe Manifestation of Dengue in Adults. *Jpn J Infect Dis.* 2018;71:239–243.

83. Humayoun MA, Waseem T, Jawa AA, Hashmi MS, Akram J: Multiple dengue serotypes and high frequency of dengue hemorrhagic fever at two tertiary care hospitals in Lahore during the 2008 dengue virus outbreak in Punjab, Pakistan. *IntJ Infect Dis.* 2010;14:e54–e59.

84. Vasey B, Shankar AH, Herrera BB, Becerra A, Xhaja K, Echenagucia M, Machado SR, Caicedo D, Miller J, Amedeo P *et al*: Multivariate time-series analysis of biomarkers from a dengue cohort offers new approaches for diagnosis and prognosis. *PLoS Negl Trop Dis.* 2020;14:e0008199.

85. Buntubatu S, Prawirohartono EP, Arguni E: Myocarditis Prevalence in Paediatric Dengue Infection: A Prospective Study in Tertiary Hospital in Yogyakarta, Indonesia. *J Trop Pediatr.* 2019;65:603–608.

86. Zhang H, Xie Z, Xie X, Ou Y, Zeng W, Zhou Y: A novel predictor of severe dengue: The aspartate aminotransferase/platelet count ratio index (APRI). *J Med Virol.* 2018;90:803–809.

87. Davidson TD, Vickers I, Christie CDC: Outcome of Dengue in Hospitalized Jamaican Children. *West Indian Med J.* 2016;65:442–449.

88. Phuong NTN, Manh DH, Dumre SP, Mizukami S, Weiss LN, Van Thuong N, Ha TTN, Phuc LH, Van An T, Tieu TM *et al*: Plasma cell-free DNA: a potential biomarker for early prediction of severe dengue. *Ann Clin Microbiol Antimicrob.* 2019;18:10.

89. Phakhounthong K, Chaovalit P, Jittamala P, Blacksell SD, Carter MJ, Turner P, Chheng K, Sona S, Kumar V, Day NPJ *et al*: Predicting the severity of dengue fever in children on admission based on clinical features and laboratory indicators: application of classification tree analysis. *BMC Pediatr.* 2018;18:109.

90. Potts JA, Gibbons RV, Rothman AL, Srikiatkhachorn A, Thomas SJ, Supradish PO, Lemon SC, Libraty DH, Green S, Kalayanarooj S: Prediction of dengue disease severity among pediatric Thai patients using early clinical laboratory indicators. *PLoS Negl Trop Dis.* 2010;4:e769.

91. Low GKK, Gan SC, Zainal N, Naidu KD, Amin-Nordin S, Khoo CS, Ridzuan MI, Said RM, Mansor MM, Zailani MH *et al*: The predictive and diagnostic accuracy of vascular endothelial growth factor and pentraxin-3 in severe dengue. *Pathog Glob Health.* 2018;112:334–341.

92. Carrasco LR, Leo YS, Cook AR, Lee VJ, Thein TL, Go CJ, Lye DC: Predictive tools for severe dengue conforming to World Health Organization 2009 criteria. *PLoS Negl Trop Dis.* 2014;8:e2972.

93. Jain S, Mittal A, Sharma SK, Upadhyay AD, Pandey RM, Sinha S, Soneja M, Biswas A, Jadon RS, Kakade MB *et al*: Predictors of Dengue-Related Mortality and Disease Severity in a Tertiary Care Center in North India. *Open Forum Infect Dis.* 2017;4:ofx056.

94. Shams N, Amjad S, Yousaf N, Ahmed W, Seetlani NK, Qaisar N, Samina: Predictors of Severity of Dengue Fever in Tertiary Care Hospitals. *J Liaquat Univ Med Health Sci.* 2016;15:168–173.

95. Torrentes-Carvalho A, Marinho CF, De Oliveira-Pinto LM, De Oliveira DB, Damasco PV, Cunha RV, De Souza LJ, De Azeredo EL, Kubelka CF: Regulation of T lymphocyte apoptotic markers is associated to cell activation during the acute phase of dengue. *Immunobiology.* 2014;219:329–340.

96. Dang TN, Naka I, Sa-Ngasang A, Anantapreecha S, Chanama S, Wichukchinda N, Sawanpanyalert P, Patarapotikul J, Tsuchiya N, Ohashi J: A replication study confirms the association of GWAS-identified SNPs at MICB and PLCE1 in Thai patients with dengue shock syndrome. *BMC Med Genet.* 2014;15:58.

97. Vazhayil PP, Stephen ST, Kumar V: A Retrospective Observational Study of Dengue Fever in a Tertiary Care Center in Kerala. *Int J Sci Study.* 2017;5:30–34.

98. Hoffmeister B, Suttorp N, Zoller T: The revised dengue fever classification in German travelers: clinical manifestations and indicators for severe disease. *Infection.* 2015;43:21–28.

99. Wichmann O, Hongsiriwon S, Bowonwatanuwong C, Chotivanich K, Sukthana Y, Pukrittayakamee S: Risk factors and clinical features associated with severe dengue infection in adults and children during the 2001 epidemic in Chonburi, Thailand. *Trop Med Int Health.* 2004;9:1022–1029.

100. Hegazi MA, Bakarman MA, Alahmadi TS, Butt NS, Alqahtani AM, Aljedaani BS, Almajnuni AH: Risk Factors and Predictors of Severe Dengue in Saudi Population in Jeddah, Western Saudi Arabia: A Retrospective Study. *Am J Trop Med Hyg.* 2020;102:613–621.

101. Perdomo-Celis F, Salgado DM, Narváez CF: Selective dysfunction of subsets of peripheral blood mononuclear cells during pediatric dengue and its relationship with clinical outcome. *Virology.* 2017;507:11–19.

102. Chaiyaratana W, Chuansumrit A, Atamasirikul K, Tangnararatchakit K: Serum ferritin levels in children with dengue infection. *Southeast Asian J Trop Med Public Health.* 2008;39:832–836.

103. Soundravally R, Sherin J, Agieshkumar BP, Daisy MS, Cleetus C, Narayanan P, Kadhiravan T, Sujatha S, Harichandrakumar KT: SERUM LEVELS OF COPPER AND IRON IN DENGUE FEVER. *Revista Do Instituto De Medicina Tropical De Sao Paulo.* 2015;57:315–320.

104. Liao B, Tang Y, Hu F, Zhou W, Yao X, Hong W, Wang J, Zhang X, Tang X, Zhang F: Serum levels of soluble vascular cell adhesion molecules may correlate with the severity of dengue virus-1 infection in adults. *Emerg Microbes Infect.* 2015;4:e24.

105. Trairatvorakul P, Chongsrisawat V, Ngamvasinont D, Asawarachun D, Nantasook J, Poovorawan Y: Serum nitric oxide in children with Dengue infection. *Asian Pac J Allergy Immunol.* 2005;23:115–119.

106. Balmaseda A, Hammond SN, Perez MA, Cuadra R, Solano S, Rocha J, Idiaquez W, Harris E: Short report: Assessment of the World Health Organization scheme for classification of dengue severity in Nicaragua. *Am J Trop Med Hyg.* 2005;73:1059–1062.

107. Soundravally R, Hoti SL: Significance of transporter associated with antigen processing 2 (TAP2) gene polymorphisms in susceptibility to dengue viral infection. *J Clin Immunol.* 2008;28:256–262.

108. Falconar AK, Romero-Vivas CM: Simple Prognostic Criteria can Definitively Identify Patients who Develop Severe Versus Non-Severe Dengue Disease, or Have Other Febrile Illnesses. *J Clin Med Res.* 2012;4:33–44.

109. Zhao L, Huang X, Hong W, Qiu S, Wang J, Yu L, Zeng Y, Tan X, Zhang F: Slow resolution of inflammation in severe adult dengue patients. *BMC Infect Dis.* 2016;16:291.

110. Wangdi K, Clements ACA, Du T, Nery SV: Spatial and temporal patterns of dengue infections in Timor-Leste, 2005-2013. *Parasites and Vectors.* 2018;11:9.

111. Rathi M, Masand R, Purohit A: STUDY OF DENGUE INFECTION IN RURAL RAJASTHAN. *J Evol Med Dent Sci.* 2015;4:6849–6859.

112. Cherupanakkal C, Ramachadrappa V, Kadhiravan T, Parameswaran N, Parija SC, Pillai AB, Rajendiran S: A Study on Gene Expression Profile of Endogenous Antioxidant Enzymes: CAT, MnSOD and GPx in Dengue Patients. *Indian J Clin Biochem.* 2017;32:437–445.

113. Bandyopadhyay D, Chattaraj S, Hajra A, Mukhopadhyay S, Ganesan V: A Study on Spectrum of Hepatobiliary Dysfunctions and Pattern of Liver Involvement in Dengue Infection. *J Clin Diagn Res.* 2016;10:Oc21–26.

114. Popper SJ, Gordon A, Liu M, Balmaseda A, Harris E, Relman DA: Temporal Dynamics of the Transcriptional Response to Dengue Virus Infection in Nicaraguan Children. *PLoS Negl Trop Dis.* 2012;6:e1966.

115. Chaudhary R, Khetan D, Sinha S, Sinha P, Sonker A, Pandey P, Das SS, Agarwal P, Ray V: Transfusion support to Dengue patients in a hospital based blood transfusion service in north India. *Transfus Apher Sci.* 2006;35:239–244.

116. Duangmala T, Lumbiganon P, Kosalaraksa P: Unusual clinical manifestations of dengue infection in children in a tertiary care hospital in northeast Thailand. *Asian Biomed.* 2014;8:97–103.

117. Park S, Srikiatkhachorn A, Kalayanarooj S, Macareo L, Green S, Friedman JF, Rothman AL: Use of structural equation models to predict dengue illness phenotype. *PLoS Negl Trop Dis.* 2018;12:e0006799.

118. Srivastava G, Chhavi N, Goel A: Validation of Serum Aminotransferases levels to define severe dengue fever in children. *Pediatr Gastroenterol, Hepatol Nutr.* 2018;21:289–296.

119. Saqib MA, Rafique I, Bashir S, Salam AA: A retrospective analysis of dengue fever case management and frequency of co-morbidities associated with deaths. *BMC research notes.* 2014, 7:205.

120. Mohamed NA, Abd El-Raoof E, Ibraheem HA: Respiratory manifestations of dengue fever in Taiz-Yemen. *Egypt J Chest Dis Tuberc.* 2013;62:319–323.

121. Ahmed FU, Mahmood CB, Sharma JD, Hoque SM, Zaman R, Hasan MS: Dengue and Dengue Haemorrhagic Fever in children during the 2000 outbreak in Chittagong, Bangladesh. Dengue bullet. 2001;25:33–39.

122. Lam PK, Ngoc TV, Thu Thuy TT, Hong Van NT, Nhu Thuy TT, Hoai Tam DT, Dung NM, Hanh Tien NT, Thanh Kieu NT, Simmons C *et al*: The value of daily platelet counts for predicting dengue shock syndrome: Results from a prospective observational study of 2301 Vietnamese children with dengue. *PLoS Negl Trop Dis.* 2017;11:e0005498.

123. Wong JG, Gan VC, Ng EL, Leo YS, Chan SP, Choo R, Lye DC: Self-reported pain intensity with the numeric reporting scale in adult dengue. *PloS one.* 2014;9:e96514.

124. Hoang LT, Lynn DJ, Henn M, Birren BW, Lennon NJ, Le PT, Duong KT, Nguyen TT, Mai LN, Farrar JJ *et al*: The early whole-blood transcriptional signature of dengue virus and features associated with progression to dengue shock syndrome in Vietnamese children and young adults. *J Virol.* 2010;84:12982–12994.

125. Andries AC, Duong V, Cappelle J, Ong S, Kerleguer A, Ly S, Tarantola A, Horwood PF, Sakuntabhai A, Dussart P *et al*: Proteinuria during dengue fever in children. *Int J Infect Dis.* 2017;55:38–44.

126. Furuta T, Murao LA, Lan NT, Huy NT, Huong VT, Thuy TT, Tham VD, Nga CT, Ha TT, Ohmoto Y *et al*: Association of mast cell-derived VEGF and proteases in Dengue shock syndrome. *PLoS Negl Ttrop Dis.* 2012;6:e1505.

127. S SS, Pillai AB, Ramachandrappa VS, T K, Dhodapkar R, Kah J, Rajendiran S: Increased serum levels of macrophage activation marker sCD163 in Dengue patients. *J Clin Virol.* 2017;86:62–67.

128. Mairuhu AT, Peri G, Setiati TE, Hack CE, Koraka P, Soemantri A, Osterhaus AD, Brandjes DP, van der Meer JW, Mantovani A *et al*: Elevated plasma levels of the long pentraxin, pentraxin 3, in severe dengue virus infections. *J Med Virol.* 2005;76:547–552.

129. Vuong NL, Manh DH, Mai NT, Phuc le H, Luong VT, Quan VD, Thuong NV, Lan NT, Nhon CT, Mizukami S *et al*: Criteria of "persistent vomiting" in the WHO 2009 warning signs for dengue case classification. *Trop Med Health.* 2016;44:14.

130. Pothapregada S, Kamalakannan B, Thulasingham M: Risk factors for shock in children with dengue fever. *Indian J Crit Care Med.* 2015;19:661–664.

131. Thanachartwet V, Wattanathum A, Sahassananda D, Wacharasint P, Chamnanchanunt S, Khine Kyaw E, Jittmittraphap A, Naksomphun M, Surabotsophon M, Desakorn V: Dynamic Measurement of Hemodynamic Parameters and Cardiac Preload in Adults with Dengue: A Prospective Observational Study. *PloS one.* 2016;11:e0156135.

132. May WL, Kyaw MP, Blacksell SD, Pukrittayakamee S, Chotivanich K, Hanboonkunupakarn B, Thein KN, Lim CS, Thaipadungpanit J, Althaus T *et al*: Impact of glucose-6-phosphate dehydrogenase deficiency on dengue infection in Myanmar children. *PloS one.* 2019;14:e0209204.

133. Machain-Williams C, Raga E, Baak-Baak CM, Kiem S, Blitvich BJ, Ramos C: Maternal, Fetal, and Neonatal Outcomes in Pregnant Dengue Patients in Mexico. *BioMed Res Int.* 2018;2018:9643083.

134. de Kruif MD, Setiati TE, Mairuhu AT, Koraka P, Aberson HA, Spek CA, Osterhaus AD, Reitsma PH, Brandjes DP, Soemantri A *et al*: Differential gene expression changes in children with severe dengue virus infections. *PLoS Negl Trop Dis.* 2008;2:e215.

135. Zhao H, Qiu S, Hong WX, Song KY, Wang J, Yang HQ, Deng YQ, Zhu SY, Zhang FC, Qin CF: Dengue Specific Immunoglobulin A Antibody is Present in Urine and Associated with Disease Severity. *Sci Rep.* 2016;6:27298.

136. Balakrishna Pillai A, Cherupanakkal C, Immanuel J, Saravanan E, Eswar Kumar V, A A, Kadhiravan T, Rajendiran S: Expression Pattern of Selected Toll-like Receptors (TLR's) in the PBMC's of Severe and Non-severe Dengue Cases. *Immunol Invest.* 2020;49:443–452.

137. Marin-Palma D, Sirois CM, Urcuqui-Inchima S, Hernandez JC: Inflammatory status and severity of disease in dengue patients are associated with lipoprotein alterations. *PloS one.* 2019;14:e0214245.

138. Cherupanakkal C, Samadanam DM, Muthuraman KR, Ramesh S, Venkatesan A, Balakrishna Pillai AK, Rajendiran S: Lipid peroxidation, DNA damage, and apoptosis in dengue fever. *IUBMB life.* 2018;70:1133–1143.

139. Utama IMGDL, Agustini NMA: The role of macrophage Migration Inhibitory Factor (MIF) in pediatric dengue infection at Sanglah Hospital, Bali, Indonesia. *Bali Med J*. 2020;9:224–228.

140. Gibson G, Souza-Santos R, Pedro AS, Honório NA, M SC: Occurrence of severe dengue in Rio de Janeiro: an ecological study. *Rev Soc Bras Med Trop.* 2014;47:684–691.

141. Prasad D, Bhriguvanshi A: Clinical Profile, Liver Dysfunction and Outcome of Dengue Infection in Children: A Prospective Observational Study. *Pediatr Infect Dis J.* 2020;39:97–101.

142. Uddin MN, Hossain MM, Dastider R, Hasan Z, Ahmed Z, Dhar DK: Clinico-pathological profile of dengue syndrome: an experience in a tertiary care hospital, Dhaka, Bangladesh. *Mymensingh Med J.* 2014;23:774–780.

143. Thanachartwet V, Oer-Areemitr N, Chamnanchanunt S, Sahassananda D, Jittmittraphap A, Suwannakudt P, Desakorn V, Wattanathum A: Identification of clinical factors associated with severe dengue among Thai adults: a prospective study. *BMC Infect Dis.* 2015;15:420.
