# Supplementary material for: Clinical predictors of severe dengue: a systematic review and meta-analysis
Source: Infect Dis Poverty. 2021 Oct 9;10:123. doi: 10.1186/s40249-021-00908-2 (PMC8501593; doi:10.1186/s40249-021-00908-2)

*Note:* In all these figures, stacked column charts were used to present 36 bias safeguards and seven standards for all 143 studies included in the review. Stacks are sized and numbered from 1 to 7 to represent standards for equal recruitment, equal retention, equal ascertainment, equal implementation, equal prognosis, sufficient analysis and temporal precedence respectively. The y-axis represents the total safeguard counts (i.e., overall quality score).

Additional file 4-A
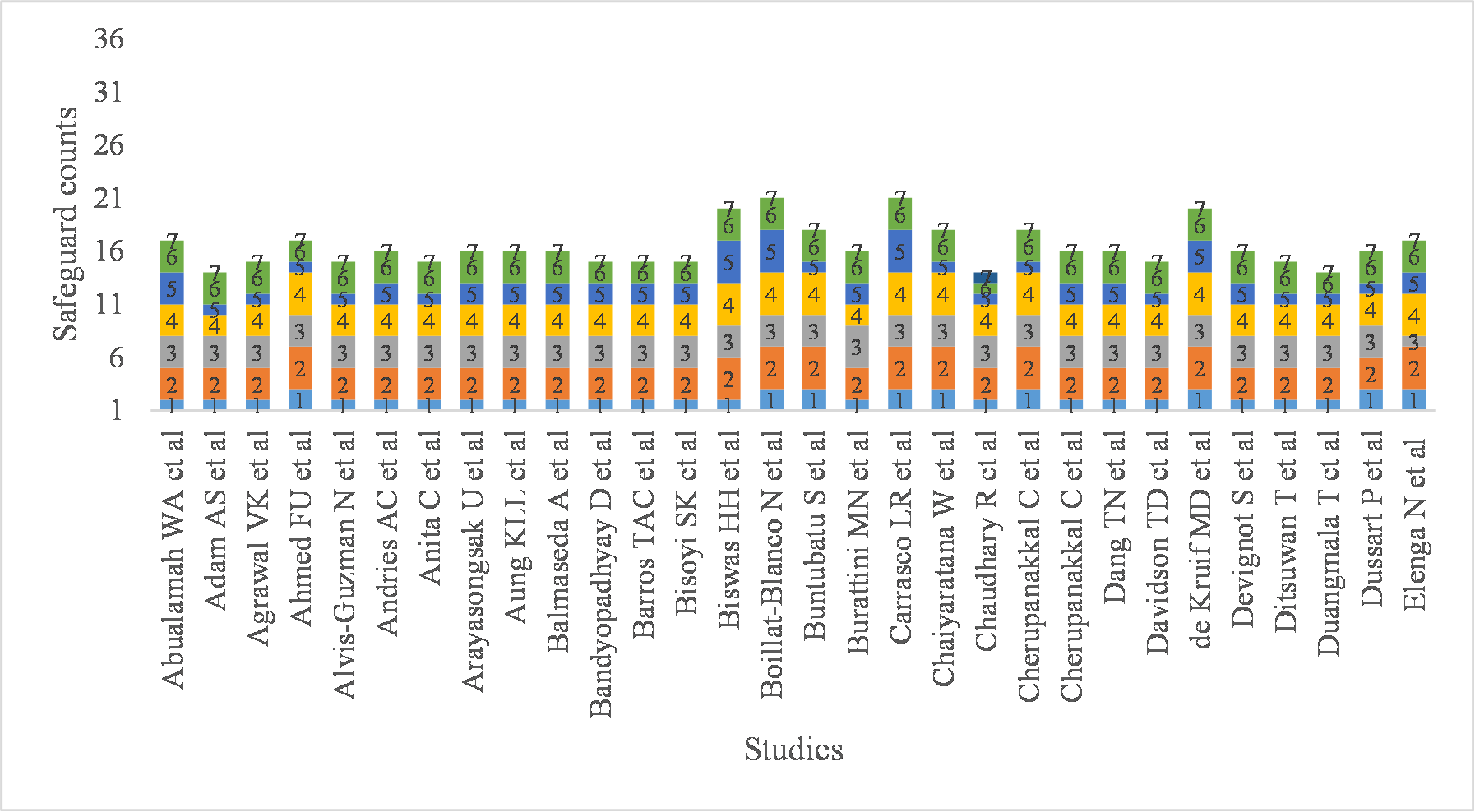


Additional file 4-B
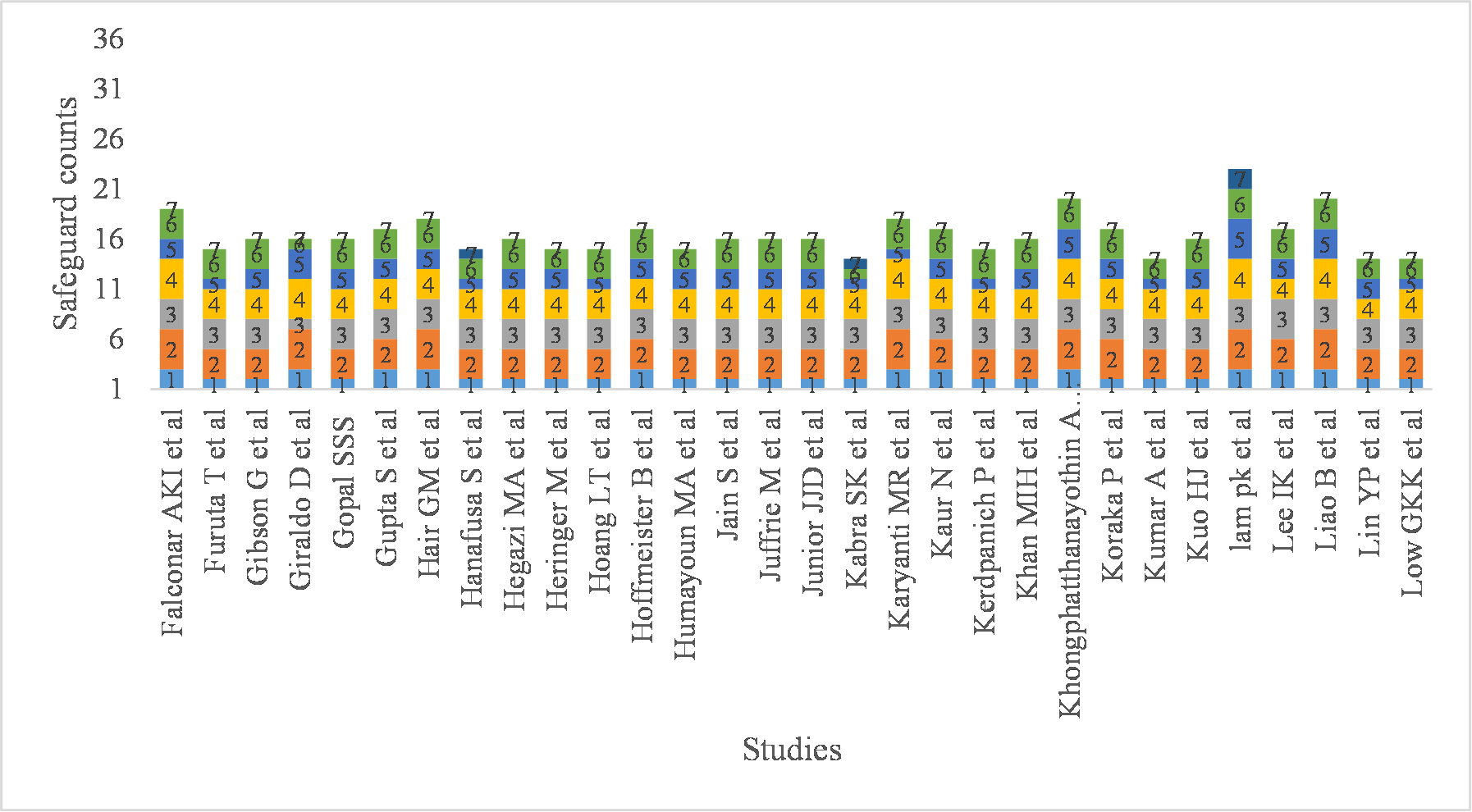


Additional file 4-C


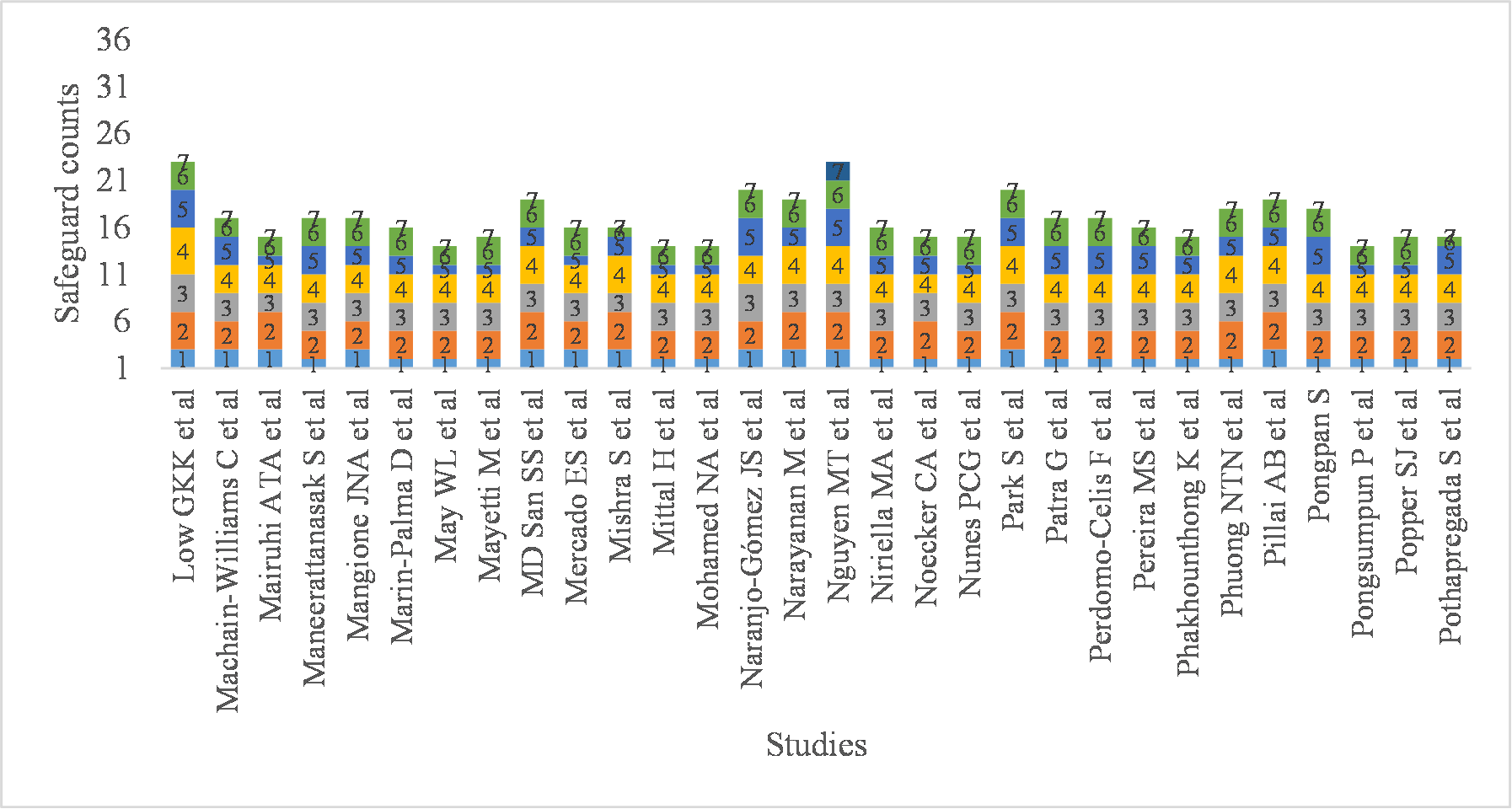


Additional file 4-D


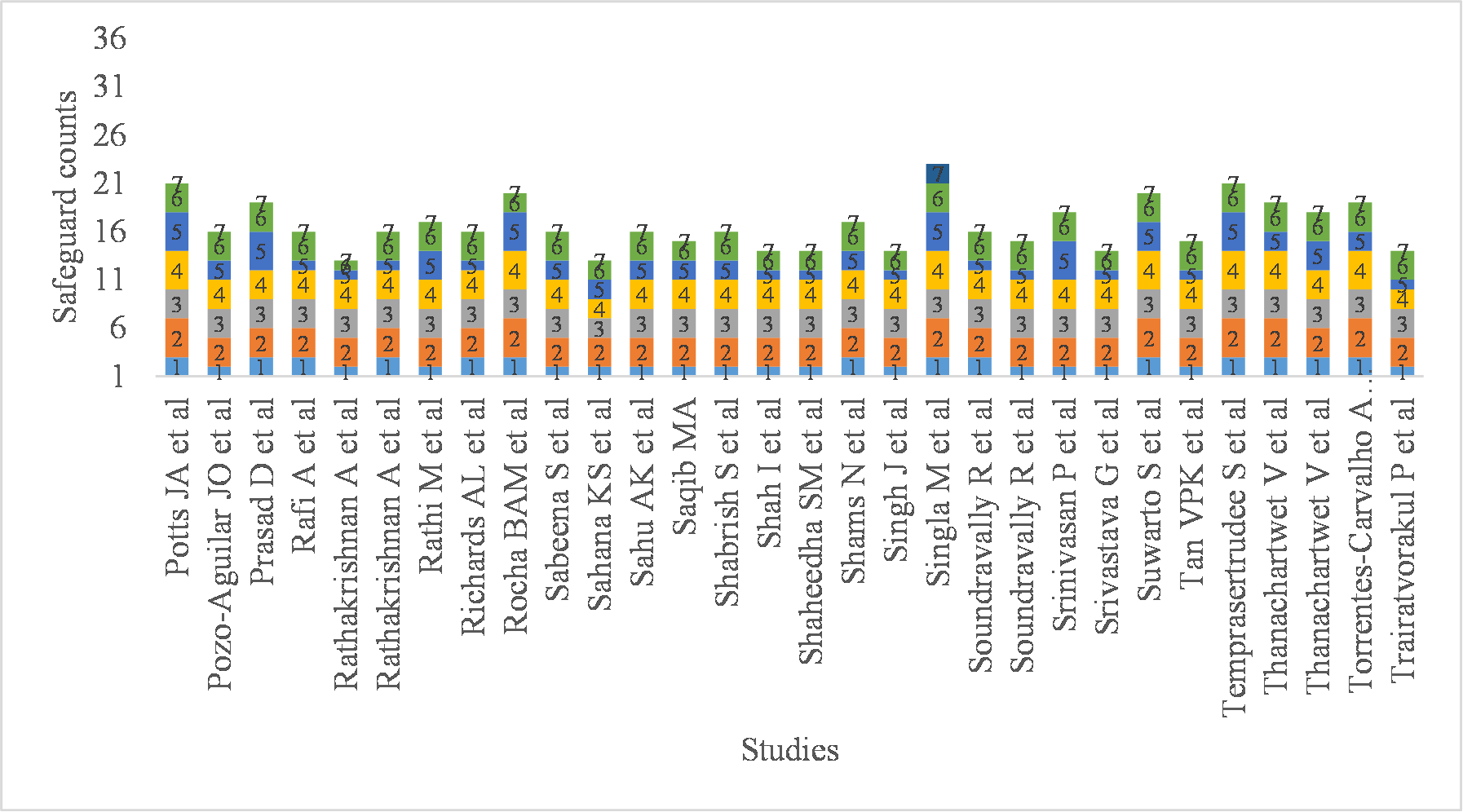


Additional file 4-E


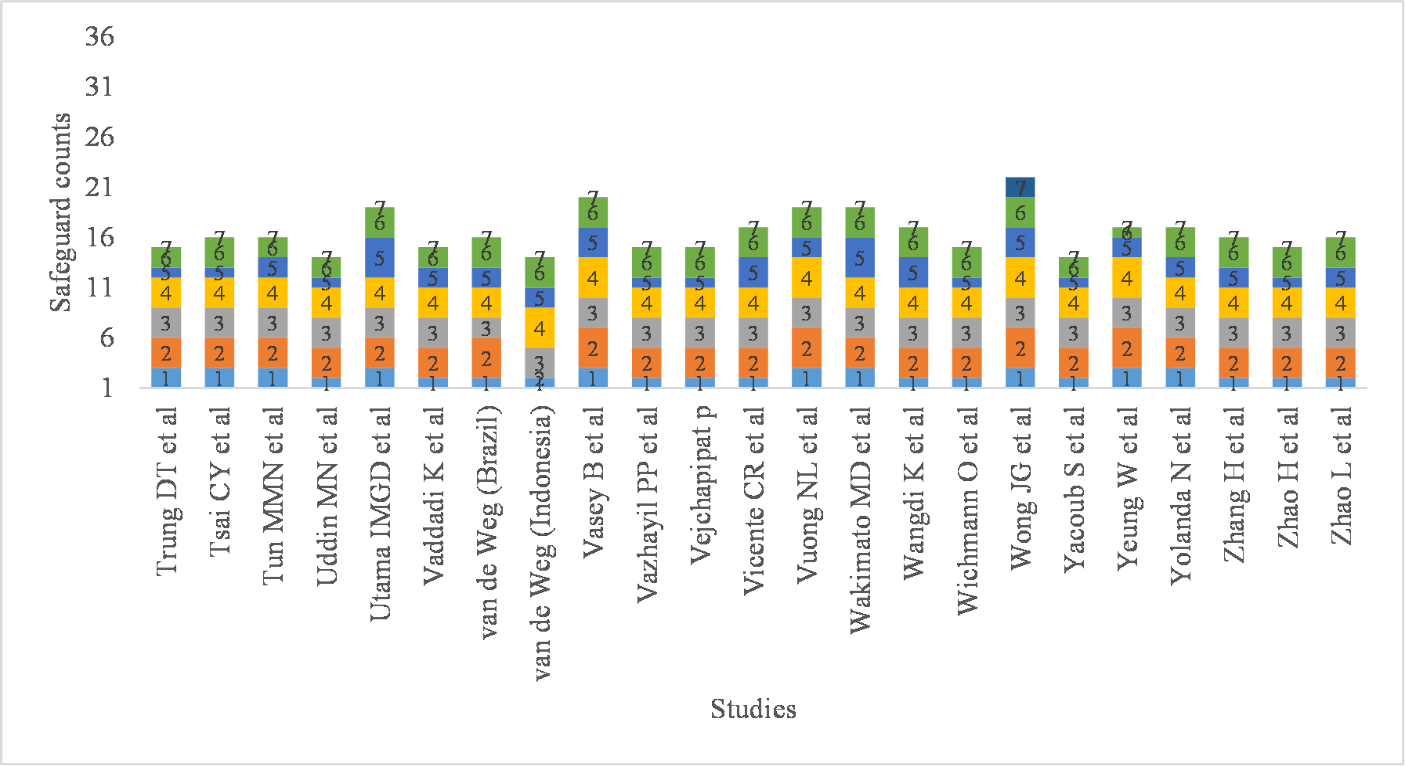

Supplement: Supplementary file 4 — Additional file 4. Quality assessment of the studies using a MASTER Scale [file 40249_2021_908_MOESM4_ESM.docx]
