# Supplementary material for: Clinical predictors of severe dengue: a systematic review and meta-analysis
Source: Infect Dis Poverty. 2021 Oct 9;10:123. doi: 10.1186/s40249-021-00908-2 (PMC8501593; doi:10.1186/s40249-021-00908-2)

Additional file 5 - Children


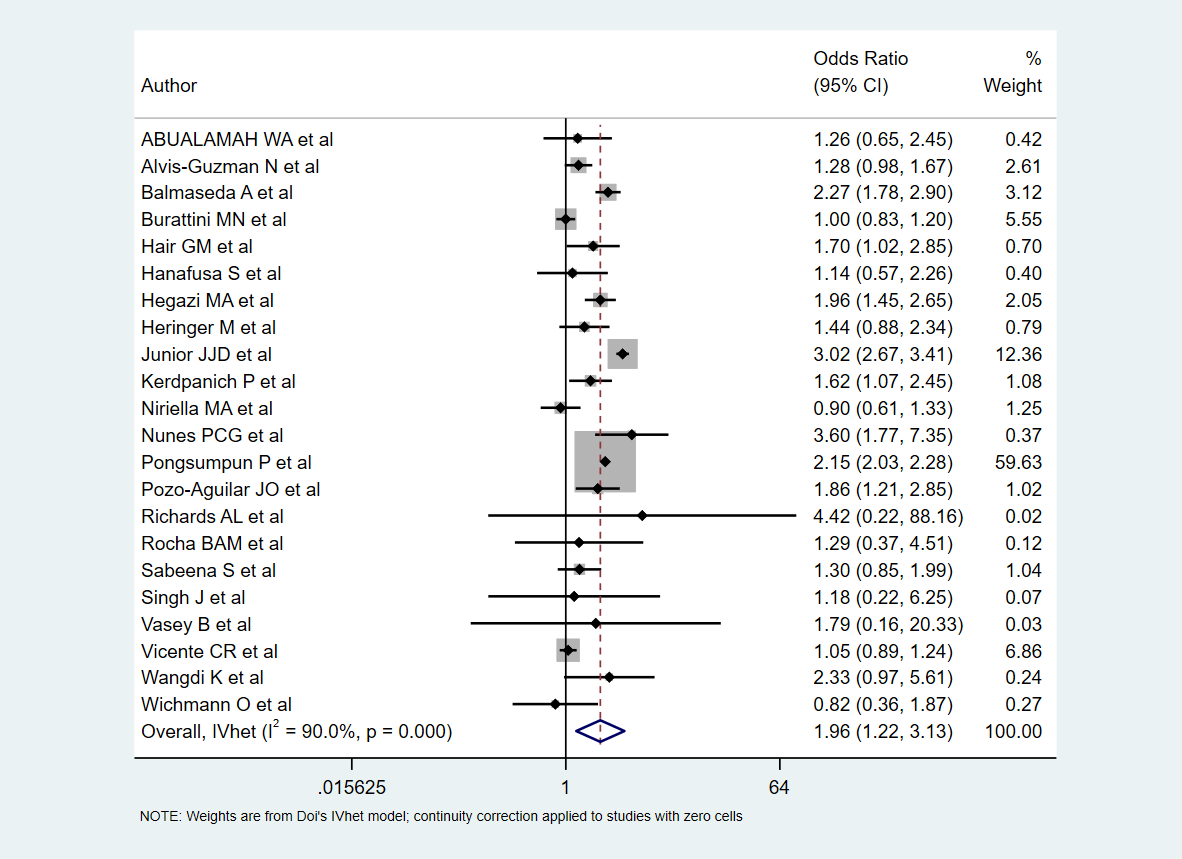


Additional file 5 - Infection


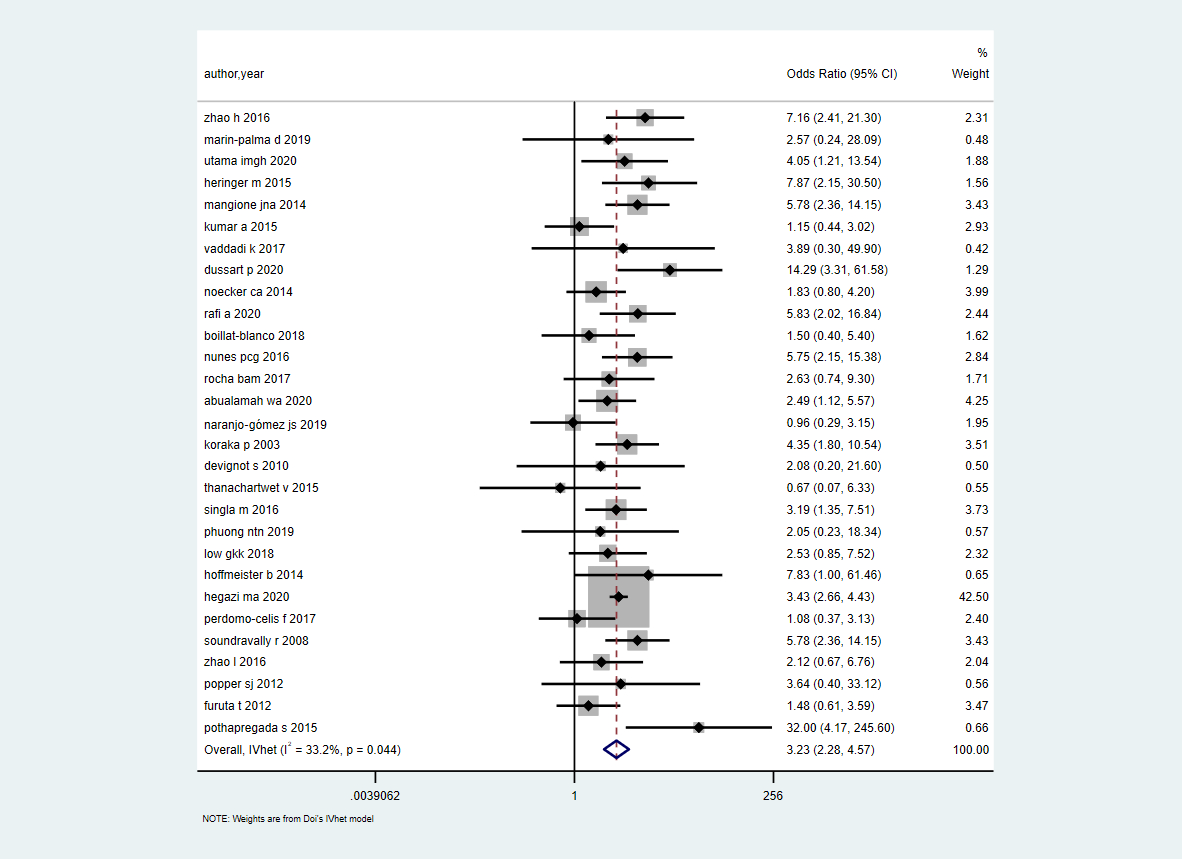


Additional file 5 - Diabetes


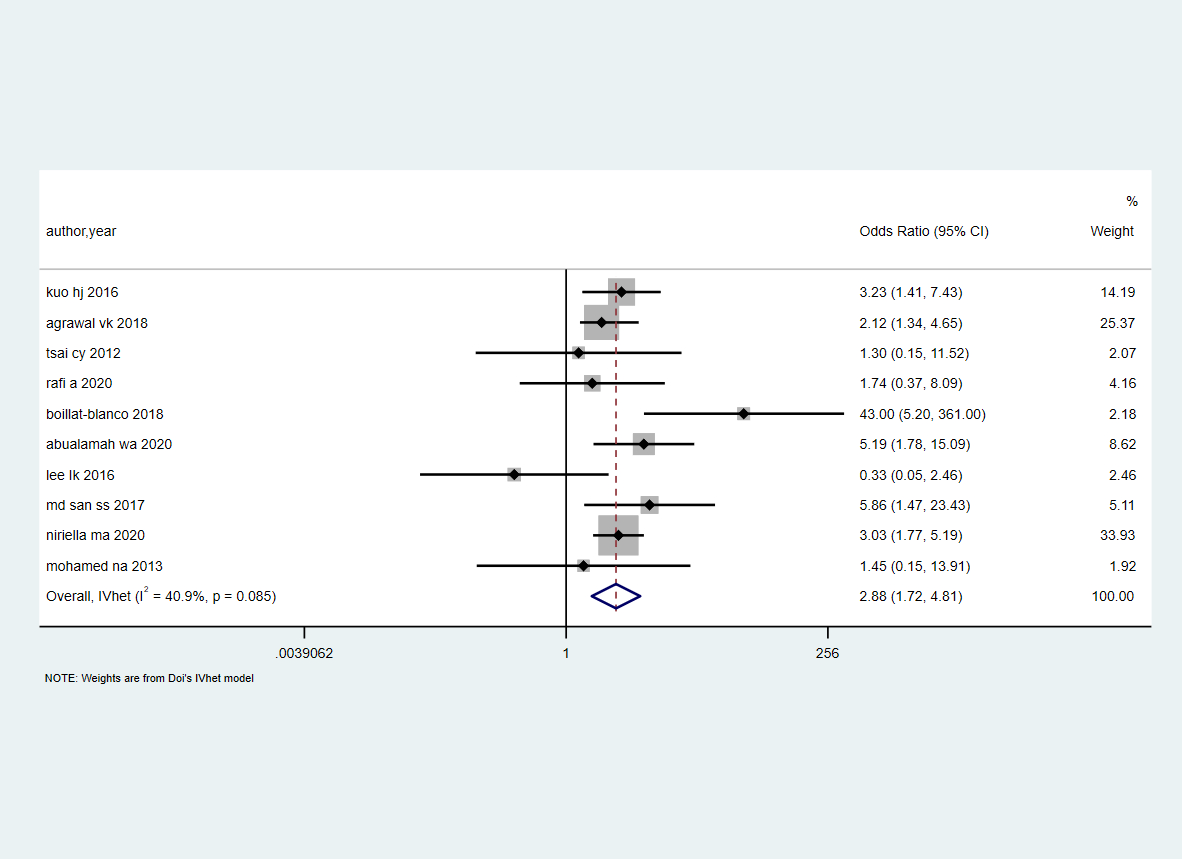


Additional file 5 - Cardiovascular disease


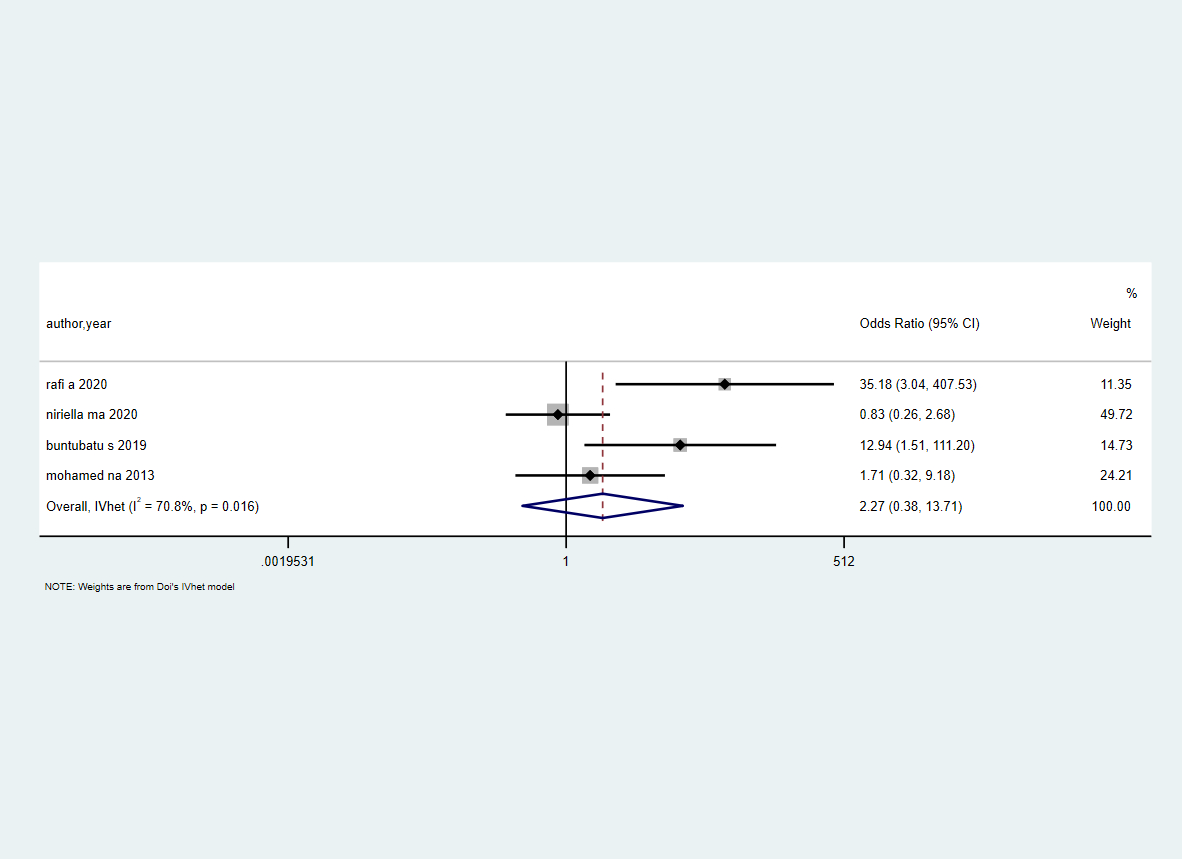


Additional file 5 - Obesity


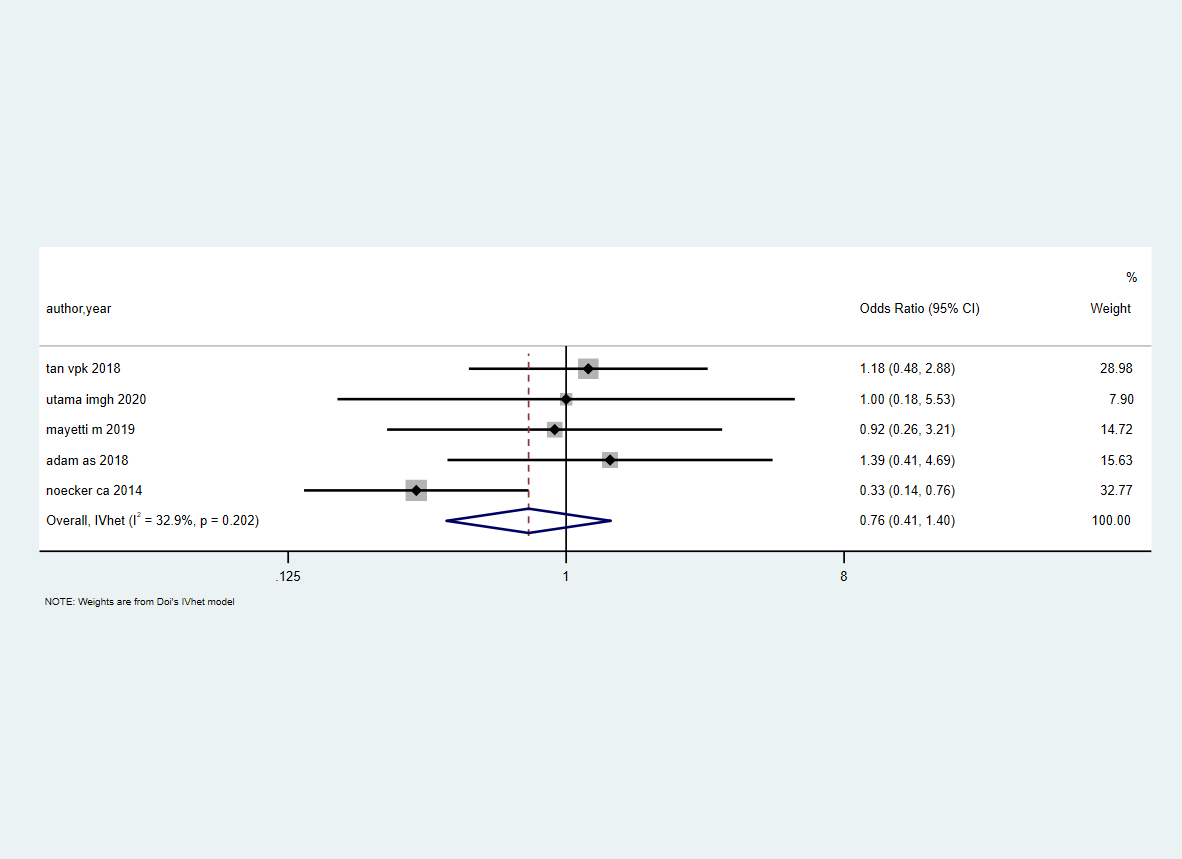


Additional file 5 - Renal disease


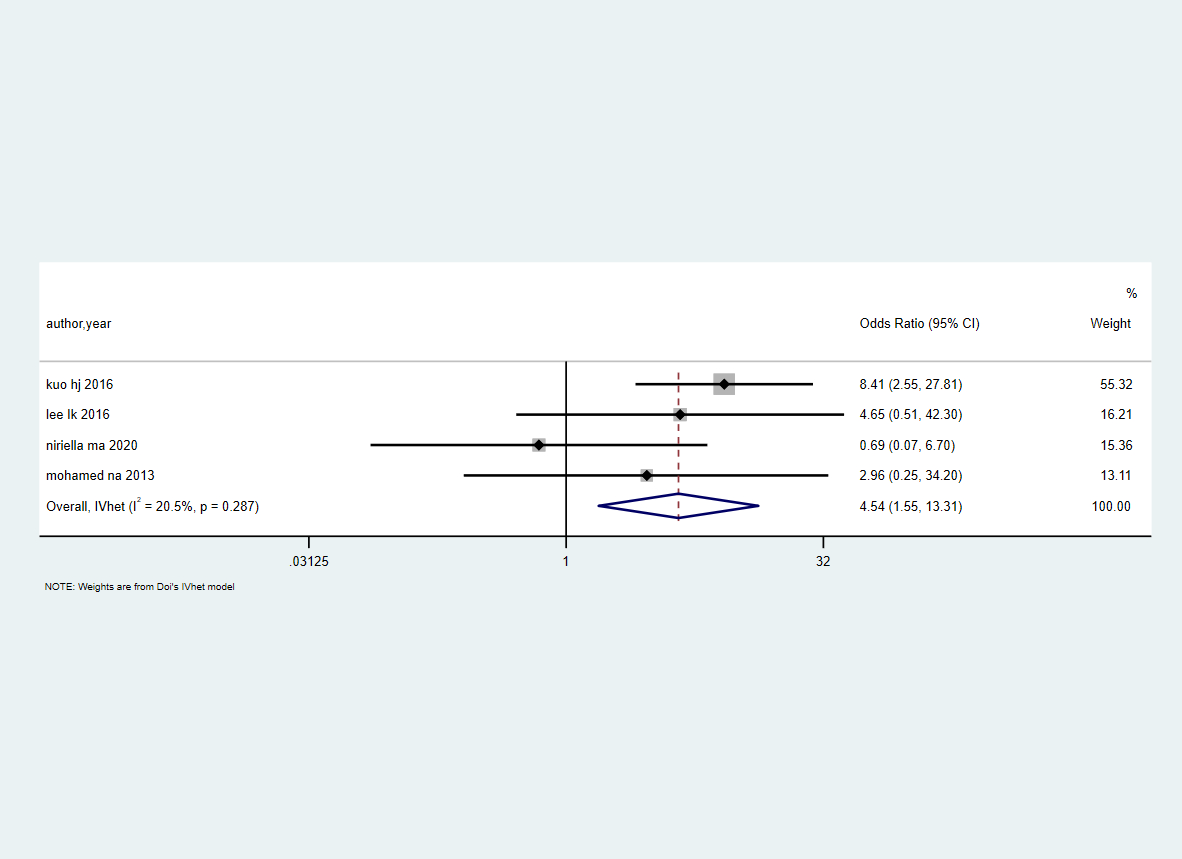


Additional file 5 - Hypertension


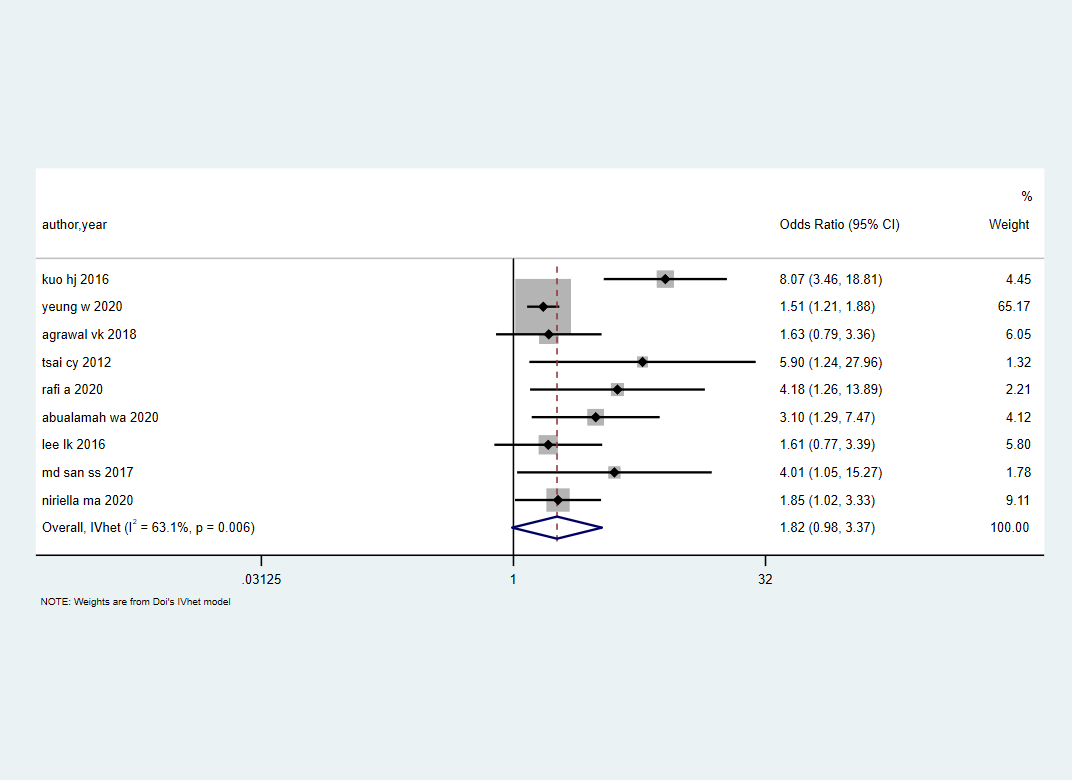


Additional file 5 - ↑Hct & ↓Plt


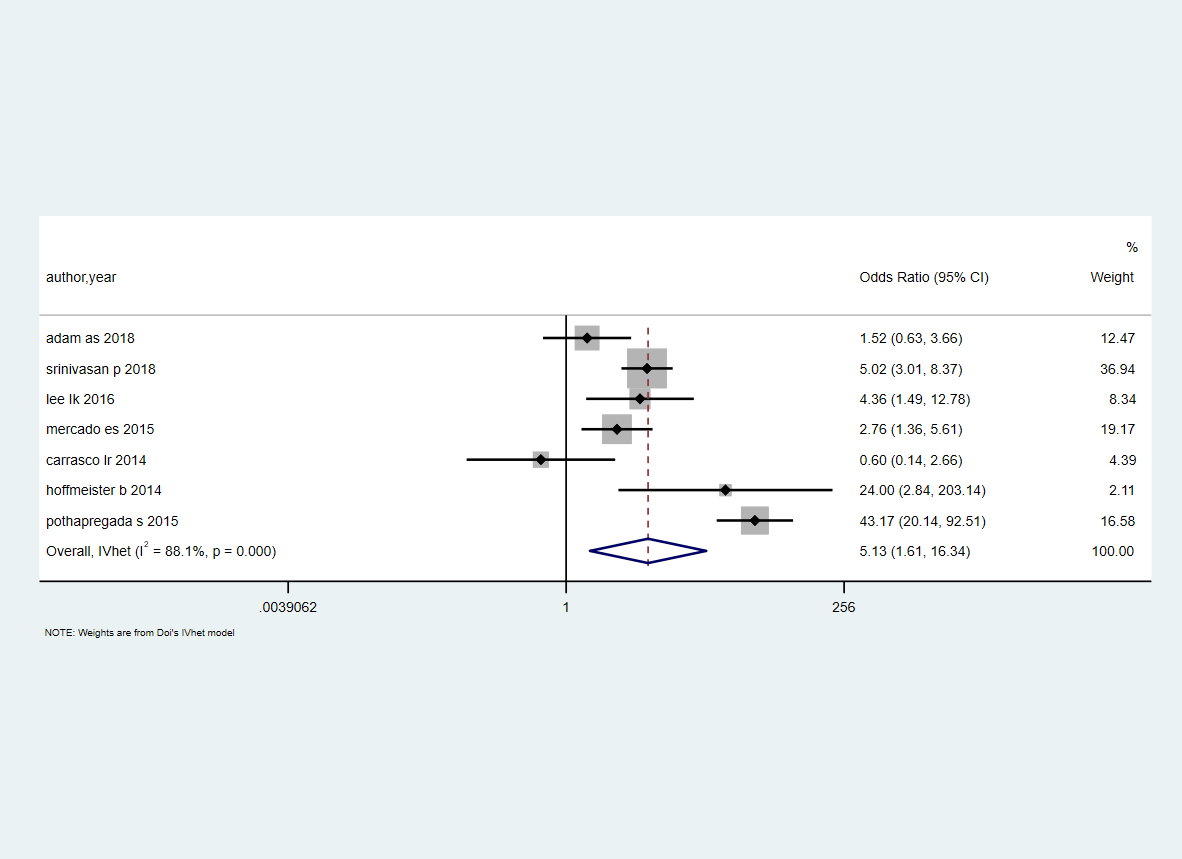


Additional file 5 - Abdominal pain


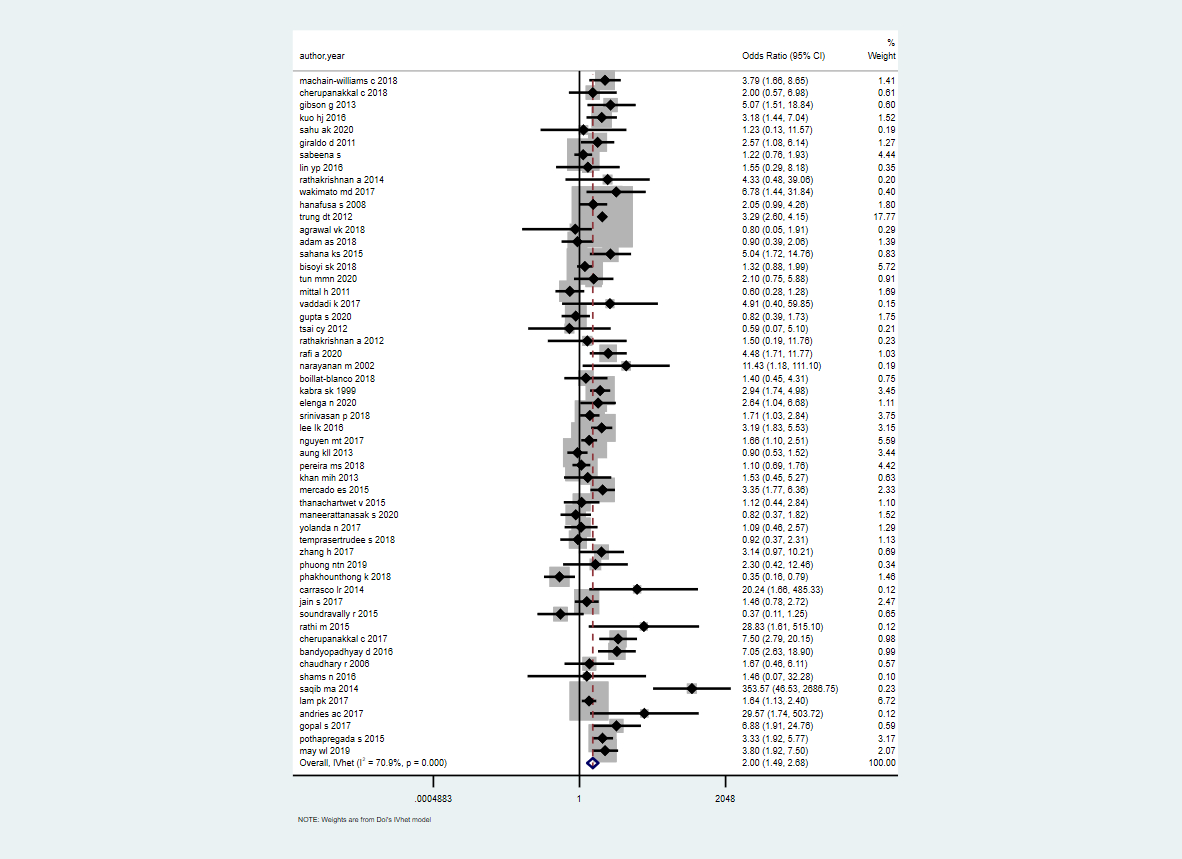


Additional file 5 - Vomiting


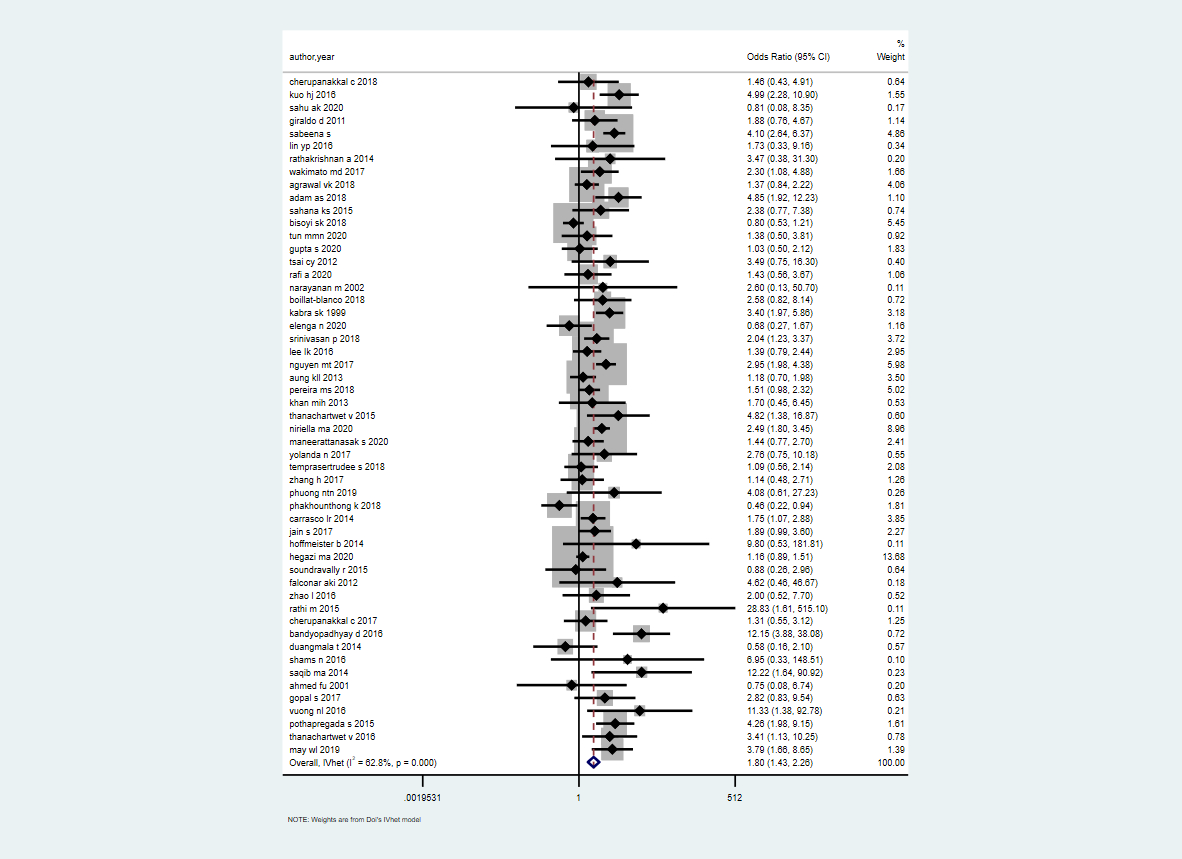


Additional file 5 - Lethargy


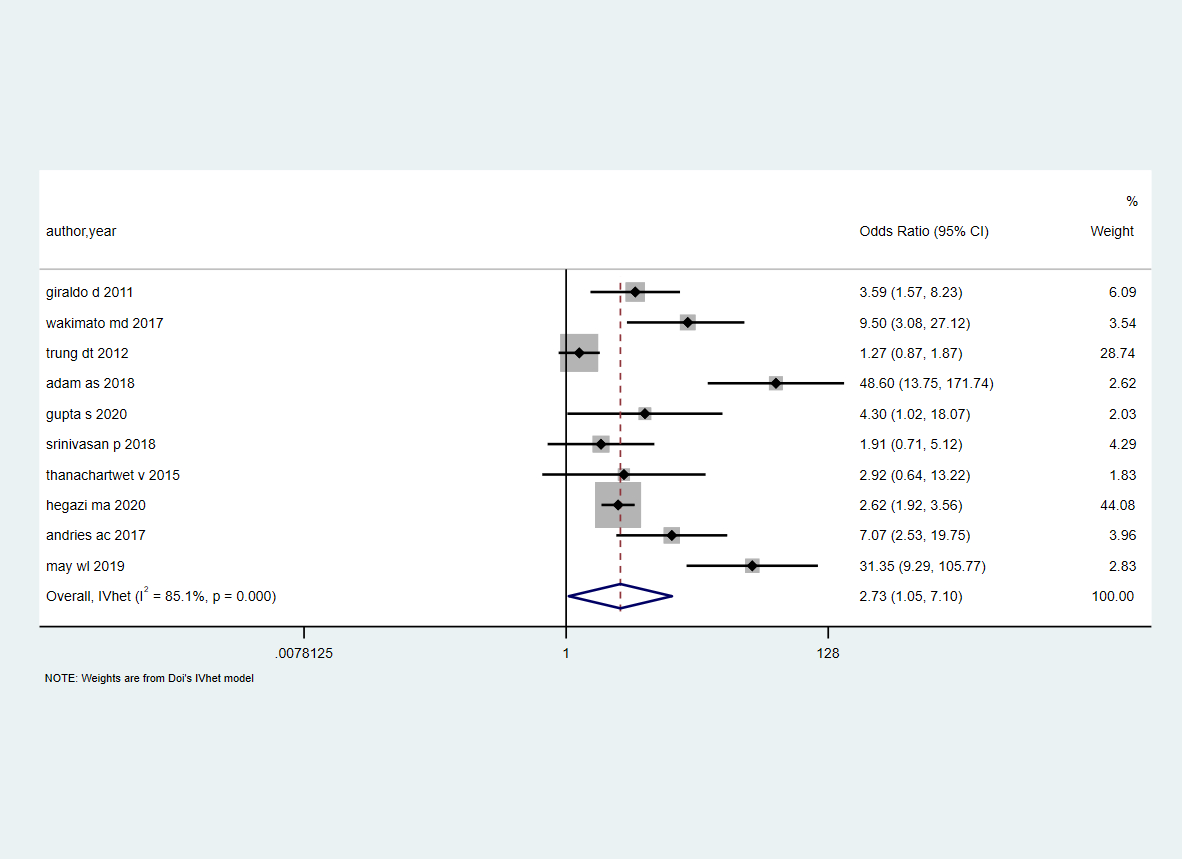


Additional file 5 - Hepatomegaly


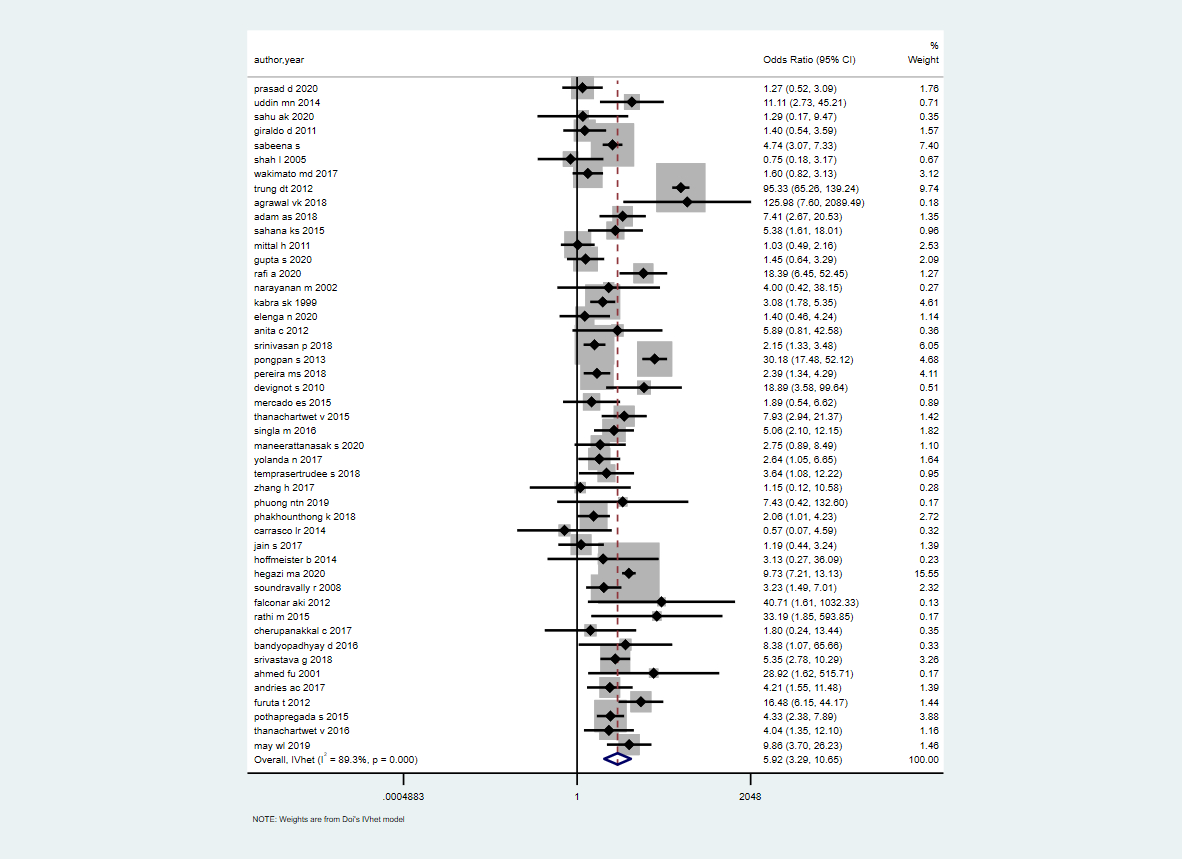


Additional file 5 - Ascitis


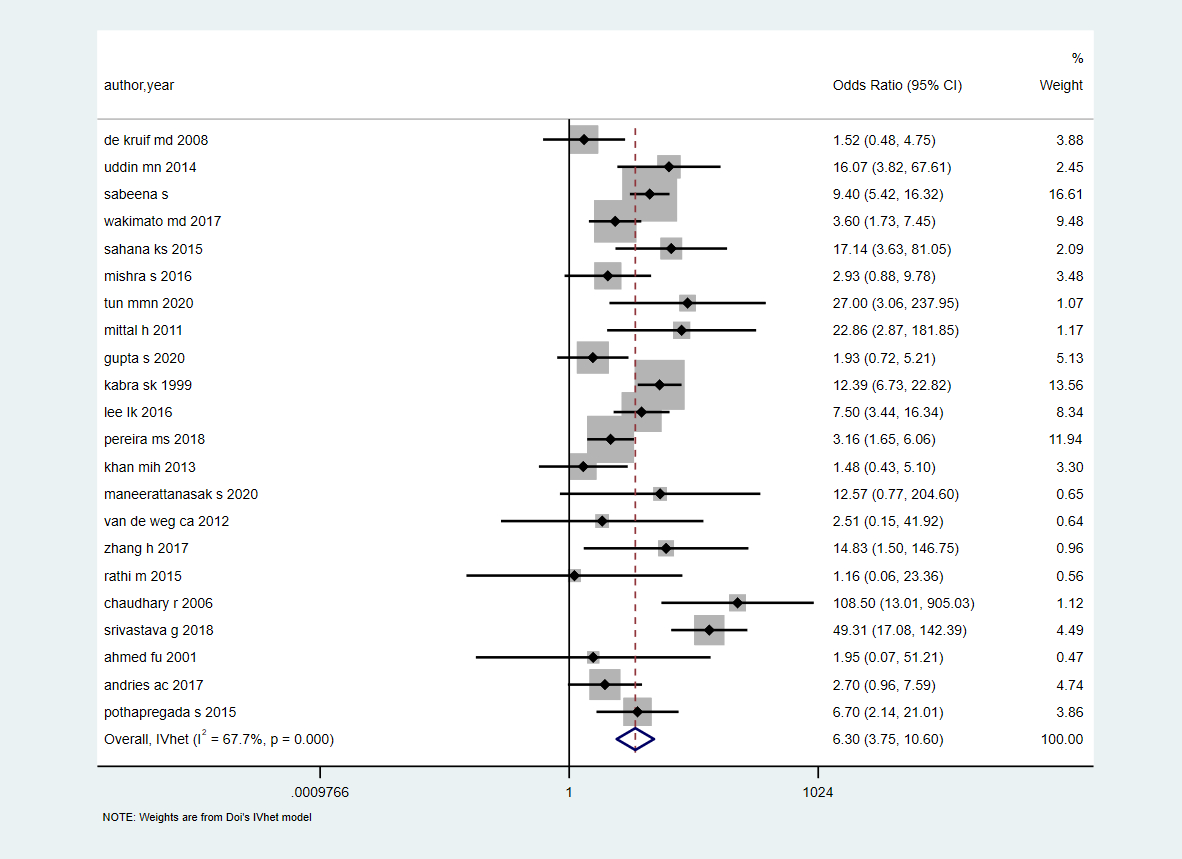


Additional file 5 - Pleural effusion


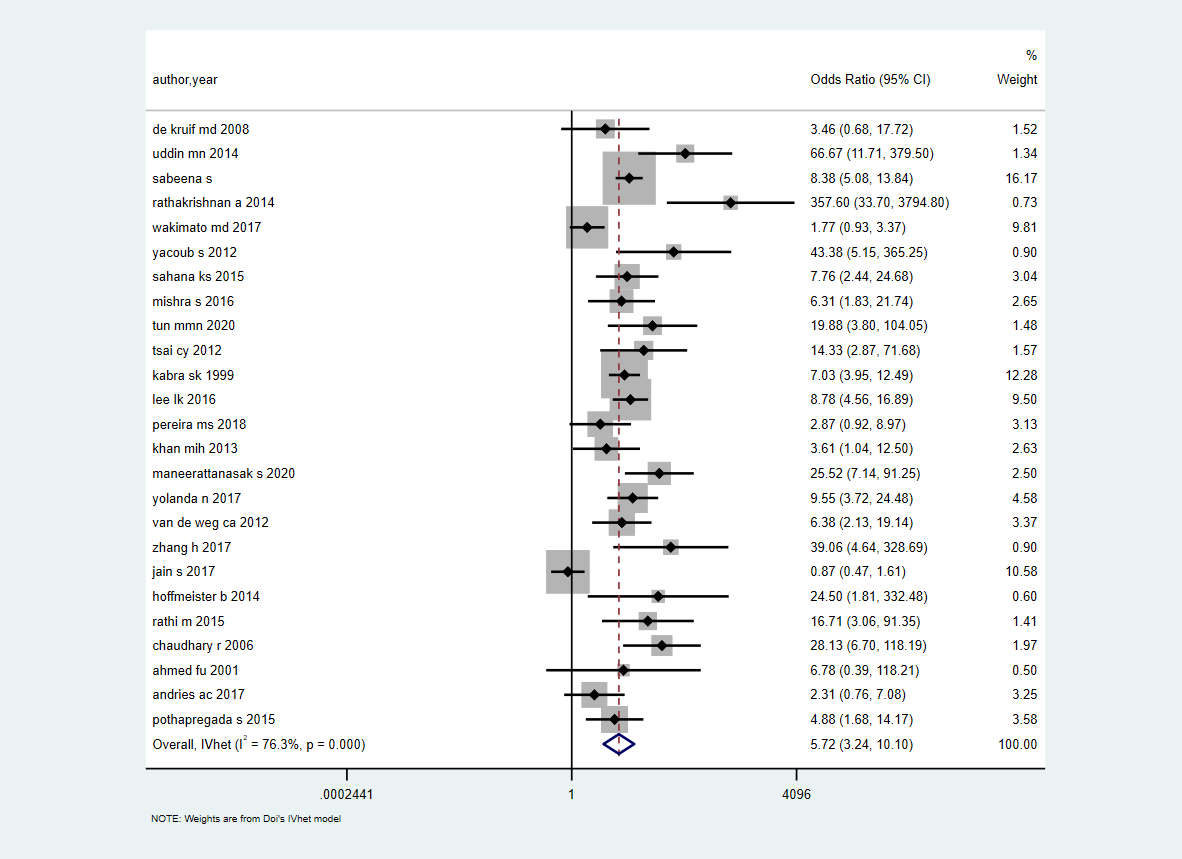


Additional file 5 - Gum bleeding


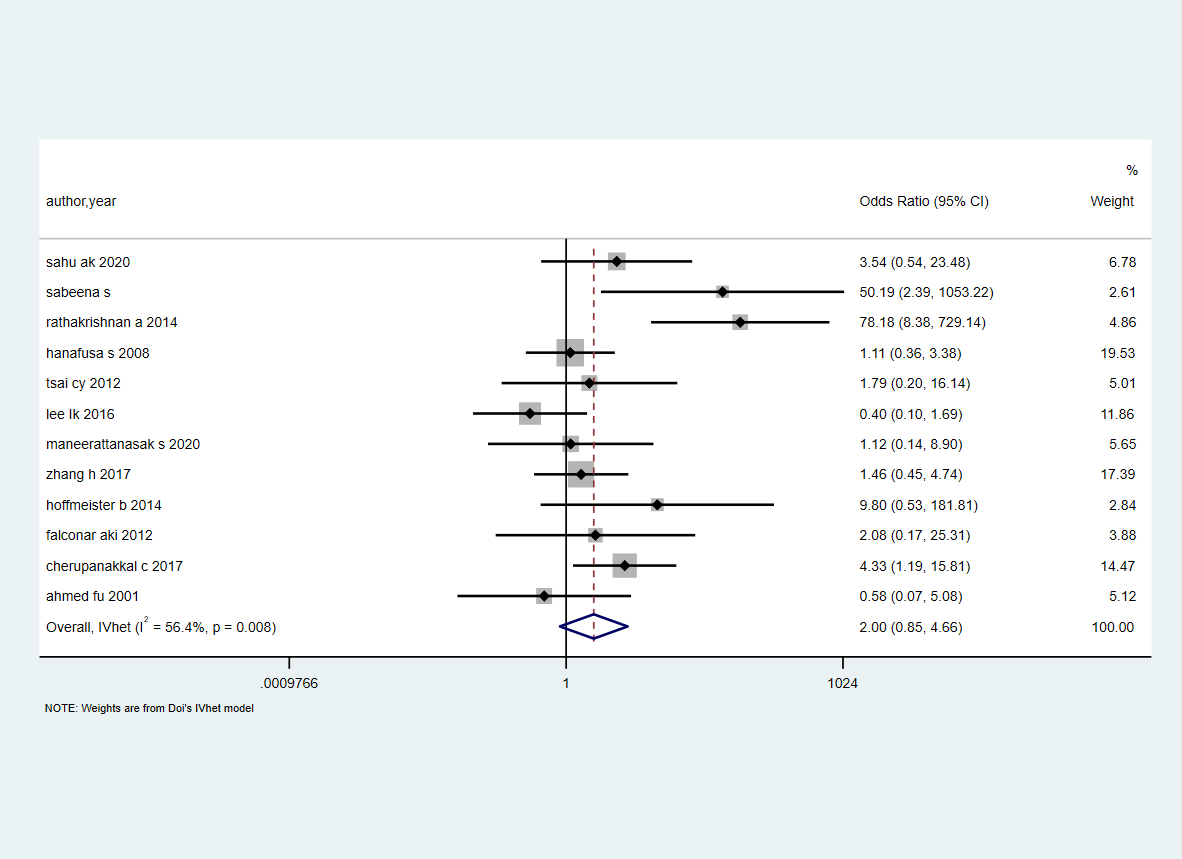


Additional file 5 - Epistaxis


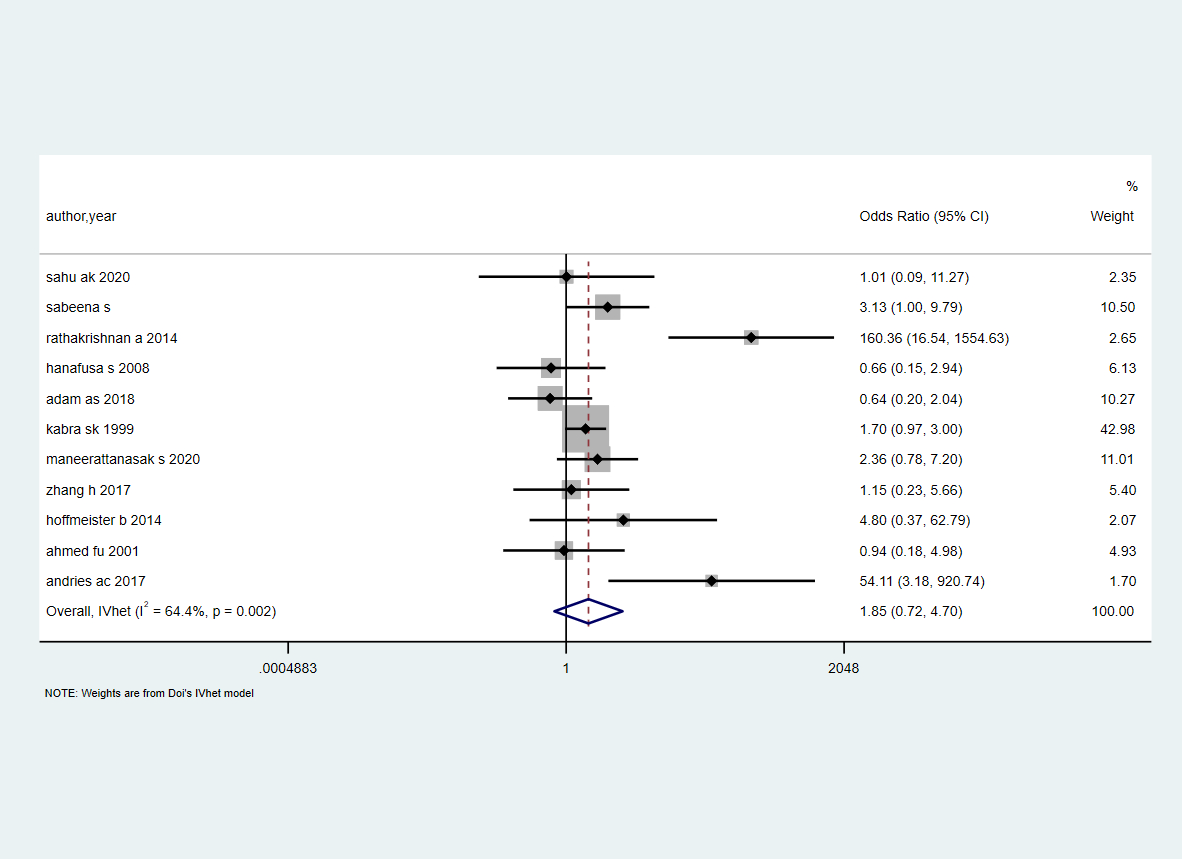


Additional file 5 - Hemetemesis


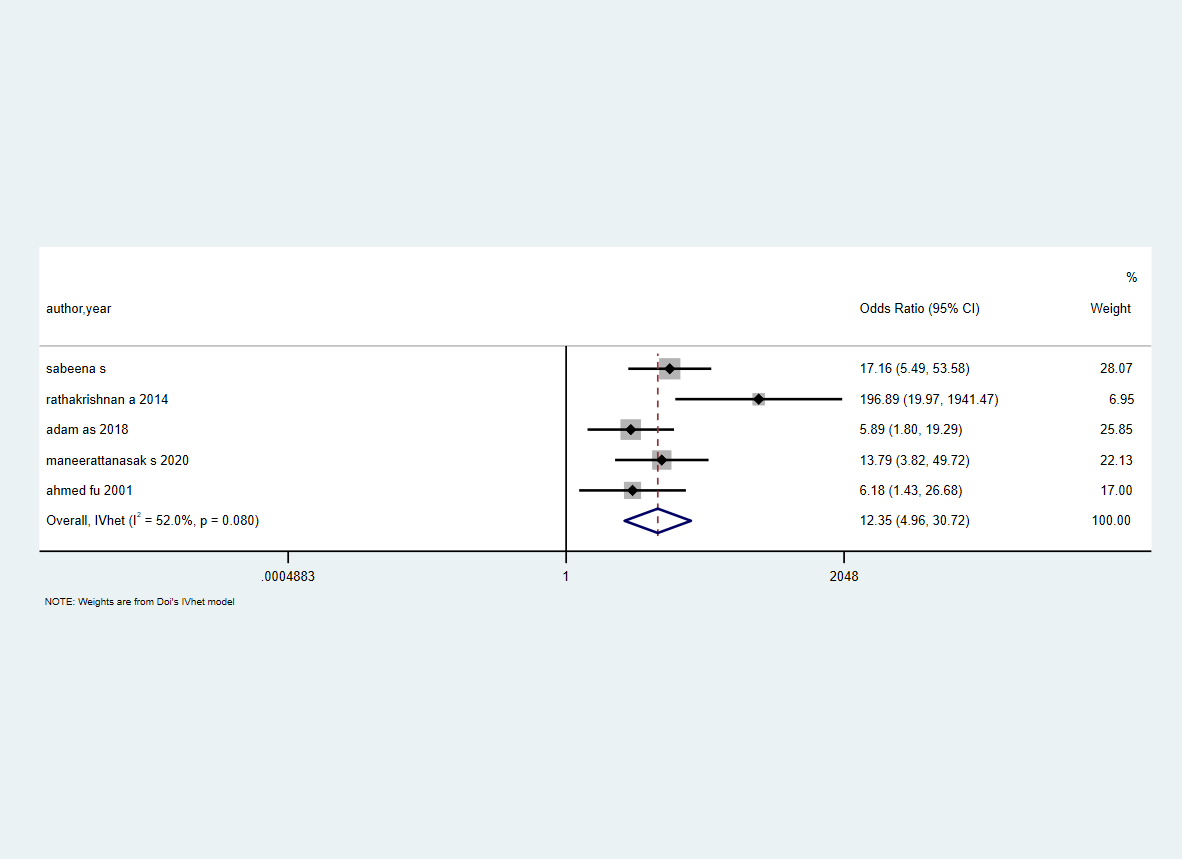


Additional file 5 - Melena


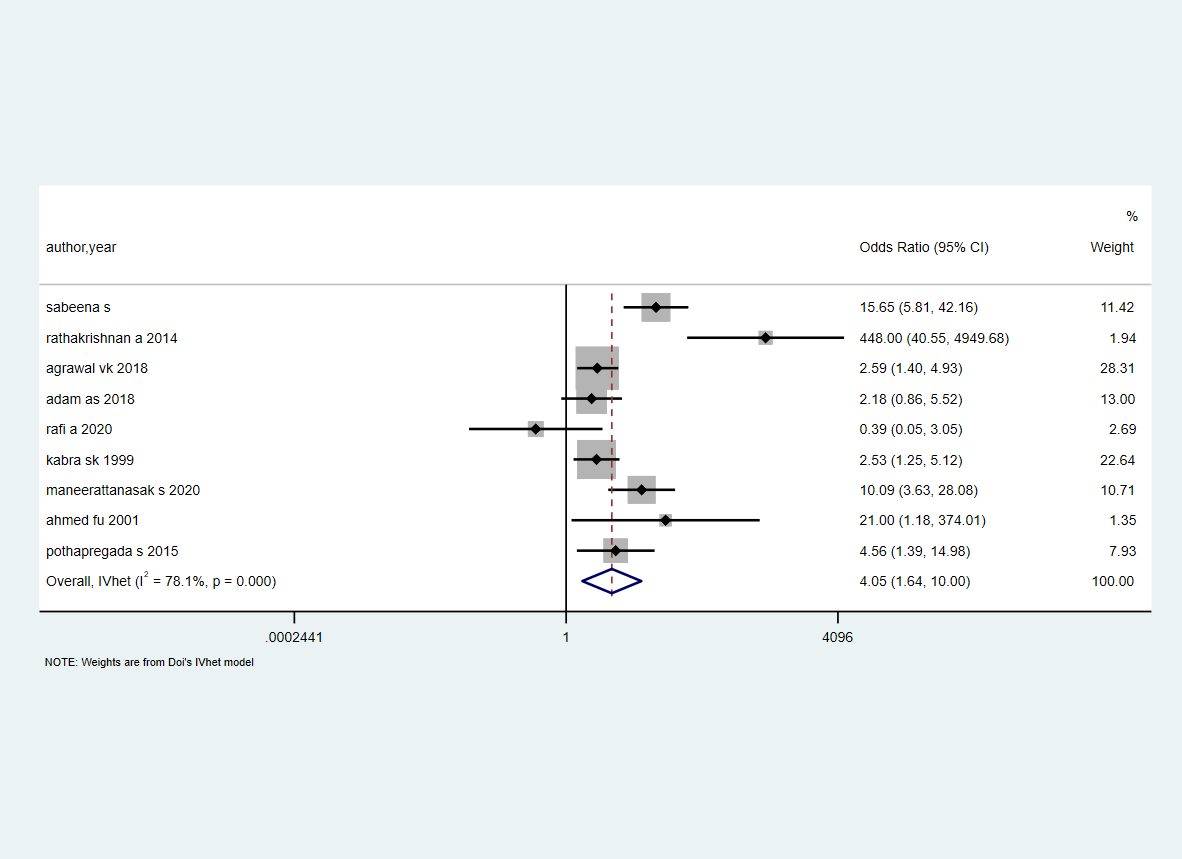


Additional file 5 - Skin bleeding


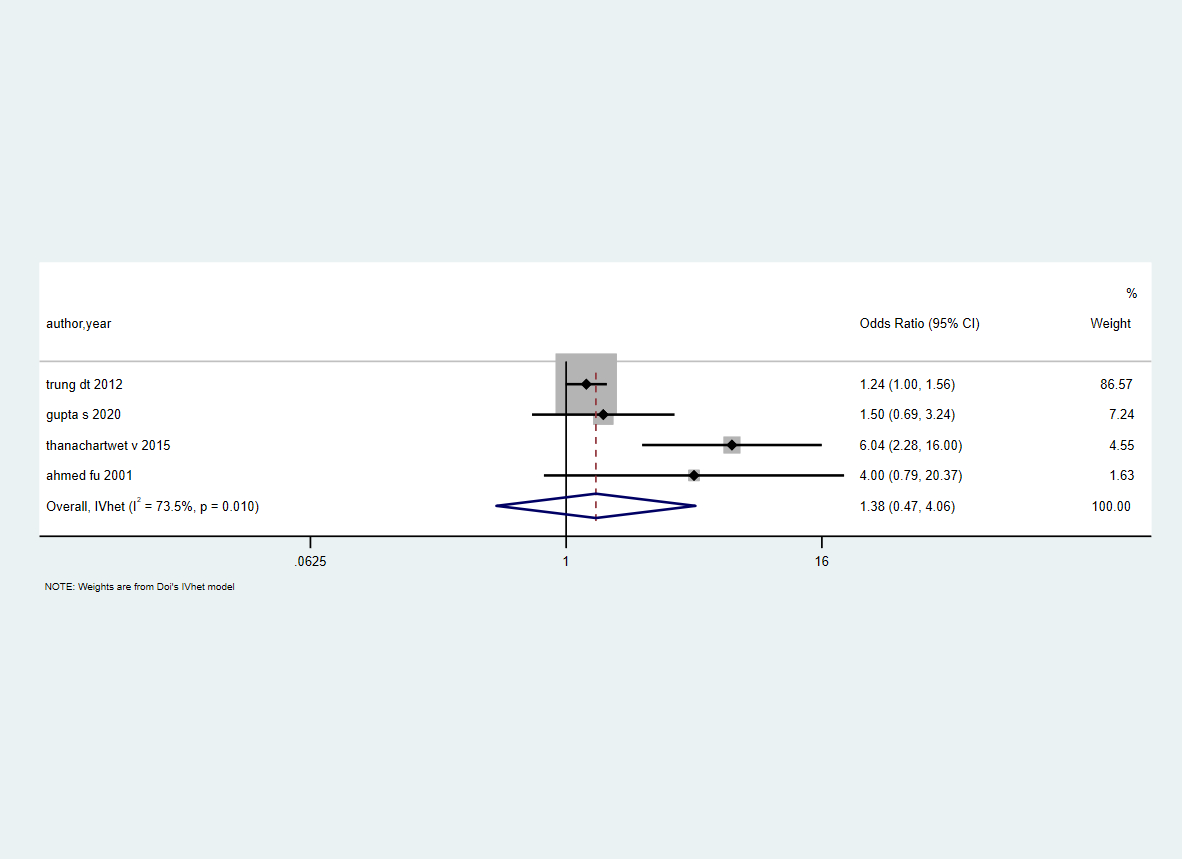


Additional file 5 - Gum bleeding


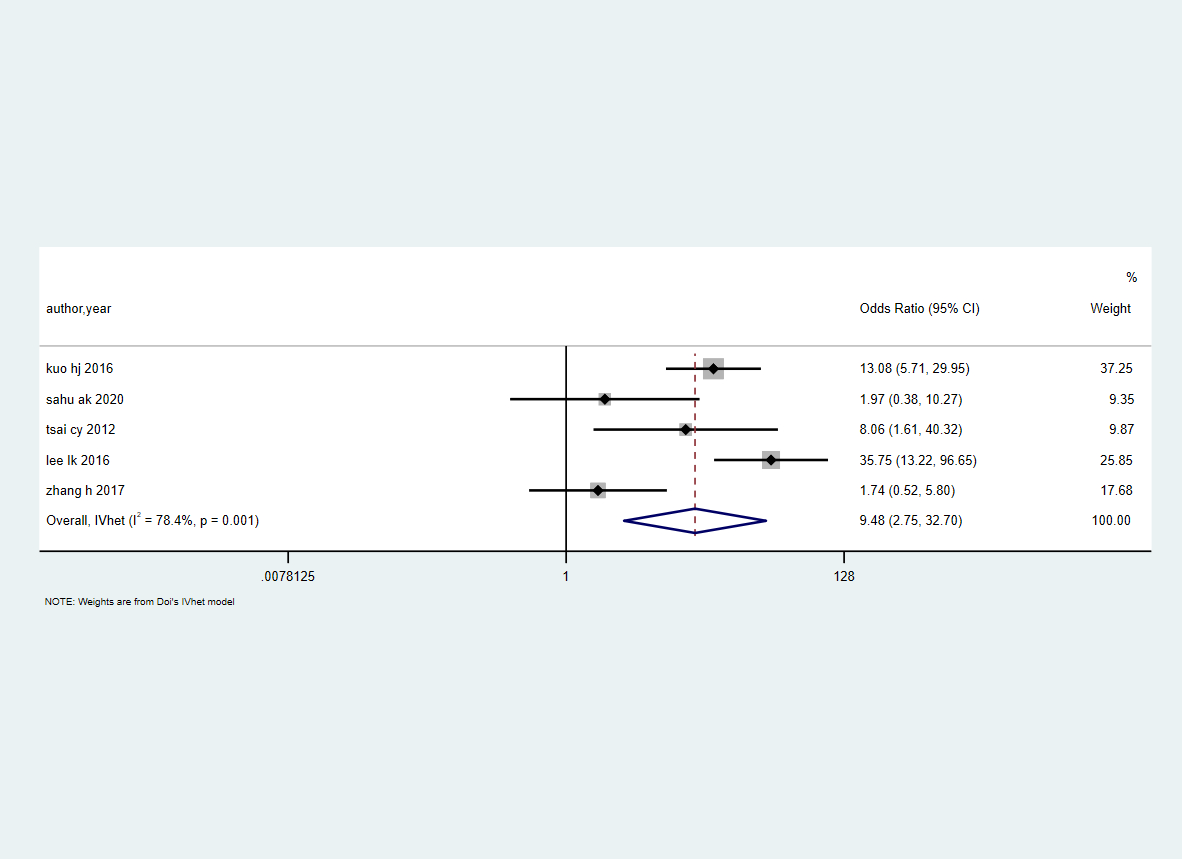

Supplement: Supplementary file 5 — Additional file 5. Forest plots with pooled OR of progression to severe dengue with potential predictors [file 40249_2021_908_MOESM5_ESM.docx]
