# Supplementary material for: Clinical predictors of severe dengue: a systematic review and meta-analysis
Source: Infect Dis Poverty. 2021 Oct 9;10:123. doi: 10.1186/s40249-021-00908-2 (PMC8501593; doi:10.1186/s40249-021-00908-2)
Supplement: Supplementary file 6 — Additional file 6. Subgroup analysis using WHO classification for demography, co-morbidities and clinical warning signs of dengue severity [file 40249_2021_908_MOESM6_ESM.docx]

| Risk factors | WHO classification | Number of studies | Pooled *OR* |  |
| --- | --- | --- | --- | --- |
|  |  |  | (95% *CI*) | *P-value* |
| Children | 1997 | 9 | 2.01 (1.03–3.92) | 0.042 |
|  | 2009 | 13 | 1.84 (1.10–3.07) | 0.019 |
| Female | 1997 | 45 | 1.16 (0.57–2.37) | 0.679 |
|  | 2009 | 69 | 1.27 (0.92–1.75) | 0.147 |
| Secondary Infection | 1997 | 7 | 4.09 (2.22–7.55) | < 0.001 |
|  | 2009 | 22 | 3.09 (2.12–4.49) | < 0.001 |
| Abdominal pain | 1997 | 16 | 2.54 (1.44–4.47) | 0.001 |
|  | 2009 | 39 | 1.66 (1.25–2.19) | < 0.001 |
| Vomiting | 1997 | 12 | 2.59 (1.57–4.27) | < 0.001 |
|  | 2009 | 41 | 1.70 (1.33–2.18) | < 0.001 |
| Hepatomegaly | 1997 | 18 | 10.62 (3.19–35.31) | < 0.001 |
|  | 2009 | 29 | 4.21 (2.63–6.74) | < 0.001 |
| Ascitis | 1997 | 10 | 7.50 (2.67–21.07) | < 0.001 |
|  | 2009 | 12 | 5.83 (3.12–10.89) | < 0.001 |
| Pleural effusion | 1997 | 10 | 4.54 (1.56–13.17) | 0.005 |
|  | 2009 | 15 | 6.70 (3.49–12.85) | < 0.001 |
| Gum bleeding | 1997 | 3 | 1.00 (0.41–2.43) | 0.992 |
|  | 2009 | 9 | 2.70 (0.88–8.26) | 0.081 |
| Epistaxis | 1997 | 4 | 1.58 (1.00–2.49) | 0.052 |
|  | 2009 | 7 | 2.48 (0.53 11.68) | 0.25 |
| Melena | 1997 | 4 | 4.28 (1.80–10.17) | 0.001 |
|  | 2009 | 5 | 3.89 (0.72–20.95) | 0.114 |
