# Supplementary material for: Clinical predictors of severe dengue: a systematic review and meta-analysis
Source: Infect Dis Poverty. 2021 Oct 9;10:123. doi: 10.1186/s40249-021-00908-2 (PMC8501593; doi:10.1186/s40249-021-00908-2)
Supplement: Supplementary file 7 — Additional file 7. Subgroup analysis using age groups for demography and warning signs of severe dengue [file 40249_2021_908_MOESM7_ESM.docx]

| **Predictors** |  | **Studies** | **Pooled *OR*** |  |
| --- | --- | --- | --- | --- |
|  |  |  | **(95% *CI*)** | ***P-value*** |
| **Demography** | | | | |
| Female | Children | 50 | 1.00 (0.88– 1.13) | 0.978 |
|  | Adult | 29 | 2.12 (1.13– 3.97) | 0.019 |
| Secondary Infection | Children | 12 | 3.02 (1.79– 5.12) | < 0.001 |
|  | Adult | 9 | 2.50 (1.57– 3.97) | < 0.001 |
| **Warning signs** | | | | |
| Abdominal pain | Children | 23 | 1.85 (1.29– 2.64) | 0.001 |
|  | Adult | 19 | 1.75 (1.21– 2.54) | 0.003 |
| Vomiting | Children | 19 | 2.05 (1.42– 2.98) | < 0.001 |
|  | Adult | 19 | 1.57 (1.14– 2.16) | 0.006 |
| Hepamegaly | Children | 26 | 3.58 (2.26– 5.66) | < 0.001 |
|  | Adult | 13 | 3.56 (2.03– 6.25) | < 0.001 |
| Ascitis | Children | 15 | 6.14 (3.03– 12.44) | < 0.001 |
|  | Adult | 4 | 5.40 (2.40– 12.13) | < 0.001 |
| Pleural effusion | Children | 13 | 5.48 (3.28– 9.15) | < 0.001 |
|  | Adult | 7 | 4.22 (0.62– 28.78) | 0.141 |
