# Supplementary material for: Clinical predictors of severe dengue: a systematic review and meta-analysis
Source: Infect Dis Poverty. 2021 Oct 9;10:123. doi: 10.1186/s40249-021-00908-2 (PMC8501593; doi:10.1186/s40249-021-00908-2)
Supplement: Supplementary file 8 — Additional file 8. Sensitivity analysis [file 40249_2021_908_MOESM8_ESM.docx]

| **Predictors** | **Number of Studies** | **Pooled *OR* (95% *CI*)** |
| --- | --- | --- |
| **Demography** | | |
| Children | 22 | 1.85 (1.30, 2.64) |
| Female | 114 | 1.17 (0.90, 1.53) |
| Secondary Infection | 29 | 3.18 (2.37, 4.26) |
| **Co-morbidities** | | |
| Diabetes | 10 | 2.96 (1.83, 4.82) |
| CVD | 4 | 2.61 (0.45, 15.14) |
| Obesity | 5 | 0.78 (0.42, 1.43) |
| Renal disease | 4 | 4.46 (1.53, 13.00) |
| Hypertension | 9 | 1.83 (0.98, 3.39) |
| **Warning signs** | | |
| ↑Hct & ↓Plt | 7 | 4.86 (1.61, 14.67) |
| Abdominal pain | 55 | 2.11 (1.64, 2.71) |
| Vomiting | 53 | 1.94 (1.58, 2.39) |
| Lethargy | 10 | 3.06 (1.30, 7.17) |
| Hepatomegaly | 47 | 5.41 (3.32, 8.82) |
| Ascitis | 22 | 6.00 (3.69, 9.76) |
| Pleural effusion | 25 | 6.18 (3.65, 10.45) |
| Gum bleeding | 12 | 2.06 (0.90, 4.71) |
| Epistaxis | 11 | 1.95 (0.83, 4.60) |
| Hemetemesis | 5 | 12.40 (4.99, 30.84) |
| Melena | 9 | 4.38 (1.84, 10.42) |
| Skin bleeding | 4 | 1.55 (0.59, 4.04) |
| GI bleeding | 5 | 9.54 (2.79, 32.60) |
