# Supplementary material for: Clinical predictors of severe dengue: a systematic review and meta-analysis
Source: Infect Dis Poverty. 2021 Oct 9;10:123. doi: 10.1186/s40249-021-00908-2 (PMC8501593; doi:10.1186/s40249-021-00908-2)
Supplement: Supplementary file 9 — Additional file 9. Doi plots and LFK values used to assess publication bias [file 40249_2021_908_MOESM9_ESM.docx]

| Additional file 9 - Age group 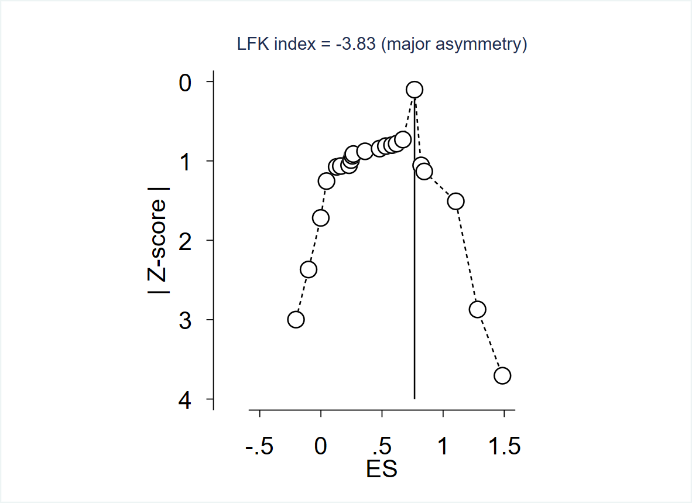 | Additional file 9 - Sex 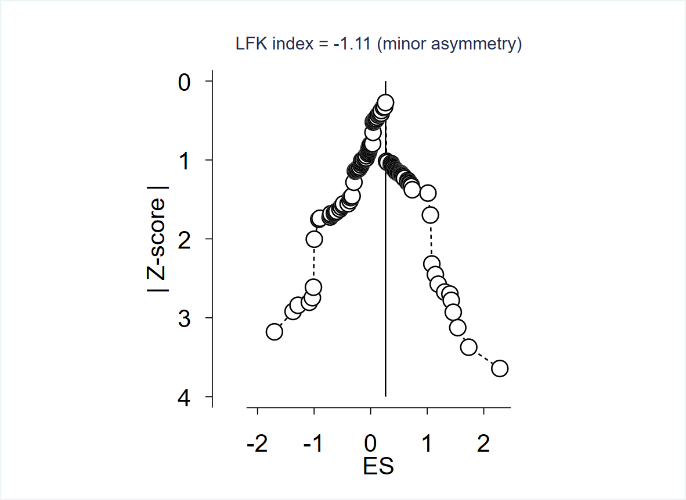 |
| --- | --- |
| Additional file 9 - Secondary infection 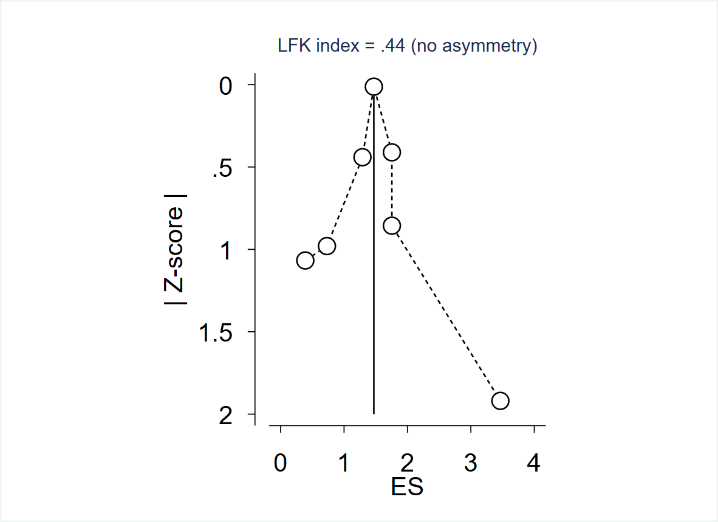 | Additional file 9 - Diabetes *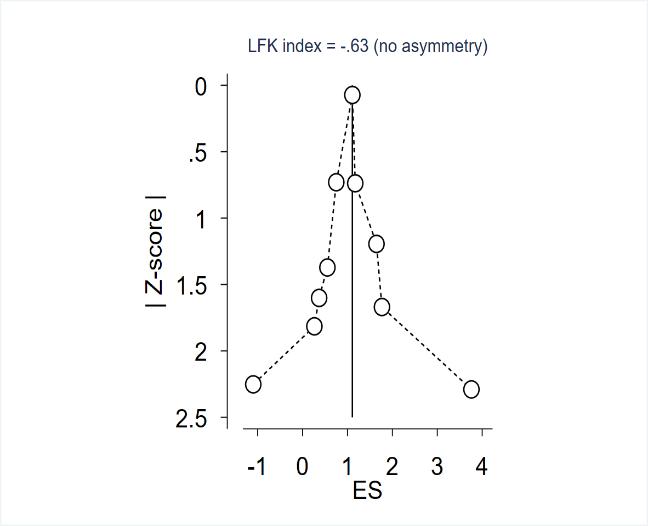* |
| Additional file 9 – Cardiovascular disease**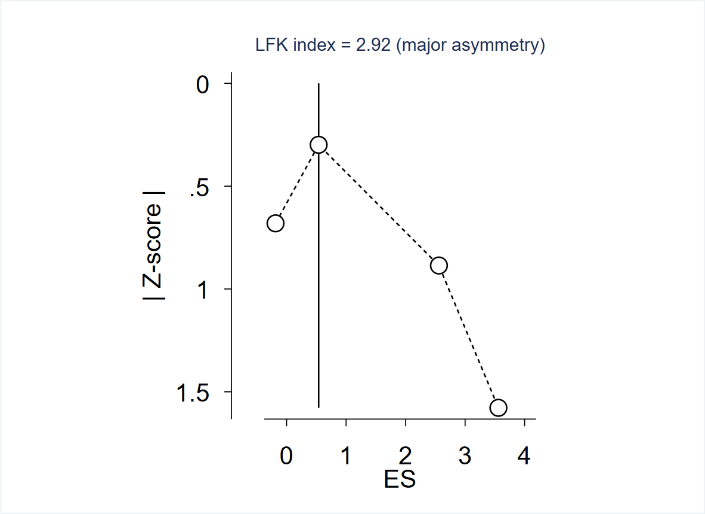** | Additional file 9 - Obesity  **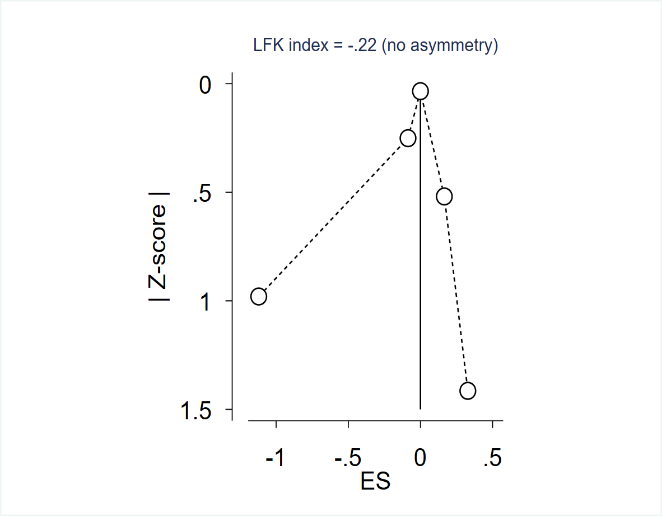** |

| Additional file 9 - Renal disease**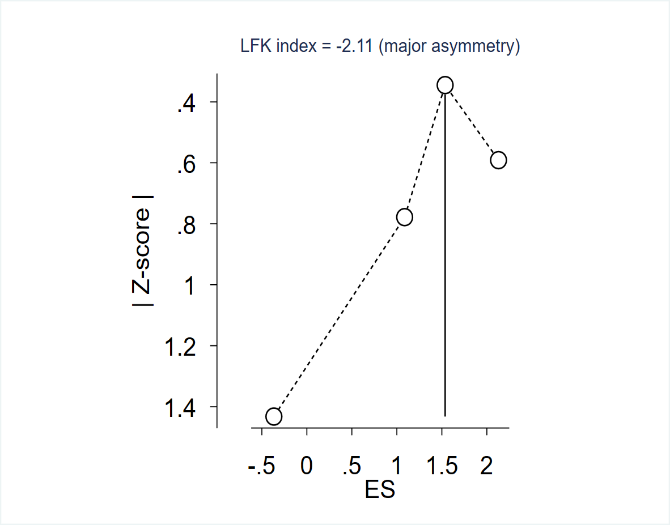** | Additional file 9 - Hypertension**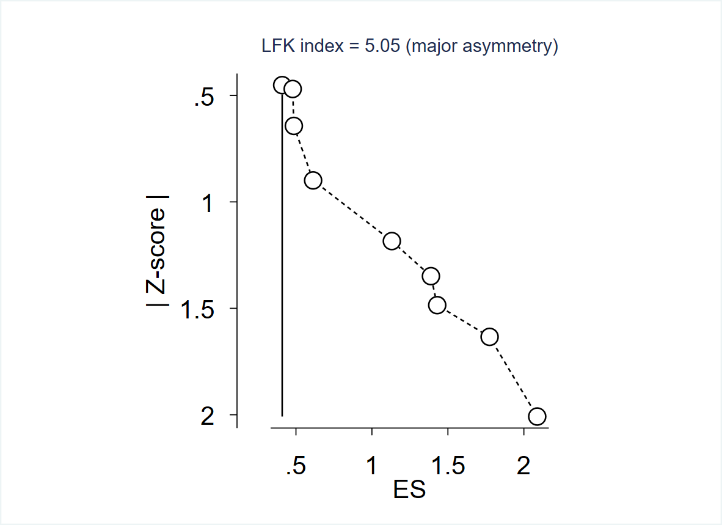** |
| --- | --- |
| Additional file 9 - ↑Hct & ↓Plt **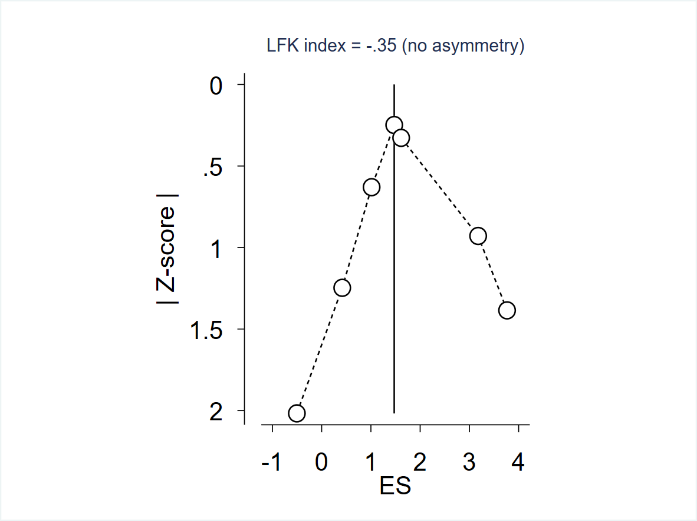** | Additional file 9 - Abdominal pain **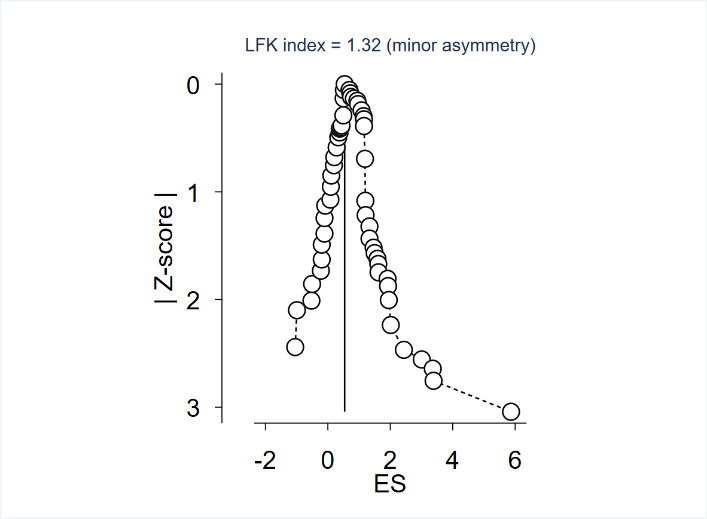** |
| Additional file 9 - Vomiting**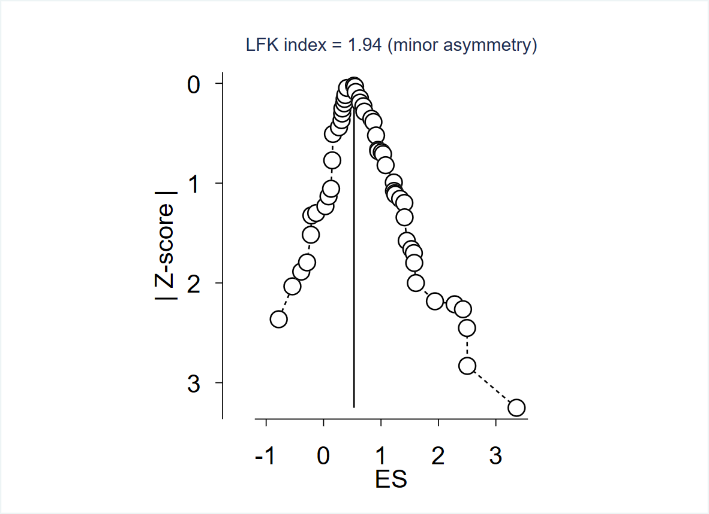** | Additional file 9 - Lethargy  **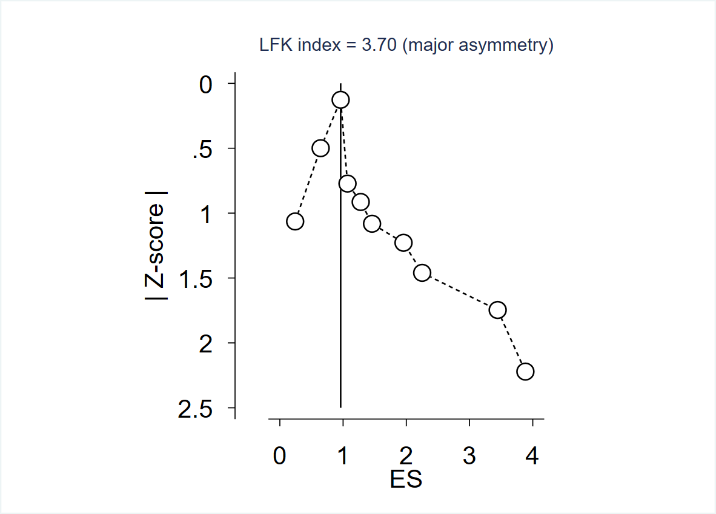** |
|  |  |
|  |  |

| Additional file 9 - Enlarged liver size**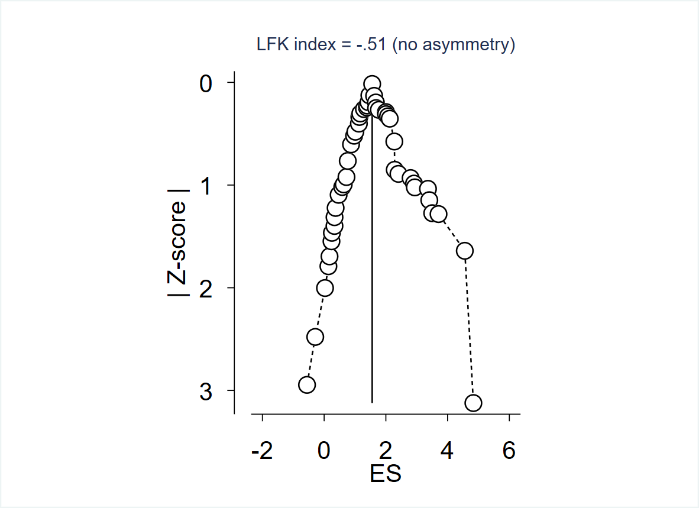** | Additional file 9 - Ascitis**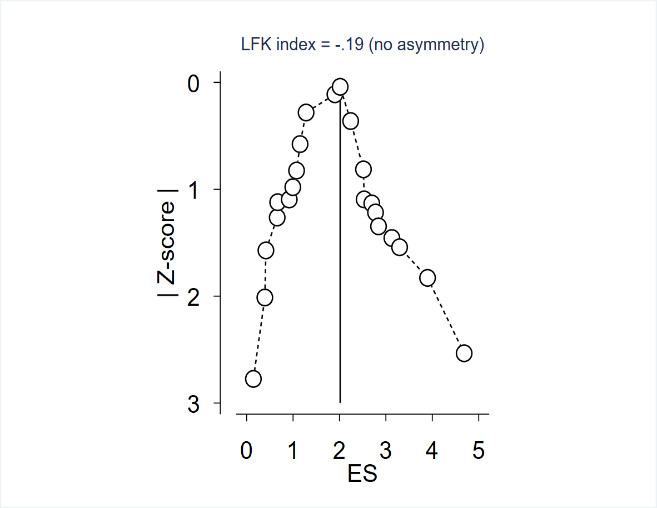** |
| --- | --- |
| Additional file 9 - Pleural effusion**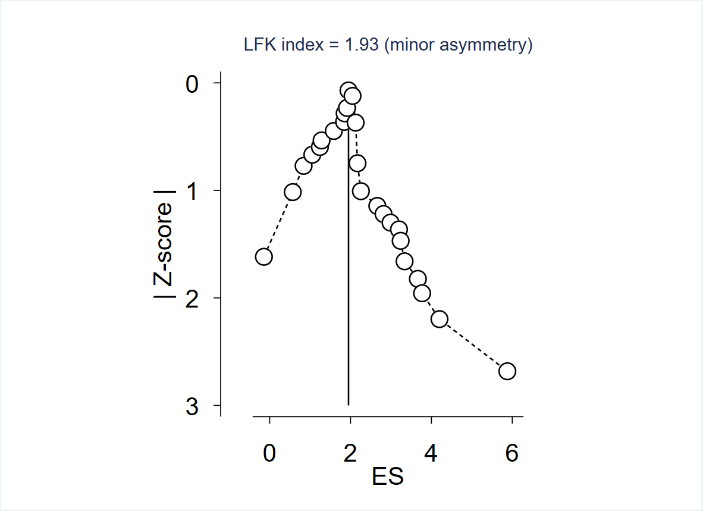** | Additional file 9 - Gum bleeding**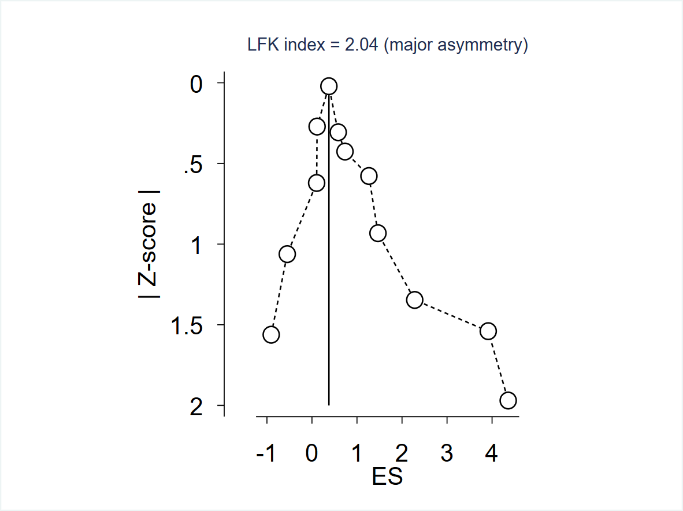** |
| Additional file 9 - Epistaxis**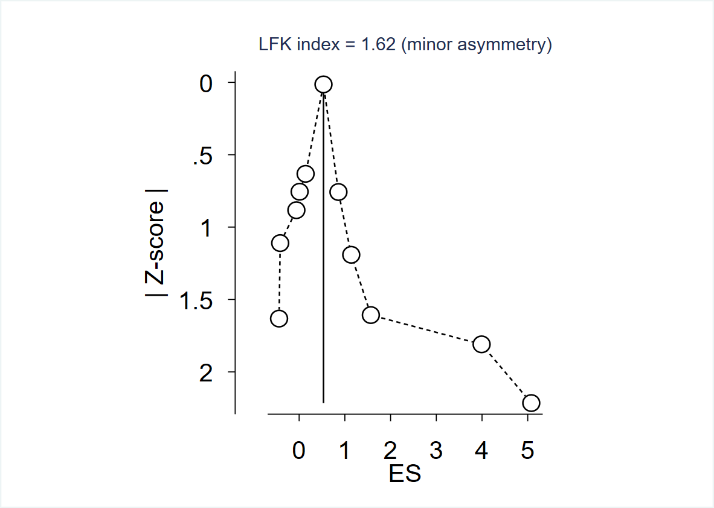** | Additional file 9 - Hemetemesis  **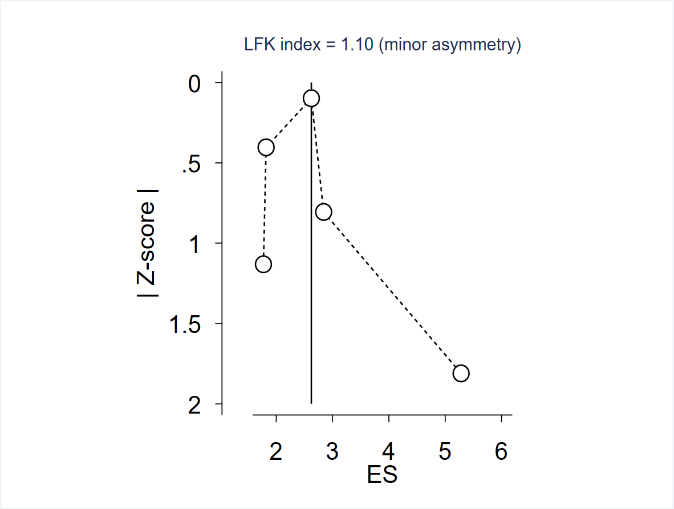** |

| Additional file 9 - Melena**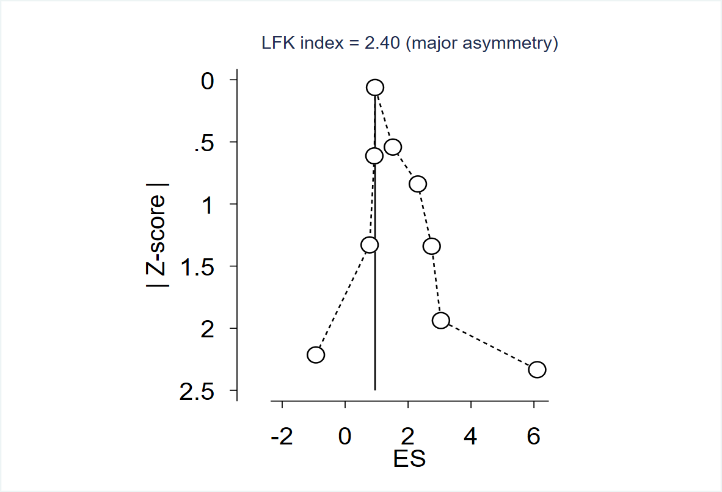** | Additional file 9 - Skin bleeding**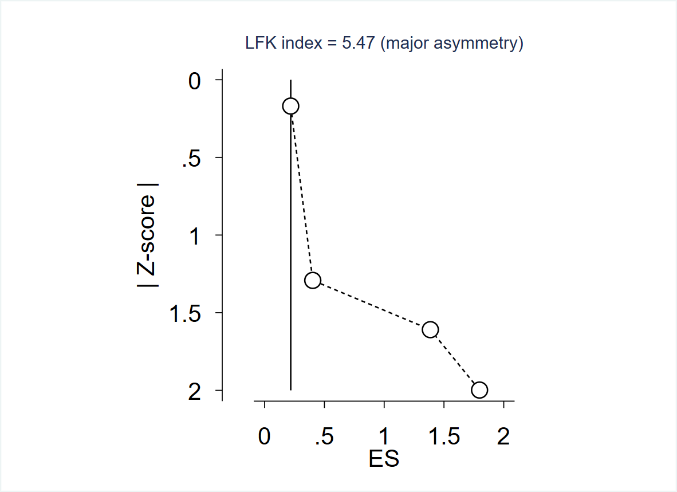** |
| --- | --- |
| Additional file 9 - GI bleeding**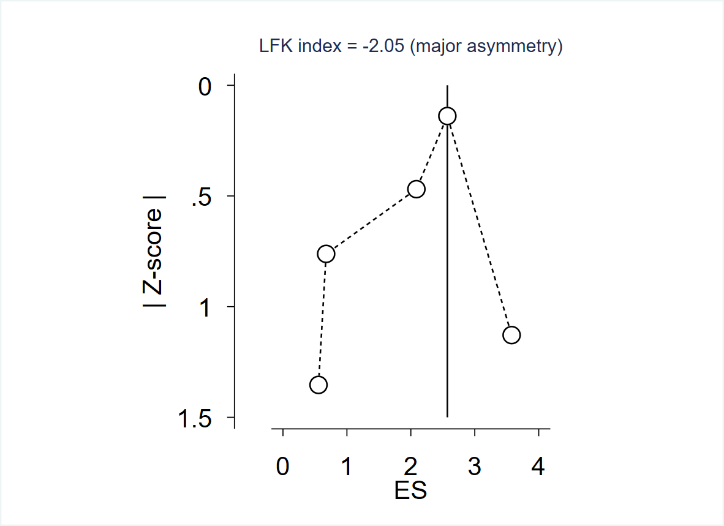** |  |
